# Maternal and fetal genetic contribution to gestational weight gain

**DOI:** 10.1038/ijo.2017.248

**Published:** 2017-11-21

**Authors:** N M Warrington, R Richmond, B Fenstra, R Myhre, R Gaillard, L Paternoster, C A Wang, R N Beaumont, S Das, M Murcia, S J Barton, A Espinosa, E Thiering, M Atalay, N Pitkänen, I Ntalla, A E Jonsson, R Freathy, V Karhunen, C M T Tiesler, C Allard, A Crawford, S M Ring, M Melbye, P Magnus, F Rivadeneira, L Skotte, T Hansen, J Marsh, M Guxens, J W Holloway, H Grallert, V W V Jaddoe, W L Lowe Jr, T Roumeliotaki, A T Hattersley, V Lindi, K Pahkala, K Panoutsopoulou, M Standl, C Flexeder, L Bouchard, E Aagaard Nohr, L Santa Marina, M Kogevinas, H Niinikoski, G Dedoussis, J Heinrich, R M Reynolds, T Lakka, E Zeggini, O T Raitakari, L Chatzi, H M Inskip, M Bustamante, M-F Hivert, M-R Jarvelin, T I A Sørensen, C Pennell, J F Felix, B Jacobsson, F Geller, D M Evans, D A Lawlor

**Affiliations:** 1 University of Queensland Diamantina Institute, Translational Research Institute, Brisbane, Queensland, Australia; 2 Division of Obstetrics and Gynaecology, The University of Western Australia, Perth, Western Australia, Australia; 3 Medical Research Council Integrative Epidemiology Unit at the University of Bristol, Bristol, UK; 4 Population Health Science, Bristol Medical School, University of Bristol, Bristol, UK; 5 Department of Epidemiology Research, Statens Serum Institute, Copenhagen, Denmark; 6 Norwegian Institute of Public Health, Oslo, Norway; 7 The Generation R Study Group, Erasmus MC, University Medical Center Rotterdam, Rotterdam, The Netherlands; 8 Department of Epidemiology, Erasmus MC, University Medical Center Rotterdam, Rotterdam, The Netherlands; 9 Department of Pediatrics, Erasmus MC, University Medical Center Rotterdam, Rotterdam, The Netherlands; 10 Institute of Biomedical and Clinical Science, University of Exeter Medical School, University of Exeter, Royal Devon and Exeter Hospital, Exeter, UK; 11 Department of Public Health and Primary Care, School of Public Health, Imperial College London, London, UK; 12 Epidemiology and Environmental Health Joint Research Unit, FISABIO–Universitat Jaume I–Universitat de València, Valencia, Spain; 13 Spanish Consortium for Research on Epidemiology and Public Health (CIBERESP), Spain; 14 MRC Lifecourse Epidemiology Unit, Faulty of Medicine, University of Southampton, Southampton, UK; 15 ISGlobal, Centre for Research in Environmental Epidemiology (CREAL), Barcelona, Spain; 16 IMIM (Hospital del Mar Medical Research Institute), Barcelona, Spain; 17 Universitat Pompeu Fabra (UPF), Barcelona, Spain; 18 Institute of Epidemiology I, Helmholtz Zentrum München- German Research Center for Environmental Health, Neuherberg, Germany; 19 Division of Metabolic and Nutritional Medicine, Dr. von Hauner Children's Hospital, University of Munich Medical Center, Munich, Germany; 20 Institute of Biomedicine, School of Medicine, University of Eastern Finland, Kuopio, Finland; 21 Research Centre of Applied and Preventive Cardiovascular Medicine, University of Turku, Turku, Finland; 22 William Harvey Research Institute, Barts and the London School of Medicine and Dentistry, Queen Mary University of London, London, UK; 23 The Novo Nordisk Foundation Center for Basic Metabolic Research, Section of Metabolic Genetics, and Department of Public Health, Faculty of Health and Medical Sciences, University of Copenhagen, Copenhagen, Denmark; 24 Center for Life Course Health Research, Faculty of Medicine, University of Oulu, Oulu, Finland; 25 Biocenter Oulu, University of Oulu, Oulu, Finland; 26 Centre de Recherche du Centre Hospitalier Universitaire de Sherbrooke, Sherbrooke, Canada; 27 British Heart Foundation Centre for Cardiovascular Science, Queen's Medical Research Institute, University of Edinburgh, Edinburgh, UK; 28 ALSPAC (Children of the 90s), School of Social and Community Medicine, University of Bristol, Bristol, UK; 29 Department of Clinical Medicine, Copenhagen University, Copenhagen, Denmark; 30 Department of Medicine, Stanford School of Medicine, Stanford, CA, USA; 31 Department of Internal Medicine, Erasmus MC, University Medical Center Rotterdam, Rotterdam, The Netherlands; 32 Department of Child and Adolescent Psychiatry/Psychology, Erasmus University Medical Centre–Sophia Children’s Hospital, Rotterdam, The Netherlands; 33 Human Development and Health, Faculty of Medicine, University of Southampton, Southampton, UK; 34 Institute of Epidemiology II, Research Unit of Molecular Epidemiology, Helmholtz Zentrum München Research Center for Environmental Health, Neuherberg, Germany; 35 German Center for Diabetes Research (DZD), Neuherberg, Germany; 36 Clinical Cooperation Group Type 2 Diabetes, Helmholtz Zentrum München, Neuherberg, Germany; 37 Clinical Cooperation Group Nutrigenomics and Type 2 Diabetes, Helmholtz Zentrum München, Neuherberg, Germany; 38 Technische Universität München, Freising, Germany; 39 Department of Medicine, Division of Endocrinology, Metabolism, and Molecular Medicine, Feinberg School of Medicine, Northwestern University, Chicago, IL, USA; 40 Department of Social Medicine, University of Crete, Crete, Greece; 41 Paavo Nurmi Centre, Sports and Exercise Medicine Unit, Department of Health and Physical Activity, Turku, Finland; 42 Department of Human Genetics, Wellcome Trust Sanger Institute, Hinxton, UK; 43 Department of Biochemistry, Faculty of medicine and life sciences, Université de Sherbrooke, Sherbrooke, Canada; 44 Public Health Division of Gipuzkoa, Basque Government, Vitoria-Gasteiz, Spain; 45 Health Research Institute, Biodonostia, San Sebastián, Gipuzkoa, Spain; 46 Health Research Institute, Biodonostia, San Sebastián, Spain; 47 Department of Pediatrics, Turku University Hospital, Turku, Finland; 48 Department of Nutrition and Dietetics, Harokopio University of Athens, Athens, Greece; 49 Institute and Outpatient Clinic for Occupational, Social and Environmental Medicine, Inner City Clinic, University Hospital Munich, Ludwig Maximilian University of Munich, Munich, Germany; 50 Department of Clinical Physiology and Nuclear Medicine, Kuopio University Hospital, School of Medicine, University of Eastern Finland, Kuopio, Finland; 51 Kuopio Research Institute of Exercise Medicine, Kuopio, Finland; 52 Department of Clinical Physiology and Nuclear Medicine, Turku University Hospital, Turku, Finland; 53 Department of Preventive Medicine, Keck School of Medicine, University of Southern California, Los Angeles, CA, USA; 54 Department of Social Medicine, University of Crete, Crete, Greece; 55 Department of Genetics and Cell Biology, Faculty of Health, Medicine and Life Sciences, Maastricht University, Maastricht, The Netherlands; 56 NIHR Southampton Biomedical Research Centre, University of Southampton and University Hospital Southampton NHS Foundation Trust, Southampton, UK; 57 Centre for Genomic Regulation (CRG), The Barcelona Institute of Science and Technology, Barcelona, Spain; 58 Department of Population Medicine at Harvard Pilgrim Health Care Institute, Harvard Medical School, Boston, MA, USA; 59 Diabetes Unit, Massachusetts General Hospital, Boston, MA, USA; 60 Department of Epidemiology and Biostatistics, MRC–PHE Centre for Environment and Health, School of Public Health, Imperial College London, London, UK; 61 Unit of Primary Care, Oulu University Hospital, Oulu, Finland; 62 Department of Clinical Epidemiology (formally the Institute of Preventive Medicine), Bispebjerg and Frederiksberg Hospital, The Capital Region, Copenhagen, Denmark; 63 Department of Obstetrics and Gynecology, Sahlgrenska Academy, Gothenburg University, Gothenburg, Sweden; 64 Department of Genetics and Bioinformatics, Domain of Health Data and Digitalization, Institute of Public Health, Oslo, Norway

## Abstract

**Background:**

Clinical recommendations to limit gestational weight gain (GWG) imply high GWG is causally related to adverse outcomes in mother or offspring, but GWG is the sum of several inter-related complex phenotypes (maternal fat deposition and vascular expansion, placenta, amniotic fluid and fetal growth). Understanding the genetic contribution to GWG could help clarify the potential effect of its different components on maternal and offspring health. Here we explore the genetic contribution to total, early and late GWG.

**Participants and methods:**

A genome-wide association study was used to identify maternal and fetal variants contributing to GWG in up to 10 543 mothers and 16 317 offspring of European origin, with replication in 10 660 mothers and 7561 offspring. Additional analyses determined the proportion of variability in GWG from maternal and fetal common genetic variants and the overlap of established genome-wide significant variants for phenotypes relevant to GWG (for example, maternal body mass index (BMI) and glucose, birth weight).

**Results:**

Approximately 20% of the variability in GWG was tagged by common maternal genetic variants, and the fetal genome made a surprisingly minor contribution to explain variation in GWG. Variants near the pregnancy-specific beta-1 glycoprotein 5 (*PSG5*) gene reached genome-wide significance (*P*=1.71 × 10^−8^) for total GWG in the offspring genome, but did not replicate. Some established variants associated with increased BMI, fasting glucose and type 2 diabetes were associated with lower early, and higher later GWG. Maternal variants related to higher systolic blood pressure were related to lower late GWG. Established maternal and fetal birth weight variants were largely unrelated to GWG.

**Conclusions:**

We found a modest contribution of maternal common variants to GWG and some overlap of maternal BMI, glucose and type 2 diabetes variants with GWG. These findings suggest that associations between GWG and later offspring/maternal outcomes may be due to the relationship of maternal BMI and diabetes with GWG.

## Introduction

High and low levels of gestational weight gain (GWG), defined as the weight a woman gains during pregnancy before delivery of her infant,^1^ are associated with a wide range of adverse outcomes for mother and child in the short term (during pregnancy and the perinatal period), and long term.^2, 3, 4, 5, 6, 7, 8, 9^ As a consequence of these associations, recommendations for healthy GWG are increasingly used in antenatal care,^1^ despite a lack of evidence that any of these associations are causal, and if they are, what the mechanisms underlying them might be.^3, 10^ GWG is a complex phenotype that is influenced by maternal responses to pregnancy, such as gestational fat deposition and volume expansion, as well as fetal growth, placental size and amniotic fluid volume.^3, 10^ Each of these are likely to be influenced both by maternal and fetal genes and environmental exposures. Understanding the maternal and fetal genetic contributions to GWG could shed light on both genetic and non-genetic contributions to between woman variation in GWG.^11, 12^ For example, we have recently used maternal genetic instrumental variables to determine the causal effect of maternal pregnancy adiposity and related traits on offspring birth weight and ponderal index.^13^

Among 1159 European origin Swedish maternal twin pairs (694 pairs with data on their first pregnancies and 465 on their second), it has been shown that approximately 40% of variability in first pregnancy GWG was due to genetic factors.^14^ Other studies have examined the associations of candidate maternal and/or fetal adiposity or diabetes related genetic variants with GWG and yielded inconsistent results; however, these studies have had small sample sizes, been conducted in single studies and have not sought independent replication.^15, 16, 17^ To our knowledge, no previous genome-wide association study (GWAS) of GWG has been conducted.

The aim of this study was to increase understanding of the genetic and non-genetic determinants of GWG by (a) estimating the proportion of variation in total, early and late GWG tagged by maternal and fetal common genetic variants; (b) undertaking a GWAS of maternal and fetal genetic variants with total, early and late GWG, and attempting to replicate associations in independent samples and (c) determining the associations of genetic variants from GWAS of phenotypes that are plausible contributors to GWG (that is, birth weight, body mass index (BMI), waist-hip ratio, height, blood pressure, glucose, type 2 diabetes and vitamin D) with total, early and late GWG. We examined associations of maternal and fetal genetic exposures with total, early and late GWG, because the relative contribution of maternal and fetal phenotypes to GWG vary across gestation. For example, maternal fat deposition contributes relatively more to early GWG (up to ~18–20 weeks of gestation), and fetal growth more to later GWG.^3, 10^ We included vitamin D (25(OH)D) as a phenotype that plausibly contributes to GWG as maternal 25(OH)D may have a positive affect on birth weight,^13^ and therefore may have a positive association with GWG.

## Materials and methods

We included singleton pregnancies of mother–offspring pairs of European origin from 20 pregnancy/birth cohorts, described in detail in Supplementary material and Supplementary Table 1. Pregnancies that resulted in a miscarriage or stillbirth, those with a known congenital anomaly and those where delivery was preterm (before 37 completed weeks of gestation) were excluded.

Total GWG was defined as the last gestational weight (as long as this was ⩾28 weeks of gestation) before delivery minus pre-/early-pregnancy weight divided by the length of gestation in weeks at the last measurement. Pre/early-pregnancy weight was defined as maternal self-reported pre-pregnancy weight (with this report collected during pregnancy), a research/clinical measure of weight before pregnancy (with that measure taken no >12 weeks before predicted date of conception) or the first antenatal clinic weight (with that assessment ⩽13 weeks of gestation), which ever was the earliest. Early GWG was the difference between pre-/early-pregnancy weight and weight measured any time between 18 and 20 (inclusive) completed weeks of gestation divided by length of gestation in weeks at the time of the 18- to 20-week measurement. Late GWG was the difference between the 18- and 20-week measurement and the last gestational weight measure at ⩾28 weeks of gestation divided by the gestational age difference in completed weeks between these two measurements. Although we were interested in the possibility that different maternal and fetal genetic variants might contribute to early and late GWG (as discussed in the final paragraph of the introduction), we considered total GWG to be our main outcome. The rationale for this was: (a) this was the phenotype for which we had most data and therefore greatest statistical power; (b) the majority of epidemiological studies relating GWG to subsequent maternal and offspring outcomes use total GWG;^2, 3, 7, 18^ and (c) recommendations regarding optimal GWG use total GWG.^1^ Similarly, the gestational ages used to define early and late GWG were based several factors: (a) maximizing sample sizes for both early and late GWG, which for many studies reflected times in pregnancy when women are routinely weighed; (b) evidence that the relative (to maternal contribution) fetal contribution to GWG becomes greater from 16 to 20 weeks of gestation;^1, 19^ and (c) applying multilevel models to the very detailed repeat measurements of gestational weight in the Avon Longitudinal Study of Parents and Children (ALSPAC), in which the median (interquartile range) of measurements per woman was 12,^9, 10, 11, 12, 13^ demonstrated changes in the amount of weight gained per week of gestation at 18 and 28 weeks.^4, 20, 21^ Other epidemiological studies report differences in GWG from around 14 to 15 weeks of pregnancy, but as discussed in previous reviews, this if often driven by selecting measures around traditional trimesters (14 weeks being the time at which the second trimester begins).^1, 2^ In studies with repeated measurements between 18 and 20 weeks, the one nearest to 18 weeks was used and in those with repeated later measurements the last weight was defined as the one nearest to (but before) delivery. Total, early and late GWG s.d. (z-) scores were calculated within each study as the participant value minus the individual study mean then divided by the study s.d.

### Proportion of variation in total, early and late GWG, and birth weight that is due to maternal and fetal common genetic variants

We used methods that have been developed for use with genome-wide data to estimate the proportion of variation in total, early and late GWG, and birth weight tagged by maternal and fetal common genetic variants. Genetic restricted maximum likelihood (GREML)^22^ and maternal genome-wide complex trait analysis (M-GCTA)^23^ were applied to maternal and fetal genome-wide data from ALSPAC. The M-GCTA model uses similarity between mothers and offspring in the genome-wide data to partition the phenotypic variance in GWG into components due to the maternal genotype, the child’s genotype, the covariance between the maternal and child’s genotype and an environmental component.

ALSPAC is a prospective population-based birth cohort study that recruited 14 541 pregnant women resident in Avon, UK with expected dates of delivery between 1 April 1991 and 31 December 1992 (http://www.alspac.bris.ac.uk).^24, 25^ GWG was determined using data extracted from obstetric medical records by trained research midwives.^5^ Birth weight, gestational age (in completed weeks) and fetal sex were obtained from obstetric/perinatal records. Maternal genome-wide data were obtained from the genome-wide Illumina 660K quad single nucleotide polymorphism (SNP) chip (Illumina Inc., San Diego, CA, USA) at the Centre National de Genotypage, Paris. Fetal genome-wide data were obtained from the Illumina HumanHap550 quad genome-wide SNP genotyping platform by 23 and Me subcontracting the Wellcome Trust Sanger Institute, Cambridge, UK, and the Laboratory Corporation of America, Burlington, NC, USA. Further details, including genotype imputation and QC, are provided in online Supplementary material and characteristics of the participants are described in Supplementary Table 1.

### Maternal and fetal GWAS of total, early and late GWG

All studies are in the Early Growth Genetics (EGG) consortium (http://egg-consortium.org/) with relevant data participated. Twenty independent pregnancy/birth cohorts contributed to at least one discovery and/or replication analysis (this included data from ALSPAC). Details of each of these studies are provided in the Supplementary Material and study participant characteristics, including their contribution to each GWAS, are shown in Supplementary Table 1. For total GWG, up to 10 543 (including participants from ALSPAC, DNBC-GOYA, DNBC-PTB and MoBa; see Supplementary material for details of these cohorts) and 16 317 participants (from ALSPAC, DNBC-PTB, Generation R, INMA, LISAplus, MoBa, NFBC-1966, PANIC, Raine, STRIP and TEENAGE; Supplementary material) contributed to maternal and fetal discovery GWAS, respectively (Supplementary Table 1 and Table 1). The numbers for early and late GWG GWAS being somewhat lower (Table 1). Up to an additional 10 660 and 7561 participants contributed to maternal and fetal replication analyses (see Supplementary Table 1 for cohorts included), with the maximum total meta-analysis sample size for total GWG being 18 420 and 21 105 for maternal and fetal GWAS, respectively.

All genotyping was undertaken by laboratory staff at each of the contributing studies who were blinded to any phenotypic data. GWAS discovery and replication analyses were undertaken independently by analysts working with each of the contributing studies following a prior agreed analysis plan. Genotypic data imputed to HapMap Phase 2 (Build 36, release 22) was used (methods for imputing within each contributing study is described in the Supplementary Material) in the analysis, assuming an additive genetic model and adjusting for fetal sex.

Fixed-effects, inverse-variance weighted meta-analyses in METAL^26^ were undertaken to combine GWAS results from the individual discovery studies. The most significant SNPs in regions reaching suggestive significance (*P*<10^−5^) in the discovery GWAS of any of the analyses (that is, total, early or late GWG or in the maternal or fetal genome) were taken forward to replication. This set of SNPs were analyzed against the three phenotypes in the replication studies and the results were combined using inverse-variance weighted meta-analysis in R (version 3.0.0) using the rmeta package.^27^ In addition, to investigate whether this set of top SNPs were more likely to be acting in the maternal or offspring genome to influence GWG, conditional analysis was conducted in studies where both maternal and fetal genotype were available. Again, the results from these analyses were combined using inverse-variance weighted meta-analysis in R (version 3.0.0). Computer code for the discovery and replication meta-analyses is available from the first author upon request.

### Maternal and fetal genetic variants for phenotypes with plausible contributions to GWG

We examined the associations of a set of *a priori* agreed genetic variants that had previously (in GWAS) been shown to be robustly associated with phenotypes that might plausibly influence GWG with our GWG phenotypes. These were genetic variants for birth weight, BMI, waist-hip ratio, height, blood pressure, fasting glucose, type 2 diabetes and vitamin D (25(OH)D). Supplementary Table 2 lists the variants included for each of the traits. The results for each of the variants were extracted from the discovery GWAS, and the replication studies provided results for the subset of variants they had available. Inverse-variance weighted meta-analysis was conducted in METAL^26^ to combine the results across all the cohorts. In these hypothesis-driven analyses, we use a two-sided *P*-value of <0.05 as indicating statistical significance.

## Results

Total GWG was between 0.35 and 0.45 kg per week in all of the general population studies and somewhat lower in the two studies that combined severely obese women with lean or a population cohort comparison group; in all studies early GWG was considerably lower than late GWG (Supplementary Table 1). The correlation between early (defined as between pre-pregnancy and 18 weeks) and late GWG (18 weeks to delivery) and between early and total GWG, in one of the largest contributing studies with data on both periods (ALSPAC) were −0.08 and −0.11, respectively.

### Proportion of variation in GWG and birth weight because of maternal and fetal common genetic variants

SNPs across the genome explained broadly similar proportions of variation in late GWG and early GWG, but with stronger contributions of maternal compared with fetal genome, with SNPs in the maternal genome explaining approximately twice the amount of variation in total GWG than the fetal genome (Table 2). The opposite pattern was seen for birth weight, for example, SNPs across the maternal genome explained 24% (*P*=1.94 × 10^−6^) of the variation in total GWG, with 12% (*P*=0.008) explained by SNPs in the fetal genome, whereas the maternal genome explained 13% (*P*=0.02) and fetal genome 18% (*P*=1.86 × 10^−3^) of variation in birth weight (Table 2). When we modeled maternal and fetal contributions together this pattern remained, but with the differences between maternal and fetal contributions increasing somewhat; for total GWG 17% and 5%, respectively, for maternal and fetal genome and for birth weight 4% and 24%, respectively, for maternal and fetal genome, with relatively little covariance between the two genomes for either trait. When the covariance and offspring/maternal variance components were constrained to zero in the M-GCTA model, similar results to the GREML analysis were obtained (Supplementary Table 3).

### Maternal and fetal GWAS of total, early and late GWG

There was no systematic inflation of the test statistics in the meta-analysis of approximately 2.5 million SNPs (λ_mat-early_=1.01, λ_mat-late_=1.01, λ_mat-total_=1.02, λ_off-early_=1.02, λ_off-late_=1.00, λ_off-total_=1.00; Supplementary Figure 1). In discovery analyses, one variant, rs16989175 near the pregnancy-specific beta-1 glycoprotein 5 (*PSG5*) gene, reached conventional GWAS significance (P<5 × 10^−8^) for fetal genetic association with total GWG; it also showed some evidence of fetal association with late (*P*=2.4 × 10^−3^) and early (*P*=0.02) GWG. However, it did not replicate in either the maternal or fetal analysis. An additional nine regions were identified as being suggestively significant (*P*< 10^−5^) for at least one phenotype in either maternal or fetal genome (Supplementary Figure 2). These were taken forward to replication analyses. Only one of these 10 SNPs replicated, rs310087 near *SYT4* (Table 1). This SNP was associated with total GWG in the fetal genome (mean difference in total GWG per allele 0.06 (95% confidence interval (CI): 0.04, 0.08) kg per week; *P*=3 × 10^−6^ in discovery samples and 0.05 (95% CI: 0.01, 0.09) kg per week; *P*=0.03 in replication samples and 0.06 (95% CI: 0.04, 0.08) kg per week; *P*=1.6 × 10^−5^ in pooled discovery and replication). For 6 out of the 10 top SNPs identified (rs481396, rs3924699, rs6457375, rs13295979, rs1702200 and rs7133083), the point estimate was larger for the maternal genotype on total GWG than the offspring genotype on this phenotype (Table 1). This was suggested in conditional analyses, whereby the point estimates for the offspring genotypes mostly attenuated after adjusting for maternal genotype (Table 3). The variant near *SYT4*, that in the fetal genome was nominally significantly associated with total GWG in discovery analyses and replicated, was not notably altered with adjustment for the maternal variant. Summary statistics from the discovery meta-analyses are available at http://egg-consortium.org/.

### Association of maternal and fetal genotypes for phenotypes with plausible contributions with GWG

Seven of the 32 BMI-associated SNPs showed evidence of association (*P*<0.05) with early GWG using maternal genotype, with five of the BMI increasing alleles associated with a decrease in early GWG (Supplementary Figure 3). In contrast, only three of the BMI-associated SNPs showed association with late GWG using maternal genotype, and all three increased both BMI and GWG. A similar pattern of association was seen with the SNPs associated with glucose (Supplementary Figure 4) and type 2 diabetes (Supplementary Figure 5), whereby alleles associated with increased glucose/risk of type 2 diabetes showed evidence of association of maternal genotype with decreased GWG in early gestation and with increased GWG in late gestation. A smaller portion of SNPs for these phenotypes using the offspring genotype were associated with GWG (Supplementary Figures 3 to 5).

Surprisingly, none of the birth weight-associated SNPs using the offspring genotype were associated with any GWG phenotype (Supplementary Figure 6). However, the SNP with the largest effect on birth weight (from fetal GWAS of birth weight), rs900400, using the maternal genotype was associated with decreased late GWG and total GWG, for each birth weight increasing allele. SNPs associated with blood pressure, when using the maternal genome, showed stronger association with late GWG than early GWG, with the blood pressure increasing allele for most variants associating with decreased GWG (Supplementary Figure 7). Offspring blood pressure SNPs and SNPs associated with waist-hip ratio (maternal or offspring; Supplementary Figure 8), vitamin D (25(OH)D) (Supplementary Figure 9) or height (Supplementary Figure 10) were not notably associated with any GWG phenotypes.

## Discussion

We have shown that approximately 20% of the variability in GWG can be explained by common maternal genetic variants. A much smaller contribution is made by common genetic variants from the fetal genome. This pattern of maternal and fetal genetic contribution is opposite to what we see with birth weight, for which the fetal contribution is greater. Despite this modest genetic contribution, which is similar to the common genetic contribution to birth weight and many other phenotypes,^28^ in what we believe to be the first GWAS of GWG, we were unable to identify any genetic variants that reached genome-wide levels of significance (*P*<5 × 10^−8^) and that replicated. Given the possible contribution of several adiposity-related phenotypes to overall GWG, we also investigated whether genetic variants that are known to be associated with these traits were also associated with GWG. Some maternal BMI, fasting glucose and type 2 diabetes variants were nominally associated with GWG, such that those that were associated with increased BMI, glucose or type 2 diabetes, were associated with lower early and higher late GWG. Some maternal variants associated with higher systolic blood pressure were also associated with lower late GWG. In general, fetal variants associated with these traits were largely unrelated to GWG. Of note, established maternal and fetal birth weight variants were for the most part not related to GWG. The one exception being rs900400, a variant previously shown to be strongly related to birth weight in a genome-wide study of fetal genotype,^29^ which in our study was inversely associated with late and total GWG in the case of the maternal genotype. This variant has also been recently shown to be inversely associated with leptin in genome-wide analyses,^30^ and thus the inverse association of this variant in the mother with GWG may reflect a positive association of maternal leptin with GWG.

Using a twin study, Andersson *et al.*^14^ show that the heritability of first pregnancy GWG is 43% we were able to show that approximately half of this could be explained by common genetic variants or variants they tag in the maternal genome. This is similar to the proportion of heritability explained in other common traits such as height and BMI.^28^ It is perhaps not surprising that our results suggest that the maternal genome has a greater contribution to GWG than the offspring genome. On average, approximately 55% of GWG is a result of increased maternal tissue, 15–20% is due to the placenta and amniotic fluid, and 20–25% is a result of fetal tissue.^19^ The maternal genome will contribute to tissue expansion in the mother, as well as to placental size, amniotic fluid and fetal growth, whereas, it is likely that the fetal genome will only contribute to placenta, amniotic fluid and fetal growth. We detected some evidence of a negative genetic covariance in the M-GCTA analysis of late GWG. A negative covariance implies that a proportion of maternal genetic variants associated with increased GWG are associated with decreased GWG when present in the fetal genome. Although this was a surprising result, it is not inconceivable. For example, there is a well-described relationship between mutations in the glucokinase gene (*GCK*) and offspring birth weight, whereby if the mutation is present in the mother and not the offspring then the birth weight is increased, whereas if the mutation is present in the offspring but not the mother then the birth weight is decreased.^31^ Given birth weight is a component of GWG, it is plausible that variants in *GCK* and other mutations involved in insulin secretion could produce similar effects on GWG. However, given the large standard error on the estimate, this negative covariance might be a chance finding and requires replication before any further interpretation is made.

Our lack of replicated genome-wide significant findings might be due to the complexity of the GWG phenotype. Weight was measured by trained personnel during pregnancy in the majority of studies included in this meta-analysis, however, the pre-pregnancy measure was often self-report and the early-pregnancy measure would have included some pregnancy weight gain. This would have increased the measurement error for GWG, making it difficult to identify true genetic associations. In addition, we had low statistical power to detect associations with genetic variants, which have a small effect. With an alpha of 5 × 10^−8^ in the maternal GWAS of total GWG, we had 80% power to detect a genetic variant that explained between 0.37 and 0.4% of the variance for our range of sample sizes (*N*=9832–10 543). Similarly, we had 80% power to detect a variant that explained 0.24–0.3% of the variance in the offspring GWAS of total GWG (*N*=12 995–16 317). However, for other complex quantitative phenotypes, such as BMI, the genetic variants discovered to date each explain 0.003–0.325% of the variance,^32^ indicating that many common genetic variants each of small effect influence the trait. Therefore, we had adequate power to detect common genetic variants with modest to large effects, but we were unable to detect variants with smaller effects, although we used the largest sample of individuals for exploring genetic associations with this phenotype to date and are unaware currently of other European origin studies that could have added to this effort.

Despite most of our analyses suggesting a stronger contribution of maternal, than fetal common genetic variants to GWG, the one nominally significant variant that replicated was for a fetal variant that was related to total GWG. This variant on chromosome 18, is near to the Synaptotagmin 4 (*SYT4*) gene, which is a protein-coding gene involved in calcium and syntaxin binding.^33^ Its relation to GWG is unclear and this association should be treated with caution unless further replicated, particularly as the association was only nominal and did not reach conventional GWAS significance of 5 × 10^−8^ even in combined meta-analysis of discovery and replication samples (the result being per allele difference in mean total GWG: 0.06 (95% CI: 0.04, 0.08) kg per week; *P*=1.6 × 10^−5^ in pooled discovery and replication).

There is little clear evidence linking most of the genes nearest our top 10 loci to GWG. However, some speculative evidence exists for the following nearest genes. The gene nearest our strongest association in the discovery GWAS was *PSG5*, which belongs to a group of molecules that are mainly produced during pregnancy by the placental syncytiotrophoblasts. Pregnancy-specific beta-1 glycoprotein (PSBG) levels have been correlated with placental function and fetal well-being.^34^ *TMEM163*, which harbors rs481396, is a putative zinc transporter.^35^ Zinc has been shown to be positively correlated with birth weight.^36^ Two of the genes nearest to the index SNPs taken forward for replication have previously been associated with adult height,^37^ *LCORL* and *HLA-C*. *LCORL* has also been associated with birth weight^29^ and birth length,^38^ which might be driving the association with GWG. *HLA-C*, being part of the major histocompatibility complex on chromosome 6 has also been associated with various auto-immune diseases such as psoriasis, Crohn’s disease and atopic-dermatitis. Variants in *TCERG1L* have been shown to be associated with fasting insulin and insulin resistance in an African–American population,^39^ which could have implications for growth of both the mother and the offspring.

We expanded on previous studies that examined the associations of candidate maternal and/or fetal adiposity or diabetes related genetic variants with GWG^15, 16, 17^ by looking at a wider variety of phenotypes that are observationally related to GWG (previous studies looked only at BMI and type 2 diabetes), in a considerably larger sample of participants and using a greater number of variants for each phenotype. We are aware that for some of the phenotypes investigated, there are a larger number of associated SNPs in the more recent GWAS, for example, over 90 variants have now been shown to independently relate to BMI.^32^ The subset of variants that we used for each phenotype were those with the largest effect sizes on each of the individual traits and that were available in the majority of replication (as well as discovery) samples, therefore we will have greater power to detect an effect with GWG if one exists.

The main strength of this study was the availability of both maternal and offspring genotype and the three separate phenotypes for GWG allowing us to investigate whether genetic variants had consistent effects throughout pregnancy. Our main analyses explored genetic contributions to total GWG. This enabled us to maximize our sample size, but it assumes that weight gain is linear across gestation. Evidence suggests that GWG is slower in early pregnancy up to 14–18 weeks depending on how it is measured and the population studied, but for the majority of pregnancy from this time point it is broadly linear.^1, 20^ Furthermore, we examined associations (in smaller subsets) with early and late GWG. The choice of 18–20 weeks to distinguish between early and late GWG was determined on the basis of maximizing sample sizes, evidence for differences in fetal and maternal contributions before and after this time,^1, 19^ and multilevel modeling in the ALSPAC cohort, which was the largest contributing cohort to our study with multiple repeated weight measures throughout pregnancy.^20^ The weak correlation between early and late GWG in our largest cohort contributing to these measures, supports these two periods being relatively independent of each other. GWG varies by pre- or early-pregnancy BMI and other characteristics such as parity and ethnicity,^2^ and some, but not all, studies suggest that associations of GWG with maternal and offspring outcomes varies by maternal BMI; the review that informed the Institute of Medicine recommendations for GWG noted that even for associations with outcomes that appeared to vary by BMI these subgroup analyses could not be considered robust.^2^ We were interested here in maternal and fetal genetic contributions to GWG in whole populations, and as with other studies of candidate genes with GWG, we have not looked at whether GWAS results would differ by maternal BMI. We would have limited power to do so and would be concerned about spurious subgroup findings from such analyses. We acknowledge that further analyses in studies that would enable more refined GWAS of trajectories of GWG across pregnancy, and with larger sample sizes that could explore possible interactions with maternal characteristics, such as BMI, ethnicity and parity, would be beneficial. However, our knowledge of the literature and potentially suitable studies suggests that currently only ALSPAC has detailed repeat measurements together with genome-wide data on mothers and offspring and it alone is too small to be adequately powered for such analyses. Furthermore, we have made every effort to engage with all cohorts that have both genetic and phenotypic data. Despite being the first large GWAS of this trait, to our knowledge and our effort to include all studies of European origin women with relevant data, we had limited power to detect variants with weak effects and will continue to seek additional studies to contribute to large GWAS in the future.

In summary, we have identified that a substantial proportion of the variation in GWG can be explained by common variants in the maternal genome, with an additional smaller proportion being explained by the offspring genome. In what we believe to be the first GWAS of GWG, using the largest collection of individuals, we were unable to identify any loci with a large effect on GWG and that replicated, but found some evidence that maternal variants may contribute more to GWG than fetal variants. These initial results suggest that the association of GWG with later offspring outcome may reflect intrauterine (maternal) effects. However, given the composite nature of GWG, including increasing maternal fat stores and plasma volume, the growing fetus, placenta and amniotic fluid, larger sample sizes are required to identify individual genetic loci for GWG.

## Figures and Tables

**Table 1:** The most significant SNPs from each locus that reached *P*<10^−5^ from the discovery meta-analysis in all individuals

	*Mat or Off*	*Chr*	*Position (bp)*	*EA/OA*	*EAF*^a^	*Early GWG*				*Late GWG*				*Total GWG*			
						N	*Beta*^b^	*s.e.*	P*-value*	N	*Beta*^b^	*s.e.*	P-*value*	N	*Beta*^b^	*s.e.*	P*-value*
*rs481396 (TMEM163)*																	
Discovery	Mat	2	134 953 875	T/C	0.72	7704	0.059	0.018	9.1 × 10^−4^	7681	0.062	0.018	4.1 × 10^−4^	10 537	0.068	0.015	6.8 × 10^−6^
Replication	Mat	2	134 953 875	T/C	0.68	1575	0.003	0.039	0.93	1637	**−0.077**	**0.038**	**0.04**	7883	**−**0.021	0.017	0.21
Discovery	Off	2	134 953 875	T/C	0.70	8552	0.034	0.017	0.05	8623	0.030	0.017	0.08	15 642	0.027	0.013	0.03
Replication	Off	2	134 953 875	T/C	0.70	3288	0.037	0.028	0.18	778	0.020	0.060	0.73	4546	0.014	0.025	0.57
																	
*rs3924699 (LCORL)*																	
Discovery	Mat	4	18 300 153	G/C	0.10	7704	0.101	0.028	3.6 × 10^−4^	7681	0.109	0.028	1.1 × 10^−4^	10 543	0.106	0.024	9.0 × 10^−6^
Replication	Mat	4	18 300 153	G/C	0.11	1570	0.041	0.059	0.49	1633	0.003	0.057	0.96	7321	0.031	0.027	0.25
Discovery	Off	4	18 300 153	G/C	0.10	8552	0.054	0.027	0.04	8624	0.074	0.027	5.5 × 10^−3^	15 636	0.042	0.021	0.05
Replication	Off	4	18 300 153	G/C	0.08	3280	0.131	0.076	0.08	771	**−**0.017	0.139	0.90	4380	0.023	0.041	0.58
																	
*rs9995522 (UGDH)*																	
Discovery	Mat	4	39 179 591	A/G	0.93	7704	0.118	0.033	4.1 × 10^−4^	7681	0.035	0.033	0.30	10 337	0.047	0.029	0.11
Rep lication	Mat	4	39 179 591	A/G	0.94	1303	0.153	0.091	0.09	1365	0.030	0.089	0.74	7074	**0.070**	**0.035**	**0.05**
Discovery	Off	4	39 179 591	A/G	0.93	8552	0.161	0.032	5.3 × 10^−7^	8625	0.051	0.032	0.12	16 317	0.094	0.024	8.3 × 10^−5^
Replication	Off	4	39 179 591	A/G	0.94	3284	**−**0.037	0.060	0.53	774	0.019	0.122	0.88	4492	0.020	0.040	0.62
																	
*rs6457375 (HLA-C)*																	
Discovery	Mat	6	31 380 591	G/A	0.52	7704	0.077	0.017	3.4 × 10^−6^	7681	0.035	0.017	0.04	9832	0.049	0.015	9.5 × 10^−4^
Replication	Mat	6	31 380 591	G/A	0.53	1294	0.002	0.036	0.96	1346	**−**0.035	0.036	0.33	6976	**−**0.001	0.015	0.96
Discovery	Off	6	31 380 591	G/A	0.52	8552	0.036	0.016	0.03	8625	0.038	0.016	0.02	15 166	0.029	0.012	0.02
Replication	Off	6	31 380 591	G/A	0.51	3209	**−**0.005	0.025	0.85	700	0.030	0.054	0.58	2972	**−**0.007	0.024	0.76
																	
*rs13295979 (ERCC6L2/HSD17B3)*																	
Discovery	Mat	9	97 879 618	T/G	0.90	7704	0.113	0.037	2.4 × 10^−3^	7681	0.120	0.037	1.3 × 10^−3^	9832	0.138	0.033	2.4 × 10^−5^
Replication	Mat	9	97 879 618	T/G	0.92	1126	0.047	0.082	0.57	1112	0.028	0.083	0.74	5656	**−**0.004	0.035	0.91
Discovery	Off	9	97 879 618	T/G	0.90	8552	0.082	0.035	0.02	8623	0.073	0.035	0.04	15 166	0.078	0.027	3.3 × 10^−3^
Replication	Off	9	97 879 618	T/G	0.92	3209	**−**0.031	0.046	0.50	700	**−**0.039	0.096	0.68	3599	0.063	0.045	0.16
																	
*rs1702200 (GLRX3/TCERG1L)*																	
Discovery	Mat	10	132 346 882	G/T	0.50	7704	0.035	0.017	0.03	7681	0.060	0.017	2.9 × 10^−4^	10 475	0.064	0.014	6.6 × 10^−6^
Replication	Mat	10	132 346 882	G/T	0.50	1576	**−**0.003	0.036	0.92	1638	0.025	0.035	0.48	7891	**−**0.024	0.016	0.14
Discovery	Off	10	132 346 882	G/T	0.50	8552	**−**0.011	0.016	0.50	8624	0.021	0.016	0.17	13 424	0.024	0.013	0.06
Replication	Off	10	132 346 882	G/T	0.50	3287	0.009	0.025	0.70	777	0.081	0.051	0.12	4527	0.014	0.022	0.54
																	
*rs7133083 (RBM19)*																	
Discovery	Mat	12	112 942 277	A/G	0.83	7704	0.108	0.022	1.5 × 10^−6^	7681	0.056	0.023	0.01	9832	0.090	0.020	6.4 × 10^−6^
Replication	Mat	12	112 942 277	A/G	0.82	1582	0.028	0.049	0.57	1644	0.053	0.048	0.27	7361	0.005	0.021	0.83
Discovery	Off	12	112 942 277	A/G	0.83	8552	0.042	0.021	0.05	8623	0.005	0.021	0.80	15 165	0.018	0.016	0.26
Replication	Off	12	112 942 277	A/G	0.84	3208	**−**0.006	0.039	0.88	699	**−**0.030	0.087	0.73	3637	0.040	0.033	0.23
																	
*rs7301563 (NTF3)*																	
Discovery	Mat	12	5 436 393	T/C	0.18	7704	0.024	0.022	0.26	7681	0.019	0.022	0.39	10 325	0.027	0.019	0.15
Replication	Mat	12	5 436 393	T/C	0.19	1576	0.042	0.047	0.38	1644	0.073	0.043	0.11	7355	**−**0.001	0.021	0.97
Discovery	Off	12	5 436 393	T/C	0.18	8552	0.088	0.019	4.9 × 10^−6^	8625	0.035	0.019	0.07	15 163	0.041	0.015	6.5 × 10^−3^
Replication	Off	12	5 436 393	T/C	0.18	3270	**−**0.060	0.032	0.06	760	**−**0.061	0.065	0.35	4312	**−**0.005	0.029	0.87
																	
*rs310087 (SYT4)*																	
Discovery	Mat	18	39 147 836	A/G	0.49	7704	0.013	0.017	0.42	7681	0.023	0.017	0.17	9832	0.016	0.015	0.26
Replication	Mat	18	39 147 836	A/G	0.47	1581	**−**0.038	0.035	0.28	1643	**−**0.002	0.035	0.95	7357	0.009	0.017	0.59
Discovery	Off	18	39 147 836	A/G	0.48	8552	0.041	0.016	7.8 × 10^−3^	8623	0.055	0.016	4.6 × 10^−4^	12 995	0.060	0.013	3.0 × 10^−6^
Replication	Off	18	39 147 836	A/G	0.50	3285	**−**0.004	0.025	0.87	775	**−**0.003	0.051	0.95	4452	**0.050**	**0.022**	**0.03**
																	
*rs16989175 (PSG5)*																	
Discovery	Mat	19	48 337 381	G/C	0.76	7704	0.068	0.020	5.3 × 10^−4^	7681	0.011	0.020	0.58	10 445	0.047	0.017	5.3 × 10^−3^
Replication	Mat	19	48 337 381	G/C	0.77	1577	**−**0.076	0.044	0.09	1639	**−**0.056	0.043	0.20	10 660	0.011	0.016	0.47
Discovery	Off	19	48 337 381	G/C	0.76	8552	0.046	0.019	0.02	8624	0.058	0.019	2.4 × 10^−3^	15 568	0.079	0.014	1.7 × 10^−8^
Replication	Off	19	48 337 381	G/C	0.76	3270	**−**0.040	0.028	0.15	760	**−**0.076	0.058	0.19	7561	**−**0.005	0.019	0.78

Abbreviations: EA/OA: effect allele/other allele; EAF, effect allele frequency; GWG, gestational weight gain; Mat, maternal genome; Off, Offspring (that is, fetal genome); SNP, single-nucleotide polymorphism.

The nearest gene is used as the locus name. Values in bold indicate replication *P*⩽0.05.

a Average EAF across the cohorts in the total GWG meta-analyses.

b Beta values are the difference in mean GWG in kg per week of gestation per additional effect allele.

**Table 2:** Estimates (s.e.) of proportion of maternal and fetal genetic contributions from common variants to gestational weight gain

	*GREML results*^a^		*M-GCTA results*		
	*Maternal genome* (N=*6435*)	*Child genome* (N=*6435*)	*Maternal genome* (N=*4078*)	*Child genome* (N=*4078*)	*Covariance* (N=*4078*)
Early	0.195 (0.055)	0.058 (0.053)	0.021 (0.113)	0.000 (0.115)	0.067 (0.091)
Late	0.244 (0.054)	0.110 (0.053)	0.196 (0.113)	0.161 (0.114)	−0.039 (0.091)
Total	0.239 (0.055)	0.121 (0.053)	0.173 (0.112)	0.045 (0.113)	0.016 (0.090)
Birth weight	0.13 (0.06)	0.18 (0.06)	0.04 (0.10)	0.24 (0.11)	0.04 (0.08)

Abbreviations: GREML, genetic restricted maximum likelihood; GWG, gestational weight gain; M-GCTA, maternal genome-wide complex trait analysis.

a *P*-values for the GREML results are: maternal genome, early GWG=1.12 × 10^−4^, maternal genome, late GWG=8.83 × 10^−7^, maternal genome, total GWG=1.94 × 10^−6^, maternal genome, birth weight=0.02, offspring genome, early GWG=0.130, offspring genome, late GWG=0.015, offspring genome, total GWG=0.008, offspring genome, birth weight=1.86 × 10^−3^.

**Table 3:** Results from the unconditional analysis and analysis conditional on offspring (row 2 of each SNP) or maternal genotype (row 4 of each SNP) for the most significant SNPs from each locus that reached *P*<10^−5^ from the discovery meta-analysis

	*Genome*	*Chr*	*Position (bp)*	*EA/OA*	*Early GWG*				*Late GWG*				*Total GWG*^a^			
					N	*Beta*^a^	*s.e.*	P*-value*	N	*Beta*^a^	*s.e.*	P*-value*	N	*Beta*	*s.e.*	P*-value*
*rs481396 (TMEM163)*																
Unconditional	Mat	2	134 953 875	T/C	6635	0.040	0.019	0.03	6674	0.057	0.019	2.3 × 10^−3^	12 844	0.036	0.013	7.0 × 10^−3^
Conditional	Mat	2	134 953 875	T/C	6103	0.041	0.022	0.06	6165	0.050	0.022	0.03	10 079	0.031	0.017	0.07
Unconditional	Off	2	134 953 875	T/C	6291	0.040	0.019	0.04	6356	0.031	0.019	0.11	11 340	0.033	0.015	0.03
Conditional	Off	2	134 953 875	T/C	6103	0.012	0.022	0.59	6165	0.011	0.023	0.64	10 079	0.019	0.018	0.28
																
*rs3924699 (LCORL)*																
Unconditional	Mat	4	18 300 153	G/C	6630	0.068	0.030	0.02	6670	0.075	0.030	0.01	12 830	0.055	0.021	7.6 × 10^−3^
Conditional	Mat	4	18 300 153	G/C	6091	0.052	0.035	0.14	6155	0.050	0.035	0.15	10 031	0.030	0.027	0.28
Unconditional	Off	4	18 300 153	G/C	6283	0.050	0.030	0.09	6350	0.074	0.030	0.01	11 241	0.047	0.023	0.05
Conditional	Off	4	18 300 153	G/C	6091	0.025	0.035	0.47	6155	0.045	0.035	0.20	10 031	0.027	0.028	0.33
																
*rs9995522 (UGDH)*																
Unconditional	Mat	4	39 179 591	A/G	6638	0.113	0.035	1.5 × 10^−3^	6677	**−**0.020	0.026	0.44	12 838	0.055	0.026	0.03
Conditional	Mat	4	39 179 591	A/G	6101	0.041	0.042	0.34	6163	0.039	0.043	0.36	10 057	0.037	0.033	0.26
Unconditional	Off	4	39 179 591	A/G	6288	0.179	0.035	4.8 × 10^−7^	6354	**−**0.007	0.036	0.84	11 289	0.070	0.026	6.4 × 10^−3^
Conditional	Off	4	39 179 591	A/G	6101	0.163	0.041	8.2 × 10^−5^	6163	**−**0.024	0.042	0.56	10 057	0.060	0.031	0.05
																
*rs6457375 (HLA-C)*																
Unconditional	Mat	6	31 380 591	G/A	5474	0.057	0.019	3.1 × 10^−3^	5446	0.009	0.020	0.64	9530	0.020	0.014	0.16
Conditional	Mat	6	31 380 591	G/A	5378	0.052	0.022	0.02	5366	0.014	0.022	0.52	8688	0.029	0.017	0.09
Unconditional	Off	6	31 380 591	G/A	5480	0.023	0.020	0.23	5458	0.020	0.007	0.74	9388	0.005	0.015	0.71
Conditional	Off	6	31 380 591	G/A	5378	**−**0.0005	0.022	0.98	5366	**−**0.002	0.023	0.93	8688	**−**0.011	0.017	0.53
																
*rs13295979 (ERCC6L2/HSD17B3)*																
Unconditional	Mat	9	97 879 618	T/G	5826	0.117	0.040	3.7 × 10^−3^	5805	0.118	0.041	3.9 × 10^−3^	11 087	0.061	0.027	0.03
Conditional	Mat	9	97 879 618	T/G	5577	0.086	0.046	0.06	5564	0.105	0.047	0.02	9367	0.055	0.034	0.10
Unconditional	Off	9	97 879 618	T/G	5668	0.095	0.041	0.02	5643	0.103	0.042	0.01	10 051	0.094	0.030	2.1 × 10^−3^
Conditional	Off	9	97 879 618	T/G	5577	0.051	0.046	0.26	5564	0.052	0.046	0.27	9367	0.061	0.035	0.08
																
*rs1702200 (GLRX3/TCERG1L)*																
Unconditional	Mat	10	132 346 882	G/T	6636	0.008	0.017	0.65	6675	0.044	0.017	0.01	12 840	0.027	0.012	0.03
Conditional	Mat	10	132 346 882	G/T	6105	0.003	0.021	0.90	6167	0.054	0.021	0.01	10 082	0.032	0.016	0.05
Unconditional	Off	10	132 346 882	G/T	6290	**−**0.011	0.018	0.54	6356	0.022	0.018	0.23	11 328	0.021	0.014	0.13
Conditional	Off	10	132 346 882	G/T	6105	0.001	0.021	0.97	6167	**−**0.019	0.021	0.37	10 082	**−**0.001	0.016	0.97
																
*rs7133083 (RBM19)*																
Unconditional	Mat	12	112 942 277	A/G	5834	0.099	0.025	5.3 × 10^−5^	5812	0.042	0.025	0.09	11 147	0.040	0.017	0.02
Conditional	Mat	12	112 942 277	A/G	5580	0.105	0.029	2.7 × 10^−4^	5566	0.036	0.029	0.21	9430	0.042	0.022	0.06
Unconditional	Off	12	112 942 277	A/G	5667	0.033	0.025	0.20	5642	0.026	0.025	0.31	10 089	0.038	0.019	0.05
Conditional	Off	12	112 942 277	A/G	5580	**−**0.011	0.029	0.70	5566	0.001	0.029	0.96	9430	0.022	0.022	0.32
																
*rs7301563 (NTF3)*																
Unconditional	Mat	12	5 436 393	T/C	6636	0.041	0.022	0.07	6681	0.023	0.022	0.30	12 841	0.021	0.016	0.20
Conditional	Mat	12	5 436 393	T/C	6084	**−**0.012	0.026	0.64	6146	0.013	0.027	0.63	9965	0.002	0.021	0.94
Unconditional	Off	12	5 436 393	T/C	6274	0.105	0.022	3.2 × 10^−6^	6340	0.028	0.023	0.22	11 100	0.064	0.018	3.3 × 10^−4^
Conditional	Off	12	5 436 393	T/C	6084	0.089	0.026	7.5 × 10^−4^	6146	0.044	0.027	0.10	9965	0.071	0.021	8.7 × 10^−4^
																
*rs310087 (SYT4)*																
Unconditional	Mat	18	39 147 836	A/G	6641	0.012	0.017	0.49	6680	0.020	0.017	0.24	12 848	0.016	0.012	0.18
Conditional	Mat	18	39 147 836	A/G	6104	**−**0.009	0.020	0.66	6166	**−**0.001	0.021	0.95	10 059	**−**0.014	0.016	0.39
Unconditional	Off	18	39 147 836	A/G	6288	0.035	0.017	0.04	6353	0.057	0.017	1.1 × 10^−3^	11 278	0.058	0.014	1.6 × 10^−5^
Conditional	Off	18	39 147 836	A/G	6104	0.041	0.020	0.04	6166	0.057	0.021	5.3 × 10^−3^	10 059	0.067	0.016	3.1 × 10^−5^
																
*rs16989175 (PSG5)*																
Unconditional	Mat	19	48 337 381	G/C	6637	0.039	0.020	0.06	6676	0.003	0.020	0.87	16 162	0.029	0.013	0.02
Conditional	Mat	19	48 337 381	G/C	6085	0.026	0.034	0.28	6147	**−**0.024	0.024	0.32	13 143	0.004	0.017	0.82
Unconditional	Off	19	48 337 381	G/C	6273	0.057	0.021	7.4 × 10^−3^	6339	0.049	0.021	0.02	14 363	0.058	0.014	3.7 × 10^−5^
Conditional	Off	19	48 337 381	G/C	6085	0.042	0.024	0.08	6147	0.050	0.025	0.04	13 143	0.055	0.017	9.6 × 10^−4^

Abbreviations: EA/OA, effect allele/other allele; GWG, gestational weight gain; Mat, maternal genome; Off, Offspring (that is, fetal genome; SNP, single-nucleotide polymorphism.

The nearest gene is used as the locus name.

Results from the maternal and offspring genotypes are presented.

a Beta values are the difference in mean GWG in kg per week of gestation per additional effect allele.

## References

[bib1] Rasmussen KM, Yaktine AL. Weight gain during pregnancy: reexamining the guidelines. Committee to reexamine IOM pregnancy weight guidelines: US Institute of Medicine and National Research Council 2009. 20669500

[bib2] Viswanthan M, Siega-Riz AM, Moos M-K, Deierlein A, Mumford S, Knaack J et al Outcomes of Maternal Weight Gain, Evidence Report/Technology Assessment No. 168. AHRQ Publication No08-E009 ed. Rockville, MD, USA: Agency for Healthcare Research and Quality 2008.

[bib3] Lawlor DA, Relton C, Sattar N, Nelson SM. Maternal adiposity–a determinant of perinatal and offspring outcomes? Nat Rev Endocrinol 2012; 8: 679–688. 23007319 10.1038/nrendo.2012.176

[bib4] Macdonald-Wallis C, Tilling K, Fraser A, Nelson SM, Lawlor DA. Gestational weight gain as a risk factor for hypertensive disorders of pregnancy. Am J Obstet Gynecol 2013; 209: 327 e1–17. 23711667 10.1016/j.ajog.2013.05.042 PMC3807791

[bib5] Fraser A, Tilling K, Macdonald-wallis C, Sattar N, Brion M-J, Benfield L et al. Association of maternal weight gain in pregnancy with offspring obesity and metabolic and vascular traits in childhood. Circulation 2010; 121: 2557–2564. 20516377 10.1161/CIRCULATIONAHA.109.906081 PMC3505019

[bib6] Lawlor DA, Lichtenstein P, Fraser A, Langstrom N. Does maternal weight gain in pregnancy have long-term effects on offspring adiposity? A sibling study in a prospective cohort of 146,894 men from 136,050 families. Am J Clin Nutr 2011; 94: 142–148. 21562086 10.3945/ajcn.110.009324 PMC3127508

[bib7] Mamun AA, O'Callaghan M, Callaway L, Williams G, Najman J, Lawlor DA. Associations of gestational weight gain with offspring body mass index and blood pressure at 21 years of age: evidence from a birth cohort study. Circulation 2009; 119: 1720–1727. 19307476 10.1161/CIRCULATIONAHA.108.813436

[bib8] Fraser A, Tilling K, Macdonald-Wallis C, Hughes R, Sattar N, Nelson SM et al. Associations of gestational weight gain with maternal body mass index, waist circumference, and blood pressure measured 16 y after pregnancy: the Avon Longitudinal Study of Parents and Children (ALSPAC). Am J Clin Nutr 2011; 93: 1285–1292. 21471282 10.3945/ajcn.110.008326 PMC3095501

[bib9] Lewis AJ, Galbally M, Gannon T, Symeonides C. Early life programming as a target for prevention of child and adolescent mental disorders. BMC Med 2014; 12: 33. 24559477 10.1186/1741-7015-12-33 PMC3932730

[bib10] Lawlor DA. The Society for Social Medicine John Pemberton Lecture 2011. Developmental overnutrition–an old hypothesis with new importance? Int J Epidemiol 2013; 42: 7–29. 23508404 10.1093/ije/dys209

[bib11] Davey Smith G, Hemani G. Mendelian randomization: genetic anchors for causal inference in epidemiological studies. Hum Mol Genet 2014; 23: R89–R98. 25064373 10.1093/hmg/ddu328 PMC4170722

[bib12] Lawlor DA, Harbord RM, Sterne JAC, Timpson NJ, Davey Smith G. Mendelian randomization: using genes as instruments for making causal inferences in epidemiology. Stat Med 2008; 27: 1133–1163. 17886233 10.1002/sim.3034

[bib13] Tyrrell J, Richmond RC, Palmer TM, Feenstra B, Rangarajan J, Metrustry S et al. Genetic evidence for causal relationships between maternal obesity-related traits and birth weight. JAMA 2016; 315: 1129–1140. 26978208 10.1001/jama.2016.1975 PMC4811305

[bib14] Andersson ES, Silventoinen K, Tynelius P, Nohr EA, Sorensen TI, Rasmussen F. Heritability of gestational weight gain–a Swedish register-based twin study. Twin Res Hum Genet 2015; 18: 410–418. 26111621 10.1017/thg.2015.38

[bib15] Lawlor DA, Fraser A, Macdonald-Wallis C, Nelson SM, Palmer TM, Davey Smith G et al. Maternal and offspring adiposity-related genetic variants and gestational weight gain. Am J Clin Nutr 2011; 94: 149–155. 21593506 10.3945/ajcn.110.010751 PMC3127507

[bib16] Stuebe AM, Lyon H, Herring AH, Ghosh J, Wise A, North KE et al. Obesity and diabetes genetic variants associated with gestational weight gain. Am J Obstet Gynecol 2010; 203: 283–217. 20816152 10.1016/j.ajog.2010.06.069 PMC3222335

[bib17] Dishy V, Gupta S, Landau R, Xie HG, Kim RB, Smiley RM et al. G-protein beta(3) subunit 825 C/T polymorphism is associated with weight gain during pregnancy. Pharmacogenetics 2003; 13: 241–242. 12668921 10.1097/00008571-200304000-00009

[bib18] Oken E, Rifas-Shiman SL, Field AE, Frazier AL, Gillman MW. Maternal gestational weight gain and offspring weight in adolescence. Obstet Gynecol 2008; 112: 999–1006. 18978098 10.1097/AOG.0b013e31818a5d50 PMC3001295

[bib19] Pitkin RM. Nutritional support in obstetrics and gynecology. Clin Obstet Gynecol 1976; 19: 489–513. 954246 10.1097/00003081-197609000-00002

[bib20] Macdonald-Wallis C, Lawlor DA, Palmer T, Tilling K. Multivariate multilevel spline models for parallel growth processes: application to weight and mean arterial pressure in pregnancy. Stati Med 2012; 31: 3147–3164. 10.1002/sim.5385 PMC3569877 22733701

[bib21] Sharp G, Lawlor DA, Richmond RC, Fraser A, Simpkin A, Suderman M et al. Maternal pre-pregnancy BMI and gestational weight gain, offspring DNA methylation and later offspring adiposity: findings from the Avon Longitudinal Study of Parents and Children. Int J Epidemiol 2015; 44: 1288–1304. 25855720 10.1093/ije/dyv042 PMC4588865

[bib22] Yang J, Benyamin B, McEvoy BP, Gordon S, Henders AK, Nyholt DR et al. Common SNPs explain a large proportion of the heritability for human height. Nat Genet 2010; 42: 565–569. 20562875 10.1038/ng.608 PMC3232052

[bib23] Eaves LJ, Pourcain BS, Davey Smith G, York TP, Evans DM. Resolving the effects of maternal and offspring genotype on dyadic outcomes in genome wide complex trait analysis (‘M-GCTA’). Behav Genet 2014; 44: 445–455. 25060210 10.1007/s10519-014-9666-6 PMC4174369

[bib24] Boyd A, Golding J, Macleod J, Lawlor DA, Fraser A, Henderson J et al. Cohort profile: the 'children of the 90s'–the index offspring of the Avon Longitudinal Study of Parents and Children. Int J Epidemiol 2013; 42: 111–127. 22507743 10.1093/ije/dys064 PMC3600618

[bib25] Fraser A, Macdonald-Wallis C, Tilling K, Boyd A, Golding J, Davey Smith G et al. Cohort profile: the Avon Longitudinal Study of Parents and Children: ALSPAC mothers cohort. Int J Epidemiol 2013; 42: 97–110. 22507742 10.1093/ije/dys066 PMC3600619

[bib26] Willer CJ, Li Y, Abecasis GR. METAL: fast and efficient meta-analysis of genomewide association scans. Bioinformatics (Oxford, England) 2010; 26: 2190–2191. 10.1093/bioinformatics/btq340 PMC2922887 20616382

[bib27] Ihaka R, Gentleman R. R: a language for data analysis and graphics. J Comput Graph Stat 1996; 5: 299–314.

[bib28] Yang J, Manolio TA, Pasquale LR, Boerwinkle E, Caporaso N, Cunningham JM et al. Genome partitioning of genetic variation for complex traits using common SNPs. Nat Genet 2011; 43: 519–525. 21552263 10.1038/ng.823 PMC4295936

[bib29] Horikoshi M, Yaghootkar H, Mook-Kanamori DO, Sovio U, Taal HR, Hennig BJ et al. New loci associated with birth weight identify genetic links between intrauterine growth and adult height and metabolism. Nat Genet 2013; 45: 76–82. 23202124 10.1038/ng.2477 PMC3605762

[bib30] Kilpelainen TO, Carli JF, Skowronski AA, Sun Q, Kriebel J, Feitosa MF et al. Genome-wide meta-analysis uncovers novel loci influencing circulating leptin levels. Nat Commun 2016; 7: 10494. 26833098 10.1038/ncomms10494 PMC4740377

[bib31] Hattersley AT, Beards F, Ballantyne E, Appleton M, Harvey R, Ellard S. Mutations in the glucokinase gene of the fetus result in reduced birth weight. Nat Genet 1998; 19: 268–270. 9662401 10.1038/953

[bib32] Locke AE, Kahali B, Berndt SI, Justice AE, Pers TH, Day FR et al. Genetic studies of body mass index yield new insights for obesity biology. Nature 2015; 518: 197–206. 25673413 10.1038/nature14177 PMC4382211

[bib33] Adolfsen B, Littleton JT. Genetic and molecular analysis of the synaptotagmin family. Cell Mol Life Scie 2001; 58: 393–402. 10.1007/PL00000865 PMC11337341 11315187

[bib34] Gordon YP, Grudzinskas JG, Jeffrey D, Chard T. Concentrations of pregnancy-specific beta 1-glycoprotein in maternal blood in normal pregnancy and in intrauterine growth retardation. Lancet 1977; 1: 331–333. 64859 10.1016/s0140-6736(77)91135-7

[bib35] Cuajungco MP, Basilio LC, Silva J, Hart T, Tringali J, Chen CC et al. Cellular zinc levels are modulated by TRPML1-TMEM163 interaction. Traffic (Copenhagen, Denmark) 2014; 15: 1247–1265. 10.1111/tra.12205 PMC4205267 25130899

[bib36] Neggers YH, Cutter GR, Acton RT, Alvarez JO, Bonner JL, Goldenberg RL et al. A positive association between maternal serum zinc concentration and birth weight. Am J Clin Nutr 1990; 51: 678–684. 2321574 10.1093/ajcn/51.4.678

[bib37] Wood AR, Esko T, Yang J, Vedantam S, Pers TH, Gustafsson S et al. Defining the role of common variation in the genomic and biological architecture of adult human height. Nat Genet 2014; 46: 1173–1186. 25282103 10.1038/ng.3097 PMC4250049

[bib38] van der Valk RJ, Kreiner-Moller E, Kooijman MN, Guxens M, Stergiakouli E, Saaf A et al. A novel common variant in DCST2 is associated with length in early life and height in adulthood. Hum Mol Genet 2015; 24: 1155–1168. 25281659 10.1093/hmg/ddu510 PMC4447786

[bib39] Chen G, Bentley A, Adeyemo A, Shriner D, Zhou J, Doumatey A et al. Genome-wide association study identifies novel loci association with fasting insulin and insulin resistance in African Americans. Hum Mol Genet 2012; 21: 4530–4536. 22791750 10.1093/hmg/dds282 PMC3459464

