# Supplementary material for: Maternal and fetal genetic contribution to gestational weight gain
Source: Int J Obes (Lond). 2017 Nov 21;42(4):775–84. doi: 10.1038/ijo.2017.248 (PMC5784805; doi:10.1038/ijo.2017.248)
Supplement: Supplementary Material [file ijo2017248x1.docx]

**SUPPLEMENTARY MATERIAL: Maternal and fetal genetic contribution to gestational weight gain**

| **Content** | **Page(s)** | **Description** |
| --- | --- | --- |
| eMethods | 2 |  |
| Contributing studies | 2-8 | Description of each study including population, ethics and governance, assessment of phenotype and assessment of genotype |
| eTable 1 | 9-10 | Summary description of each cohort |
| eTable 2 | 11-18 | SNPs used in weighted allele scores |
| eTable 3 | 19 | Full results from M-GCTA for 4,078 maternal-offspring pairs from ALSPAC |
| eFigure 1 | 20 | Q-Q plots of the results from the discovery genome-wide association study |
| eFigure 2 | 21 | Manhattan plots of the results from the discovery genome-wide association study |
| eFigure 3 | 22-24 | Summary of previously reported loci for BMI and their effect on GWG using maternal genotype (A-C) and offspring genotype (D-F). |
| eFigure 4 | 25-27 | Summary of previously reported loci for glucose and their effect on GWG using maternal genotype (A-C) and offspring genotype (D-F). |
| eFigure 5 | 28-30 | Summary of previously reported loci for type 2 diabetes and their effect on GWG using maternal genotype (A-C) and offspring genotype (D-F). |
| eFigure 6 | 31-33 | Summary of previously reported loci for birth weight and their effect on GWG using maternal genotype (A-C) and offspring genotype (D-F). |
| eFigure 7 | 34-36 | Summary of previously reported loci for blood pressure and their effect on GWG using maternal genotype (A-C) and offspring genotype (D-F). |
| eFigure 8 | 37-39 | Summary of previously reported loci for waist-hip ratio and their effect on GWG using maternal genotype (A-C) and offspring genotype (D-F). |
| eFigure 9 | 40 | Summary of previously reported loci for vitamin D (25(OH)D) and their effect on GWG using maternal genotype and offspring genotype. |
| eFigure 10 | 41-43 | Summary of previously reported loci for height and their effect on GWG using maternal genotype (A-C) and offspring genotype (D-F). |
| Funding individual studies and authors | 44-46 |  |
| Acknowledgements individual studies | 47-49 |  |
| Full list of EGG consortium members | 50-55 |  |
| eReferences | 56-57 | Reference list for supplementary material |

**eMethods**

***Individual study descriptions***

**The Avon Longitudinal Study of Parents and Children (ALSPAC)** is a prospective population-based birth cohort study that recruited 14,541 pregnant women resident in Avon, UK with expected dates of delivery from 1^st^ April 1991 to 31^st^ December 1992 (<http://www.alspac.bris.ac.uk>.).[^1^](#_ENREF_1)^,^[^2^](#_ENREF_2) ***Ethical approval*** was obtained from the ALSPAC Law and Ethics committee and relevant local ethics committees and all women provided informed written consent. ***GWG*** was determined using data abstracted from obstetric medical records by trained research midwives. Repeat data entry checks demonstrated error rates consistently < 1%. GWG was determined using data abstracted from obstetric medical records by trained research midwives.[^3^](#_ENREF_3) Every measurement of weight entered into the medical records (weight was measured at all antenatal clinic visits in the UK at the time of data collection for this study) and the corresponding gestational age and date at the time of the weight measurement were extracted (median (IQR) of weight measurements per woman: 12 (9, 13)). Multilevel models were used to determine individual trajectories of GWG and from these total, early and late GWG were calculated for each woman.[^3^](#_ENREF_3) Pre-pregnancy weight was based on woman-report in early pregnancy and correlated highly with predicted weight at conception from the multilevel models (Pearsons’ correlation = 0.96). Birthweight, gestational age (in completed weeks) and fetal sex were obtained from obstetric/perinatal records. ***Maternal*** ***genome-wide*** data were obtained from the genome-wide Illumina 610 Quad Array. ***Fetal*** ***genome-wide*** data were obtained from the genome-wide Illumina 550 Quad Array. Genotype data were imputed against Hapmap phase2 build 36 release 22 using MACH after quality control (MAF>1%, HWE>1x10^-7^, call rate per SNP and person >95%). ALSPAC contributed to maternal and fetal discovery GWAS for total, early and late GWG.

Please note that the study website contains details of all the data that is available through a fully searchable data dictionary:

http://www.bris.ac.uk/alspac/researchers/data-access/data-dictionary/

**The Danish National Birth Cohort-Genomics of extremely Overweight Young Adults (DNBC-GOYA)** is a sub-study of the Danish National Birth Cohort (DNBC).[^4^](#_ENREF_4) The DNBC is a collection of data on 92,274 pregnant women recruited between 1996 and 2002, from their first antenatal visit to their general practitioner (primary care physician). Of 67,853 women who had given birth to a live born infant and provided a blood sample during pregnancy and had BMI information available, 3.6% with the largest residuals from the regression of BMI on age and parity (all entered as continuous variables) were selected as DNBC-GOYA extremely overweight cases. The BMI for these 2451 women ranged from 32.6 to 64.4kg/m^2^. From the remaining cohort a random sample of similar size (2450) was selected as controls. In total, 3908 of these women were successfully genotyped. The DNBC-GOYA offspring sample consists of 1000 offspring equally distributed between cases and controls. ***Ethical approval*** was obtained from the regional scientific ethics committee and the Danish Data Protection Board. ***GWG*** was determined during pregnancy, women provided information twice about their weight (and their gestational age at the time of this report) in mailed questionnaires; these data were used to compute early and late GWG when the timing of the maternal report allowed this. At 6-months post-partum all women reported their total GWG in a telephone interview and this together with their gestational age at delivery (obtained from medical records) was used for total GWG. ***Maternal genome-wide*** data were obtained from the genome-wide Illumina Human 610-Quad v1.0 BeadChip (545,349 SNPs). Of the initial 4901 women, 1960 extremely overweight cases and 1,948 control women were genotyped and passed quality control. Data were imputed to HapMap release 22, as previously described.[^4^](#_ENREF_4) ***Fetal replication* *genotyping*** were obtained from Illumina Human CoreExom v1.0. After QC, 941 individuals and 524,046 SNPs remained. Data were imputed to HapMap release 22, as previously described.[^4^](#_ENREF_4) GWAS on fetal samples only became available recently and after we had completed discovery analyses therefore relevant SNPs were selected from the fetal GWAS data and it was used as a replication sample. Information on total DNBC-GOYA contributed to maternal discovery GWAS and fetal replication analyses for total, early and late GWG.

**The Danish National Birth Cohort-Preterm Birth Study (DNBC-PTB)** is a case-control study using mother-infant pairs to investigate genetic and environmental influences on spontaneous preterm birth. The study is nested within the Danish National Birth Cohort;[^5^](#_ENREF_5) for case pairs delivery occurred before 37 weeks of gestation and for control pairs delivery occurred in gestational week 39, 40, or 41. Exclusion criteria were multiple deliveries, pregnancy complications such as placental abnormalities, preeclampsia/eclampsia, congenital abnormalities or stillbirth. Individuals were further required to be of Northern European ancestry. ***Ethical approval*** was obtained from the Regional Scientific Ethical Committee of Copenhagen and the study was also approved by the Danish Data Protection Agency. ***GWG*** was calculated as described for the GOYA cohort above. Outliers more than 4 SDs from the mean of GWG distributions were removed. ***Maternal and fetal genome-wide genotype*** data were obtained using the Illumina Human 660W Quad array and generated as part of the Gene Environment Association Studies (GENEVA) consortium. Data cleaning and QC steps were based on the following requirements for samples and SNPs to be included: sample missingness rate < 5%, variant missingness rate < 2%, MAF cases + MAF controls > 1%, A/T and C/G variants excluded. Imputation was done separately for mothers and infants using the MACH software package against the HapMap phase2 build 36 release 22. Only control mothers and infants were used for the GWG analyses and women overlapping between the GOYA and the DNBC-PTB were excluded from analysis of the maternal genotype data. In total 918 mother-infant pairs contributed to the GWG analyses. DNBC contributed to maternal and fetal discovery GWAS for total, early and late GWG.

**The Exeter Family Study Of Child Health (EFSOCH)** is a prospective study of children born between 2000 and 2004, and their parents, from a geographically defined region of Exeter, UK.[^6^](#_ENREF_6) ***Ethical approval*** was from the local research ethics committees and all adult participants gave informed written consent. Anthropometric measurements for calculation of ***GWG*** were taken from the mothers during the 28^th^ week of pregnancy. Weight was measured to the nearest 100g using Tanita digital electric scales (model number THD-305). Three measurements were made and a mean value calculated. Pre-pregnancy weight was self-reported and total GWG calculated as the weight at 28 weeks gestation minus pre-pregnancy weight. ***Maternal and fetal replication genotyping*** of selected SNPs was performed by LGC Genomics (formerly KBioscience; [www.lgcgroup.com)](http://www.lgcgroup.com)) using KASPar chemistry, which is a competitive allele-specific PCR SNP genotyping system. The median genotyping call rate was 0.95 and there was no evidence of deviation from Hardy-Weinberg Equilibrium in either parents or children (all Bonferroni-corrected *P* values >0.05). Concordance between duplicate genotyped samples was >0.99. EFSOCH contributed to maternal and fetal replication for total GWG only.

**The Genetics of Glucose regulation in Gestation and Growth (Gen3G)** is a prospective population-based pre-birth cohort that recruited 1034 pregnant women receiving prenatal care at the Centre Hospitalier Universitaire de Sherbrooke (CHUS) in QC, Canada between January 2010 to June 2013.[^7^](#_ENREF_7) ***Ethical approval*** was obtained from the CHUS ethics committee board and every participant gave written informed consent before enrolment in the study. Total ***GWG*** was determined using late (>36 weeks) pregnancy weight from electronic medical records minus self-reported pre-gestational weight. ***Maternal replication genotyping*** data were obtained from the MegaEx BeadArray of Illumina by Genome Quebec Innovation Centre. Directly genotyped SNPs or proxies with r^2^>0.8 present in the replication list were included in the analysis, after standard QCs (HWE>0.01, call rate per SNP >95%, call rate per person >98%). Gen3G contributed to maternal replication for total GWG.

The **Generation R Study** **(Gen R)** is a population-based prospective birth cohort. All children were born between April 2002 and January 2006 and were from the Rotterdam area of the Netherlands (<http://www.generationr.nl/>).[^8^](#_ENREF_8) ***Ethical approval*** was obtained from Medical Ethics Committee of the Erasmus MC, University Medical Center Rotterdam and written informed consent was obtained for each participant. ***GWG*** was determined using maternal weight (kg) measured in early-, mid- and late-pregnancy without shoes and heavy clothing. Information about maternal pre-pregnancy weight was obtained by questionnaire at enrolment in the study. Using these measurements, we calculated total, early and late gestational weight gain.  ***Fetal*** ***genome-wide*** data were obtained from the genome-wide Illumina 610 Quad and 660 platforms. Genotype data were imputed against Hapmap phase2 build 36 release 22 cosmopolitan panel using MACH version 1.0.15 after quality control (MAF>0.1%, HWE>1x10^-6^, SNP call rate >98%, sample call rate > 97.5%). ***Maternal replication genotyping*** was undertaken at LGC Genomics (formerly KBioscience; [www.lgcgroup.com)](http://www.lgcgroup.com)) using KASPar chemistry, which is a competitive allele-specific PCR SNP genotyping system. Gen R contributed to the fetal discovery GWAS and the maternal replication for total, early and late GWG.

**The German Infant study on the influence of Nutrition Intervention PLUS environmental and genetic influences on allergy development (GINIplus)** is a prospective population based birth cohort study.[^9^](#_ENREF_9) A total of 5991 mothers and their newborns were recruited between September 1995 and June 1998 in Munich and Wesel. Infants with at least one allergic parent and/or sibling were allocated to the interventional study arm investigating the effect of different hydrolysed formulas for allergy prevention in the first year of life. All children without a family history of allergic diseases and children whose parents did not give consent for the intervention were allocated to the non-interventional arm. In the present analysis, only children from the Munich study center were included. DNA was collected at the age 6 and 10 years. ***Ethical approval*** by the local Ethics Committees and written consent from participant’s families were obtained. ***GWG*** was calculated using information on maternal weight, measured at the beginning and at the end of pregnancy, which was collected retrospectively at age 15 years. If possible, this information was obtained from a maternity certificate routinely completed in obstetrical practices in Germany. ***Fetal genome-wide*** data were obtained from the Affymetrix Human SNP Array 5.0. Genotype data were imputed against 1000 Genomes Project Phase I v3 as reference (March 2012 release, updated version from 26 Aug 2012, all ancestries, limited to variants with more than one minor allele copy) using IMPUTE2 after quality control (MAF>1%, HWE>1x10-5, call rate per SNP and person >95% ). GINIplus contributed to total GWG.

**The Hyperglycaemia and Adverse Pregnancy Outcome (HAPO) study** is a multi-centre study that was designed to clarify the risk of adverse pregnancy outcome associated with degrees of glucose intolerance in pregnancy that are less severe than overt diabetes.[^10^](#_ENREF_10) The HAPO participants included in this analysis were of European ancestry. ***Ethical approval*** of the protocol was approved by the Institutional Review Board in all field centres. All participants provided written informed consent. ***GWG*** was determined from weight in pregnancy which was measured at as close to 28 weeks as possible (median 28 weeks; inter-quartile range 27-29 weeks) by trained study personnel. Outer garments and shoes were removed, and weight was measured twice to the nearest 0.1 kg on a scale calibrated each day. A third weight was taken if the results of the first two measurements different by >0.5 kg. If a third measurement was taken, the average of the two nearest measurements was used. Pre-pregnancy weight was self-reported. ***Maternal and fetal replication genotyping*** of rs16989175 was performed by LGC Genomics (formerly KBioscience; [www.lgcgroup.com)](http://www.lgcgroup.com)) using KASPar chemistry, which is a competitive allele-specific PCR SNP genotyping system. The genotyping call rate was >0.97 and there was 100% concordance between duplicated control samples. There was no evidence of deviation from Hardy-Weinberg Equilibrium in either the total set of mothers or children included in the analysis (*P* >0.05). HAPO contributed to maternal and fetal replication analyses for total GWG only. All analyses were adjusted for centre ID.

**The Hormone Inflammation and Pregnancy (HIP)** study is a prospective cohort study of very severely obese (BMI>40kg/m^2^) and lean (BMI<25kg/m2) pregnant women in Edinburgh, Scotland recruited 2008-2013. ***Ethical approval*** was obtained from the local research ethics committee (reference: 08/S1101/39) and all participants gave written informed consent. ***GWG*** was calculated from weight measurements recorded by research midwives at the study visits including antenatal booking (~12 weeks), 28 and 36 weeks’ gestation and from these total, early and late GWG were calculated. ***Maternal replication genotyping*** were obtained using the OpenArray genotyping platform at the Wellcome Trust Clinical Research Facility Genetics Core Laboratory, Edinburgh. HIP contributed to maternal replication analyses for total, early and late GWG.

**The INfancia y Medio Ambiente (INMA)** study is a prospective population-based birth cohort (<http://proyectoinma.org/en_index.html>).[^11^](#_ENREF_11) Women from four different regions in Spain were recruited in early pregnancy between November 2003 and February 2008. The present study includes mothers and/or children from Gipuzkoa, Sabadell, and Valencia subcohorts. ***Ethical approval*** was obtained from the Ethical Committees of the centres involved in the study and participants provided written informed consent. ***GWG*** was determined using maternal self-reported pre-pregnancy weight obtained from questionnaires at recruitment, and maternal weights throughout pregnancy extracted from obstetric medical records (mean (sd) of measurements per woman: 6.5 (2.7)). The date of pregnancy beginning was based on self-reported last menstrual period (LMP) at recruitment. An early crown-rump length was used to correct LMP dating when gestational age based on self-reported LMP and ultrasound differed by 7 days or more (12.9% of  newborns). ***Fetal genome-wide*** data were obtained from the genome-wide Illumina HumanOmni1-Quad Beadchip. Genotype data were imputed against HapMap phase2 build 36 release 24 using IMPUTE after quality control (MAF>1%, HWE>1x10^-6^, call rate per SNP>95% and call rate per person >98%). ***Maternal replication genotyping*** was performed by LGC Genomics (formerly KBioscience; [www.lgcgroup.com)](http://www.lgcgroup.com)) using KASPar chemistry, which is a competitive allele-specific PCR SNP genotyping system. INMA contributed fetal discovery GWAS and maternal replication analyses for total, early and late GWG.

**The influence of Life-style factors on the development of the Immune System and Allergies in East and West Germany PLUS the influence of traffic emmissions and genetics (LISAplus) Study** is a prospective population based birth cohort study.[^12^](#_ENREF_12) A total of 3094 healthy, full-term neonates were recruited between 1997 and 1999 in Munich, Leipzig, Wesel and Bad Honnef. The participants were not pre-selected based on family history of allergic diseases. In the present analysis, only children from the Munich study centre were included. DNA was collected at the age of 6 and 10 years. ***Ethical approval*** by the local Ethics Committees and written informed consent from participant’s families was obtained. ***GWG*** was calculated using information on maternal weight at the beginning and end of pregnancy collected at birth. ***Fetal genome-wide*** data were obtained from the Affymetrix Human SNP Array 5.0. Genotype data were imputed against HapMap phase 2 build 36 release 22 using IMPUTE after quality control (MAF>1%, HWE>1x10^-6^, call rate per SNP and person >98%). LISAplus contributed the fetal discovery GWAS for total GWG.

**The Norwegian Mother and Child Cohort Study (MoBa)** is a prospective population-based pregnancy cohort conducted by the Norwegian Institute of Public Health.[^13^](#_ENREF_13)^,^[^14^](#_ENREF_14) Participants were recruited from all over Norway from 1999-2008. The women consented to participation in 40.6% of the pregnancies. The cohort now includes 114,500 children, 95,200 mothers and 75,200 fathers (<http://www.fhi.no/moba-en>). ***Ethical approval*** for the MoBa study was obtained by the Regional Committee for Medical Research Ethics and all women provided informed written consent. ***GWG*** analysis and results were calculated using data obtained from Norwegian Medical Birth Register and self-reported questionnaires[^15^](#_ENREF_15)^,^[^16^](#_ENREF_16). ***Maternal and fetal genome-wide*** data were obtained using Illumina Human660W-Quad genotyping BeadChip. Imputation was performed with reference panel HapMap phase2 build 36 release 22 using Plink 1.07 with pre-imputation quality control (MAF>1%, HWE>1x10^-6^, call rate per SNP >95% and person >97%). MoBa contributed to maternal and fetal discovery GWAS for total GWG.

**The North Finish Birth Cohort 1966 (NFBC1966)** is a prospective population-based birth cohort, including all the mothers (N = 12055) with children whose expected date of birth was in the year 1966 in the two northernmost provinces of Finland. In total, 12231 children were born into the cohort, of which 12058 were live-born. ***Ethical approval*** was obtained from the ethical committee of the Northern Ostrobothnia Hospital District and all participants gave written informed consent. ***GWG*** data were acquired from maternity clinic visits. ***Fetal genome-wide*** data were obtained from Illumina HumanCNV‐370DUO Analysis BeadChip array. Genotype data were imputed against HapMap Phase 2 build 36 release 21/22 using Impute2 after quality control (MAF >= 0.01, HWE p-value >= 5.7e-7, individual call rate >= 0.95, call rate per SNP >= 0.95 (or >=0.99 for SNPs with MAF < 0.05)). NFBC1966 contributed to fetal discovery GWAS for total GWG.

**The North Finish Birth Cohort 1986 (NFBC1986)** is a prospective population-based birth cohort. It includes all the mothers (N = 9362) with children (N = 9479, of which 9432 were live-born) whose expected date of birth was between July 1985 and June 1986 in the two northernmost provinces of Finland. ***Ethical approval*** was obtained from the ethical committee of the Northern Ostrobothnia Hospital District and all participants gave written informed consent. ***GWG*** data were acquired from maternity clinic visits. ***Fetal genome-wide*** data were obtained from the Illumina HumanOmniExpressExome-8v1.2 array. Genotype data were imputed against 1000Genomes phase 3 release August 2015 using Impute2 after quality control (HWE p-value >= 0.0001, call rate per SNP >= 0.99, individual call rate >= 0.95). NFBC1986 contributed to fetal replication analyses for total, early and late GWG.

**The Physical Activity and Nutrition in Children (PANIC)** study is a controlled physical activity and dietary intervention study in a population sample of 506 Finnish children aged 6-8 years at baseline in 2007-2009. ***Ethical approval*** was obtained from the Research Ethics Committee of the Hospital District of Northern Savo. All children and their parents gave their written informed consent. ***GWG*** data were obtained from the medical records of Kuopio University Hospital. ***Fetal genome-wide*** data were genotyped using Illumina CardioMetabochip. SNPs with HWE p-value >= 10^-6^, individual call rate >=95% and minor allele frequency > 1% were included. PANIC contributed to fetal discovery GWAS for total GWG.

**The Western Australian Pregnancy (Raine)** is a prospective population-based birth cohort (<http://www.rainestudy.org.au/>).[^17^](#_ENREF_17) Women were recruited in early pregnancy from the King Edwards Memorial Hospital in Perth, Australia, between 1989 and 1991. ***Ethical approval*** was obtained from the Hospital Research Ethics Committee and women provided written informed consent. These women were required to estimate their pre-pregnancy weight, and had obstetric reviews at approximately 18 and 34weeks of gestation where multiple phenotypes (including weight) were recorded. ***GWG*** was determined using woman-reported pre-pregnancy weight and data abstracted from obstetric medical records by trained research midwives. ***Fetal*** ***genome-wide*** data were obtained from the Illumina 660 Quad Array. Genotype data were imputed against Hapmap phase2 build 36 release 22 using MACH after quality control (MAF>1%, HWE>5.7x10^-7^, call rate per SNP and person >95%). ***Maternal replication genotyping*** was carried out by LGC Genomics (formerly KBioscience; [www.lgcgroup.com)](http://www.lgcgroup.com)) using KASPar chemistry, which is a competitive allele-specific PCR SNP genotyping system. Raine contributed to fetal discovery GWAS and maternal replication analyses for total, early and late GWG.

The **Rhea** study examined a population-based sample of pregnant women and their children at the prefecture of Heraklion, Crete, Greece. Female residents (Greek and immigrants) who became pregnant during a period of one year starting in February 2007 were contacted and asked to participate in the study.[^18^](#_ENREF_18) The first contact was made at the time of the first major ultrasound examination (mean ± SD 11.96 ± 1.49 weeks of gestation) and several contacts followed (6th month of pregnancy, at birth, 6 months, 1st year and 4 & 6 years after birth). To be eligible for inclusion in the study, women had to have a good understanding of the Greek language and be older than 16 years of age. ***Ethical approval*** was obtained from the ethical committee of the University Hospital in Heraklion, Crete, Greece, and all participants provided written, informed consent after complete description of the study. ***GWG*** was determined using self-reported pre-pregnancy weight and measured mother’s height and weight, in light clothing and without shoes, were assessed in clinic at enrolment and in consecutive visits during pregnancy. ***Maternal and fetal replication genotyping*** was performed by LGC Genomics (formerly KBioscience; [www.lgcgroup.com)](http://www.lgcgroup.com)) using KASPar chemistry, which is a competitive allele-specific PCR SNP genotyping system. Rhea contributed fetal and maternal replication analyses for total, early and late GWG

**The Special Turku Coronary Risk Factor Intervention Project (STRIP)** is a prospective randomised life-style intervention trial that began in infancy and continued through childhood and adolescence to early adulthood (http://stripstudy.utu.fi/english.html). Altogether 1,062 children born in 1989-1991 were recruited at the age of 5 months by the well-baby clinics in Turku, and were randomised into an intervention group (n=540) or a control group (n=522). In this GWAS the study is treated as a cohort study with analyses conducted with data from participants in both arms and adjustment for randomised group. ***Ethical approval*** was obtained from the Joint Commission on Ethics of the Turku University and the Turku University Central Hospital. Informed consent was obtained from all parents at the beginning of the trial and from the children at 15 years of age. ***Total GWG*** was calculated as the difference between the highest measured weight during pregnancy and the weight before pregnancy. Both weight measures were obtained from maternity clinic records; weight before pregnancy was reported to clinicians by the woman. ***Fetal genome-wide*** data were directly genotyped with Illumina CardioMetabochip. SNPs with HWE p <10^-6^, call rate <95% and MAF<1% were excluded. STRIP contributed fetal discovery GWAS for total GWG.

**The Southampton Women’s Survey (SWS)** is a population-based prospective birth cohort study that recruited over 12,583 women aged 20-34 living in Southampton between 1998 and 2002 (http://www.mrc.soton.ac.uk/sws/). There were 3,158 babies born to women in the study between 1998 and 2007. ***Ethical approval*** was obtained from the Southampton and South West Hampshire Research Ethics Committee and all women provided written informed consent. ***GWG*** was determined using measurements made by research nurses before and during pregnancy. Total GWG was calculated from maternal weight before conception and maternal weight at an interview in late pregnancy, approximately 34 weeks gestation. Gestational age was estimated by LMP if available and accurate, otherwise by ultrasound scan. ***Maternal and fetal*** ***replication genotyping*** was performed by a U.K. company using an in-house calling algorithm. 5% duplicates were included with an error rate of 0%, call rate was >=96%. SWS contributed maternal and fetal replication analyses for total GWG.

**The TEENs of Attica: Genes and Environment (TEENAGE)** study is a cross-sectional study comprised of 707 (55.9% females) adolescents students aged 13–15 years attending the first three classes of public secondary schools located in the wider Athens area of Attica. ***Ethical approval***: Parents/guardians of students gave written consent, while participating students provided their verbal assent. The study was approved by the Ethics Committee of Harokopio University of Athens and the Greek Ministry of Education, Lifelong Learning and Religious Affairs. ***GWG*** information was collected retrospectively through phone interviews of participants’ mothers. ***Fetal genome-wide data*** were genotyped using the Illumina HumanOmniExpress BeadChip (Illumina, San Diego, CA, USA) at the Wellcome Trust Sanger Institute, Hinxton, UK. Genotype calling algorithm used was Illuminus (Teo et

al., 2007). Genotypes were imputed to HapMap phase2 build 36 release 22 using IMPUTE after quality control (HWE exact *p* < 0.0001, MAF < 1%, call rate < 95% for SNPs with MAF ≥ 5% or call rate < 99% for SNPs with MAF < 5%.). TEENAGE contributed fetal discovery GWAS for total GWG.

**eTable 1: Summary description of each contributing cohort**

| **Study** | **Ancestry group** | **Country of origin** | **Year(s) of pregnancy** | **Sample size by contribution*** | | | | **Phenotype source** | **Median (IQR) GWG (kg/week)** |
| --- | --- | --- | --- | --- | --- | --- | --- | --- | --- |
|  |  |  |  | **Mums discovery** | **Mums replication** | **Fetal discovery** | **Fetal replication** |  |  |
| ALSPAC | European | UK | 1990-1993 | Total: 7,192  Early: 7,192  Late: 7,192 |  | Total:7,192  Early: 7,192  Late: 7,192 |  | Extracted from hospital records | Total: 0.45 (0.35, 0.57)  Early: 0.30 (0.21, 0.41)  Late: 0.54 (0.42, 0.64) |
| DNBC-GOYA** | European | Denmark |  | Total: 1,757  Early: 360  Late: 344 |  |  | Total: 909  Early: 80  Late: 79 | Self-reported | Total: 0.36 (0.29, 0.45)  Early: 0.21 (0.11, 0.30)  Late: 0.52 (0.39, 0.68) |
| DNBC-PTB | European | Denmark | 1996-2003 | Total: 883  Early: 152  Late: 145 |  | Total: 903  Early: 152  Late: 146 |  | Self-reported | Total: 0.35 (0.27, 0.44)  Early: 0.17 (0.10, 0.26)  Late: 0.55 (0.40, 0.67) |
| EFSOCH | European | UK | 2000-2004 |  | Total: 799  Early: NA  Late: NA |  | Total: 694  Early: NA  Late: NA | Trained study midwives | Total: 0.35 (0.27, 0.45)  Early: NA  Late: NA |
| Gen3G | European | Canada | 2010-2014 |  | Total: 538  Early: NA  Late: NA |  |  | Electronic medical records | Total: 0.38 (0.30, 0.46)  Early: NA  Late: NA |
| Gen R | European | Netherlands | 2002-2006 |  | Total: 1,645  Early: 448  Late: 525 | Total: 1,992  Early: 544  Late: 635 |  | Hospital records and community midwives | Total: 0.36 (0.26, 0.44)  Early: 0.25 (0.16, 0.36)  Late: 0.53 (0.38, 0.68 |
| GINIplus | European | Germany | 1995-1998 |  |  |  | Total: 641  Early: NA  Late: NA | Extracted from hospital records | Total: 0.33 (0.26, 0.39)  Early: NA  Late: NA |
| HAPO | European | International | 2000-2006 |  | Total: 2,569  Early: NA  Late: NA |  | Total: 2,569  Early: NA  Late: NA | Trained study personnel | Total: 0.35 (0.25, 0.46)  Early: NA  Late: NA |
| HIP** | European | UK | 2008-2013 |  | Total: 275  Early: 275  Late: 275 |  |  | Trained study personnel | Total: 0.25 (0.13, 0.35)  Early: 0.08 (-0.02,0.17)  Late: 0.40 (0.22, 0.56) |
| INMA | European | Spain | 1997-2006 |  | Total: 1,549  Early: 357  Late: 353 | Total: 668  Early: 191  Late: 189 |  | Well-trained midwives and nurses | Total: 0.33 (0.26, 0.41)  Early: 0.26 (0.16, 0.36)  Late: 0.42 (0.31, 0.54) |
| LISAplus | European | Germany | 1997-1999 |  |  | Total: 544  Early: NA  Late: NA |  | Extracted from hospital records | Total: 0.35 (0.28, 0.41)  Early: NA  Late: NA |
| MoBa | European | Norway |  | Total: 723  Early: NA  Late: NA |  | Total: 486  Early: NA  Late: NA |  | Norwegian Medical Birth Register | Total: 0.36 (0.29, 0.46)  Early: NA  Late: NA |
| NFBC-1966 | European | Finland | 1965-1966 |  |  | Total: 2,173  Early: NA  Late: NA |  | Extracted from hospital records | Total: 0.28 (0.20-0.36)  Early: NA  Late: NA |
| NFBC-1986 | European | Finland | 1985-1986 |  |  |  | Total: 707  Early: 3,180  Late: 676 | Extracted from hospital records | Total: 0.35 (0.28, 0.42)  Early: 0.25 (0.15, 0.35)  Late: 0.44 (0.35, 0.55) |
| PANIC | European | Finland | 1999-2002 |  |  | Total: 346  Early: NA  Late: NA |  | Medical records and parental questionnaire | Total: 0.35 (0.27, 0.44)  Early: NA  Late: NA |
| RAINE | European | Australia | 1989-1991 |  | Total: 1,267  Early: 448  Late: 447 | Total: 1370  Early: 480  Late: 479 |  | Extracted from hospital records | Total: 0.39 (0.30, 0.49)  Early: 0.29 (0.17,0.42)  Late: 0.50 (0.40,0.63) |
| Rhea | European | Greece |  |  | Total: 936  Early: 56  Late: 46 |  | Total: 653  Early: 29  Late: 24 | Trained study personnel | Total: 0.37 (0.26, 0.46)  Early: 0.21 (0.14, 0.32)  Late: 0.44 (0.29, 0.66) |
| STRIP | European | Finland | 1989-1991 |  |  | Total: 470  Early: NA  Late: NA |  | Extracted from hospital records | Total: 0.35 (0.29, 0.48)  Early: NA  Late: NA |
| SWS | European | UK |  |  | Total: 1,702  Early: NA  Late: NA |  | Total: 1,654  Early: NA  Late: NA | Measured by research nurses | Total: 0.38 (0.28, 0.48)  Early: NA  Late: NA |
| TEENAGE | European | Greece | 1993-1998 |  |  | Total: 290  Early: NA  Late: NA |  |  | Total: 0.32 (0.25, 0.42)  Early: NA  Late: NA |

* This is the maximum number of individuals with gestational weight gain and complete data for SNPs (i.e. some SNPs will have a slightly smaller sample size when there is missing genotype data for a subset of individuals)

** GOYA and HIP were designed as case-cohort and case-control studies, respectively. GOYA included cases (BMI 32.6 to 64.4kg/m2) and controls (BMI<32.6km/m^2^) and HIP included of cases of severely obese women (BMI>40kg/m^2^) with lean controls (BMI<25kg/m^2^). In both studies the GWAS results were similar for cases and cohort/controls and all results (including the GWG medians in this table) are for both groups of participants combined. All other studies are general population studies.

**eTable 2: SNPs used in weighted allele scores**

| **Phenotype^ref^** | **Gene** | **Rs number** | **Risk allele** |
| --- | --- | --- | --- |
| Birthweight[^19^](#_ENREF_19) | *CCNL1* | rs900400 | T |
|  | *LCORL* | rs724577 | A |
|  | *CDKAL1* | rs6931514 | A |
|  | *ADCY5* | rs9883204 | T |
|  | *HMGA2* | rs1042725 | C |
|  | *ADRB1* | rs1801253 | C |
|  | *5q11.2* | rs4432842 | T |
| Body mass index (BMI) [^20^](#_ENREF_20) | *FTO* | rs1558902 | A |
|  | *MTCH2, NDUFS3, CUGBP1* | rs3817334 | T |
|  | *FAIM2* | rs7138803 | A |
|  | *SLC39A8* | rs13107325 | T |
|  | *GPRC5B, IQCK* | rs12444979 | C |
|  | *KCTD15* | rs29941 | G |
|  | *SEC16B* | rs543874 | G |
|  | *NEGR1* | rs2815752 | A |
|  | *TNNI3K* | rs1514175 | A |
|  | *PRKD1* | rs11847697 | T |
|  | *CADM2* | rs13078807 | G |
|  | *LRP1B* | rs2890652 | C |
|  | *SH2B1, APOB48R, SULT1A2, AC138894.2, ATXN2L, TUFM* | rs7359397 | T |
|  | *RBJ, ADCY3, POMC* | rs713586 | C |
|  | *TMEM160, ZC3H4* | rs3810291 | A |
|  | *MC4R* | rs571312 | A |
|  | *FANCL* | rs887912 | T |
|  | *TMEM18* | rs2867125 | C |
|  | *GNPDA2* | rs10938397 | G |
|  | *PTBP2* | rs1555543 | C |
|  | *MAP2K5, LBXCOR1* | rs2241423 | G |
|  | *NUDT3, HMGA1* | rs206936 | G |
|  | *QPCTL, GIPR* | rs2287019 | C |
|  | *TFAP2B* | rs987237 | G |
|  | *ZNF608* | rs4836133 | A |
|  | *MTIF3, GTF3A* | rs4771122 | G |
|  | *RPL27A, TUB* | rs4929949 | C |
|  | *FLJ35779, HMGCR* | rs2112347 | T |
|  | *LRRN6C* | rs10968576 | G |
|  | *NRXN3* | rs10150332 | C |
|  | *BDNF* | rs10767664 | A |
|  | *ETV5* | rs9816226 | T |
| Waist-Hip Ratio (WHR) [^21^](#_ENREF_21) | *RSPO3* | rs9491696 | G |
|  | *ADAMTS9* | rs6795735 | C |
|  | *TBX15-WARS2* | rs984222 | G |
|  | *CPEB4* | rs6861681 | A |
|  | *VEGFA* | rs6905288 | A |
|  | *NISCH-STAB1* | rs6784615 | T |
|  | *GRB14* | rs10195252 | T |
|  | *HOXC13* | rs1443512 | A |
|  | *DNM3-PIGC* | rs1011731 | G |
|  | *LYPLAL1* | rs4846567 | G |
|  | *ITPR2-SSPN* | rs718314 | G |
|  | *LY86* | rs1294421 | G |
|  | *ZNRF3-KREMEN1* | rs4823006 | A |
|  | *NFE2L3* | rs1055144 | T |
| Height[^22^](#_ENREF_22) | *TET2* | rs10010325 | A |
|  |  | rs10037512 | T |
|  |  | rs1013209 | C |
|  |  | rs10152591 | A |
|  | *PIP4K2B* | rs1043515 | G |
|  | *TSEN15* | rs1046934 | C |
|  | *MICAL1/ZBTB24* | rs1046943 | A |
|  | *ID4* | rs1047014 | C |
|  |  | rs10748128 | T |
|  | *SLCO1C1* | rs10770705 | A |
|  |  | rs10799445 | A |
|  | *PTPRJ* | rs10838801 | G |
|  | *DTL* | rs10863936 | G |
|  | *FAM69A* | rs10874746 | C |
|  | *SOCS2* | rs11107116 | T |
|  |  | rs11118346 | C |
|  | *PCSK5* | rs11144688 | G |
|  |  | rs11205277 | G |
|  | *ADAMTSL3* | rs11259936 | C |
|  | *CPN1* | rs11599750 | C |
|  | *NARFL* | rs11648796 | G |
|  | *EIF2AK3* | rs11684404 | C |
|  |  | rs1173727 | T |
|  | *SBNO1* | rs11830103 | G |
|  | *KCNJ16* | rs11867479 | T |
|  | *SLC38A9* | rs11958779 | G |
|  |  | rs12153391 | C |
|  |  | rs12470505 | T |
|  |  | rs12474201 | A |
|  | *IGF2BP3* | rs12534093 | T |
|  |  | rs1257763 | A |
|  | *ZFAT* | rs12680655 | C |
|  | *SEPT2* | rs12694997 | G |
|  | *MYO9A* | rs12902421 | C |
|  | *DOT1L* | rs12982744 | G |
|  | *DOCK3* | rs13088462 | C |
|  | *FER* | rs13177718 | C |
|  | *PAPPA2* | rs1325598 | G |
|  | *NUCB2* | rs1330 | T |
|  | *DIRC3* | rs1351164 | T |
|  | *HMGA2* | rs1351394 | T |
|  |  | rs143384 | G |
|  |  | rs1468758 | C |
|  |  | rs1490384 | T |
|  | *RAD51B* | rs1570106 | C |
|  |  | rs1582931 | G |
|  |  | rs1659127 | A |
|  | *ACAN* | rs16942341 | C |
|  | *CYP19A1* | rs16964211 | G |
|  |  | rs17081935 | T |
|  | *JAZF1* | rs1708299 | A |
|  | *ATP5SL* | rs17318596 | A |
|  | *DNM3* | rs17346452 | C |
|  |  | rs1738475 | C |
|  |  | rs17391694 | T |
|  | *LOC101929155* | rs1741344 | C |
|  |  | rs17511102 | T |
|  |  | rs17780086 | A |
|  |  | rs17782313 | C |
|  | *SUCLG2* | rs17806888 | T |
|  |  | rs1814175 | T |
|  |  | rs1950500 | T |
|  | *STAT2* | rs2066807 | G |
|  | *ZNF652* | rs2072153 | C |
|  |  | rs2079795 | T |
|  |  | rs2093210 | C |
|  |  | rs2110001 | G |
|  |  | rs2145272 | G |
|  |  | rs2145998 | T |
|  |  | rs2154319 | C |
|  | *KCNQ1* | rs2237886 | T |
|  | *SLBP* | rs2247341 | A |
|  | *MICA* | rs2256183 | A |
|  |  | rs227724 | T |
|  | *MYO9B* | rs2279008 | T |
|  | *MFAP2* | rs2284746 | G |
|  |  | rs2336725 | C |
|  | *CAMKMT* | rs2341459 | T |
|  | *ZFAS1* | rs237743 | A |
|  |  | rs2580816 | C |
|  |  | rs2597513 | C |
|  |  | rs2629046 | T |
|  | *CCDC91* | rs2638953 | C |
|  |  | rs2665838 | G |
|  |  | rs26868 | A |
|  | *LOC553103* | rs274546 | G |
|  |  | rs2778031 | T |
|  |  | rs2780226 | C |
|  |  | rs2834442 | A |
|  | *ETV6* | rs2856321 | G |
|  | *IGF1R* | rs2871865 | C |
|  |  | rs310405 | A |
|  | *ANKRD13B/GIT1* | rs3110496 | G |
|  |  | rs3118905 | G |
|  |  | rs3129109 | C |
|  | *ATAD5* | rs3764419 | C |
|  | *SSSCA1-AS1/SSSCA1* | rs3782089 | C |
|  | *EFEMP1* | rs3791675 | C |
|  | *BMP6* | rs3812163 | T |
|  | *MYO1F* | rs4072910 | G |
|  | *CDK6* | rs42235 | T |
|  | *FGFR4* | rs422421 | C |
|  | *PRKCZ* | rs425277 | T |
|  | *SLIT3* | rs4282339 | G |
|  |  | rs4470914 | T |
|  |  | rs4601530 | C |
|  | *NME1-NME2/NME2* | rs4605213 | C |
|  | *KCNJ12* | rs4640244 | A |
|  | *DNAJC27* | rs4665736 | T |
|  | *PTCH1* | rs473902 | T |
|  | *CABLES1* | rs4800452 | T |
|  | *SYN3* | rs4821083 | T |
|  |  | rs494459 | T |
|  | *ADAMTS17* | rs4965598 | C |
|  | *ACBD4* | rs4986172 | C |
|  | *OR4A5* | rs5017948 | A |
|  |  | rs526896 | T |
|  | *ESR1* | rs543650 | G |
|  | *GHSR* | rs572169 | T |
|  | *PML* | rs5742915 | C |
|  | *SERPINH1* | rs634552 | T |
|  |  | rs6439167 | C |
|  |  | rs6449353 | T |
|  |  | rs6457620 | G |
|  |  | rs6457821 | C |
|  |  | rs6470764 | C |
|  |  | rs6473015 | C |
|  | *FLI1* | rs654723 | A |
|  | *L3MBTL3* | rs6569648 | C |
|  | *TGFB2* | rs6684205 | G |
|  | *RP11-76N22.2* | rs6699417 | T |
|  | *LTBP1* | rs6714546 | G |
|  | *GFPT2* | rs6879260 | C |
|  |  | rs6959212 | C |
|  |  | rs7027110 | A |
|  | *RHOD* | rs7112925 | C |
|  | *TRIP11* | rs7155279 | G |
|  |  | rs7178424 | C |
|  |  | rs720390 | A |
|  | *ZBTB38* | rs724016 | G |
|  | *ZNF341* | rs7274811 | G |
|  |  | rs7319045 | A |
|  |  | rs7332115 | G |
|  |  | rs7460090 | T |
|  | *FUBP3* | rs7466269 | A |
|  | *NFIC* | rs7507204 | C |
|  | *PAPPA* | rs751543 | T |
|  | *LIN28A* | rs7532866 | A |
|  |  | rs7567288 | C |
|  | *PDE11A* | rs7567851 | C |
|  | *HHIP-AS1/HHIP* | rs7689420 | C |
|  |  | rs7697556 | T |
|  |  | rs7759938 | C |
|  |  | rs7763064 | G |
|  | *QSOX2* | rs7849585 | T |
|  |  | rs7853377 | G |
|  |  | rs7864648 | T |
|  |  | rs788867 | G |
|  |  | rs7909670 | C |
|  | *LOC102723893/TEAD1* | rs7926971 | G |
|  |  | rs7971536 | T |
|  | *GNA12* | rs798489 | C |
|  | *CTU2* | rs8052560 | A |
|  | *HIST1H2AD/HIST1H2BF/HIST1H3D* | rs806794 | A |
|  |  | rs8181166 | C |
|  |  | rs822552 | G |
|  | *LTBP2* | rs862034 | G |
|  |  | rs889014 | C |
|  | *INSR* | rs891088 | G |
|  |  | rs9360921 | G |
|  |  | rs9428104 | G |
|  | *TULP4* | rs9456307 | T |
|  | *SUPT3H* | rs9472414 | T |
|  | *WWC2* | rs955748 | G |
|  |  | rs961764 | G |
|  | *FAM208A* | rs9835332 | G |
|  | *PCCB* | rs9844666 | G |
|  | *RYBP* | rs9863706 | C |
|  | *DYM* | rs9967417 | G |
|  | *IPPK* | rs9969804 | A |
| Blood pressure[^23^](#_ENREF_23)^,^[^24^](#_ENREF_24) | *TBX5-TBX3* | rs10850411 | T |
|  | *CYP17A1-NT5C2* | rs11191548 | T |
|  | *NPR3-C5orf23* | rs1173771 | G |
|  | *EBF1* | rs11953630 | C |
|  | *ZNF652* | rs12940887 | T |
|  | *SLC4A7* | rs13082711 | C |
|  | *SLC39A8* | rs13107325 | C |
|  | *GUCY1A3-GUCY1B3* | rs13139571 | C |
|  | *JAG1* | rs1327235 | G |
|  | *CYP1A1-ULK3* | rs1378942 | C |
|  | *FGF5* | rs1458038 | T |
|  | *ATP2B1* | rs17249754 | G |
|  | *MTHFR-NPPB* | rs17367504 | A |
|  | *GOSR2* | rs17608766 | C |
|  | *HFE* | rs1799945 | G |
|  | *CACNB2(3')* | rs1813353 | T |
|  | *FURIN-FES* | rs2521501 | T |
|  | *MOV10* | rs2932538 | G |
|  | *SH2B3* | rs3184504 | T |
|  | *ULK4* | rs3774372 | C |
|  | *PLEKHA7* | rs381815 | T |
|  | *MECOM* | rs419076 | T |
|  | *CACNB2(5')* | rs4373814 | C |
|  | *C10orf107* | rs4590817 | G |
|  | *GNAS-EDN3* | rs6015450 | G |
|  | *FLJ32810-TMEM133* | rs633185 | C |
|  | *ADM* | rs7129220 | A |
|  | *BAT2-BAT5* | rs805303 | G |
|  | *PLCE1* | rs932764 | G |
| Glucose[^25^](#_ENREF_25) | *FADS1* | rs174550 | T |
|  | *GCK* | rs4607517 | A |
|  | *CRY2* | rs11605924 | A |
|  | *MTNR1B* | rs10830963 | G |
|  | *GCKR* | rs780094 | C |
|  | *G6PC2* | rs560887 | C |
|  | *DGKB-TMEM195* | rs2191349 | T |
|  | *GLIS3* | rs7034200 | A |
|  | *PROX1* | rs340874 | C |
|  | *MADD* | rs7944584 | A |
|  | *SLC2A2* | rs11920090 | T |
|  | *ADRA2A* | rs10885122 | G |
|  | *ADCY5* | rs11708067 | A |
|  | *C2CD4B* | rs11071657 | A |
| Type 2 Diabetes[^26^](#_ENREF_26) | *THADA* | rs10203174 | C |
|  | *GCK* | rs10278336 | A |
|  | *CILP2* | rs10401969 | C |
|  | *GLIS3* | rs10758593 | A |
|  | *CDKN2A/B* | rs10811661 | T |
|  | *MTNR1B* | rs10830963 | G |
|  | *KLHDC5* | rs10842994 | C |
|  | *NOTCH2* | rs10923931 | T |
|  | *CCND2* | rs11063069 | G |
|  | *HHEX/IDE* | rs1111875 | C |
|  | *CDC123/CAMK1D* | rs11257655 | T |
|  | *ZFAND6* | rs11634397 | G |
|  | *ADCY5* | rs11717195 | T |
|  | *VPS26A* | rs12242953 | G |
|  | *HNF1A (TCF1)* | rs12427353 | G |
|  | *PSMD6* | rs12497268 | G |
|  | *ZMIZ1* | rs12571751 | A |
|  | *PRC1* | rs12899811 | G |
|  | *MC4R* | rs12970134 | A |
|  | *KLF14* | rs13233731 | G |
|  | *GRB14* | rs13389219 | C |
|  | *SPRY2* | rs1359790 | G |
|  | *UBE2E2* | rs1496653 | A |
|  | *ARAP1 (CENTD2)* | rs1552224 | A |
|  | *KCNQ1* | rs163184 | G |
|  | *PTPRD* | rs16927668 | T |
|  | *DGKB* | rs17168486 | T |
|  | *ST64GAL1* | rs17301514 | A |
|  | *TLE4* | rs17791513 | A |
|  | *GCC1* | rs17867832 | T |
|  | *PPARG* | rs1801282 | C |
|  | *AP3S2* | rs2007084 | G |
|  | *PROX1* | rs2075423 | G |
|  | *HMGA2* | rs2261181 | T |
|  | *DUSP8* | rs2334499 | T |
|  | *BCL11A* | rs243088 | T |
|  | *SRR* | rs2447090 | A |
|  | *TLE1* | rs2796441 | G |
|  | *IRS1* | rs2943640 | C |
|  | *KCNK16* | rs3734621 | C |
|  | *SLC30A8* | rs3802177 | G |
|  | *ZFAND3* | rs4299828 | A |
|  | *IGF2BP2* | rs4402960 | T |
|  | *WFS1* | rs4458523 | G |
|  | *C2CD4A* | rs4502156 | T |
|  | *ANKRD55* | rs459193 | G |
|  | *HNF4A* | rs4812829 | A |
|  | *ANK1* | rs516946 | C |
|  | *KCNJ11* | rs5215 | C |
|  | *ADAMTS9* | rs6795735 | C |
|  | *MAEA* | rs6819243 | T |
|  | *ZBED3* | rs6878122 | G |
|  | *HMG20A* | rs7177055 | A |
|  | *BCAR1* | rs7202877 | T |
|  | *RBMS1* | rs7569522 | A |
|  | *CDKAL1* | rs7756992 | G |
|  | *GCKR* | rs780094 | C |
|  | *TP53INP1* | rs7845219 | T |
|  | *TCF7L2* | rs7903146 | T |
|  | *TSPAN8/LGR5* | rs7955901 | C |
|  | *GIPR* | rs8108269 | G |
|  | *PEPD* | rs8182584 | T |
|  | *JAZF1* | rs849135 | G |
|  | *FTO* | rs9936385 | C |
| Vitamin D[^27^](#_ENREF_27) | *DHCR7/NADSYN1* | rs12785878 | A |
|  | *CYP2R1* | rs10741657 | T |

**eTable 3: Full results from M-GCTA for 4,078 maternal-offspring pairs from ALSPAC**

| Trait | Model | Maternal | Offspring | Covariance | LRT | P-Value |
| --- | --- | --- | --- | --- | --- | --- |
| Early GWG | Full | 0.021 (0.113) | 0.000 (0.115) | 0.067 (0.091) | 0.000 | 0.500 |
|  | Cov=0 | 0.094 (0.088) | 0.004 (0.087) | - | 1.140 | 0.100 |
|  | O=Cov=0 | 0.095 (0.085) | - | - | 1.265 | 0.100 |
|  | M=Cov=0 | - | 0.030 (0.084) | - | 0.128 | 0.400 |
| Late GWG | Full | 0.196 (0.113) | 0.161 (0.114) | -0.039 (0.091) | 3.095 | 0.040 |
|  | Cov=0 | 0.166 (0.088) | 0.131 (0.089) | - | 3.733 | 0.030 |
|  | O=Cov=0 | 0.203 (0.085) | - | - | 6.047 | 0.007 |
|  | M=Cov=0 | - | 0.179 (0.085) | - | 4.525 | 0.020 |
| Total GWG | Full | 0.173 (0.112) | 0.045 (0.113) | 0.016 (0.090) | 2.514 | 0.060 |
|  | Cov=0 | 0.186 (0.088) | 0.058 (0.088) | - | 4.698 | 0.020 |
|  | O=Cov=0 | 0.202 (0.085) | - | - | 6.092 | 0.007 |
|  | M=Cov=0 | - | 0.113 (0.085) | - | 1.837 | 0.090 |

Cov: Covariance; O: Offspring; M:Maternal.

**eFigure 1: Q-Q plots of the results from the discovery genome-wide association studies.**

|  | **Early** | **Late** | **Total** |
| --- | --- | --- | --- |
| **Maternal** | **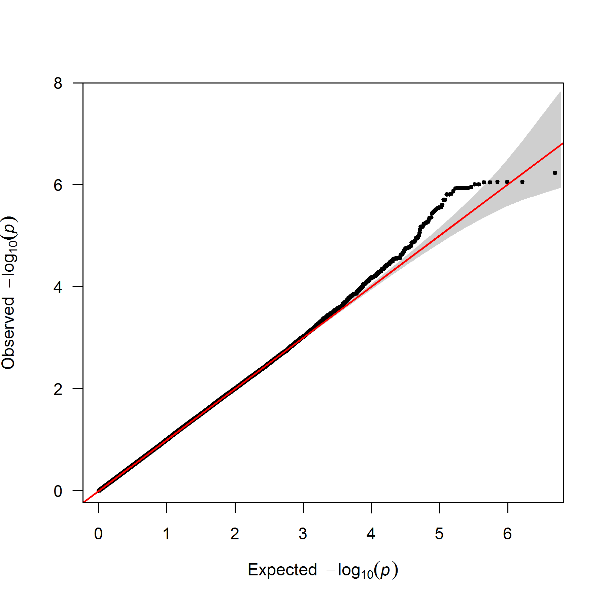**  λ=1.01 | **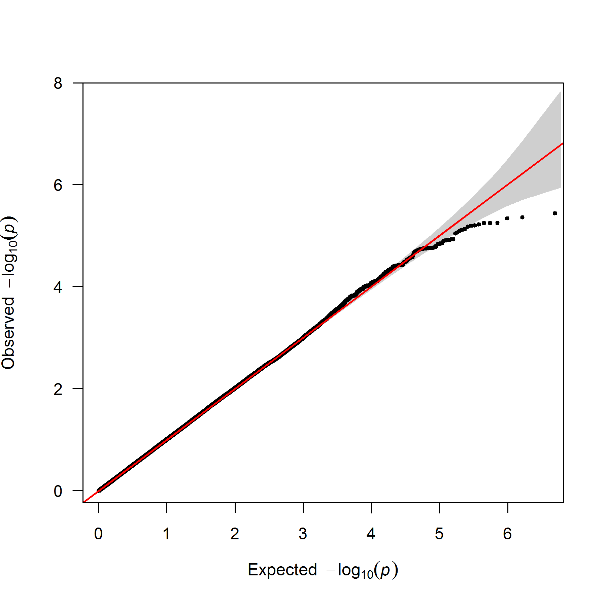**  λ=1.01 | **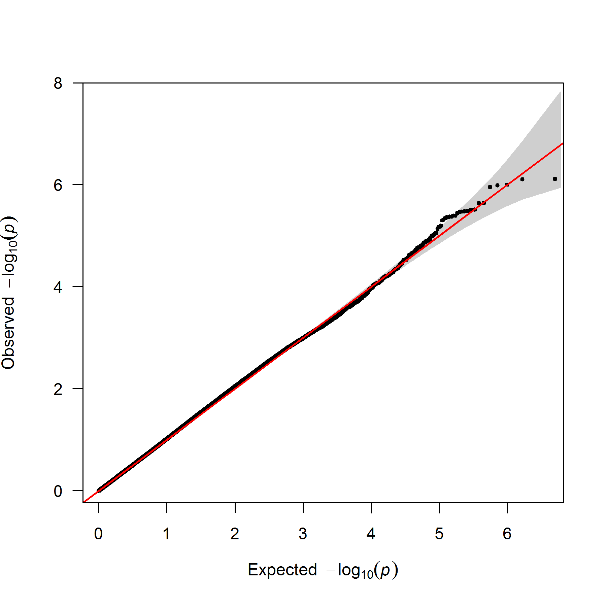**  λ=1.02 |
| **Offspring** | **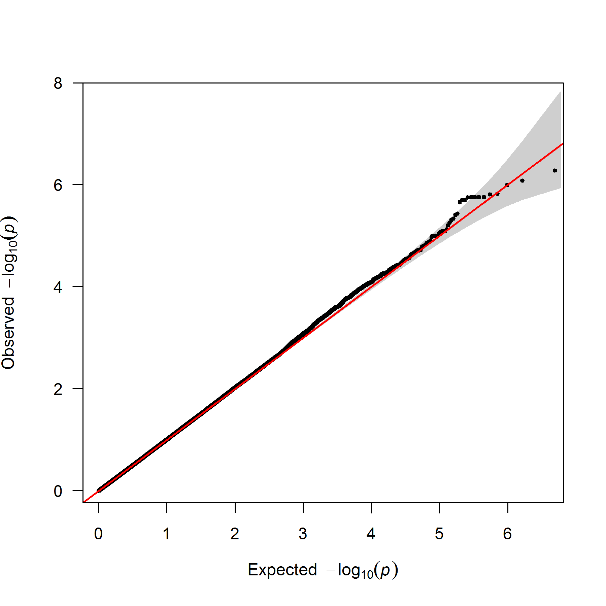**  λ=1.02 | **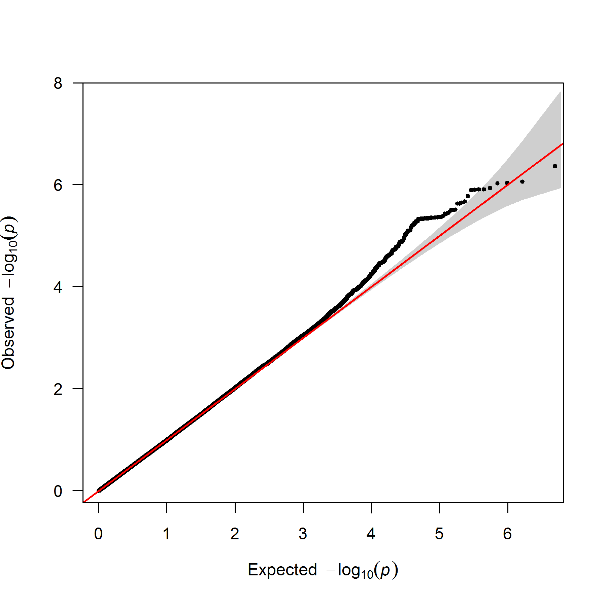**  λ=1.00 | **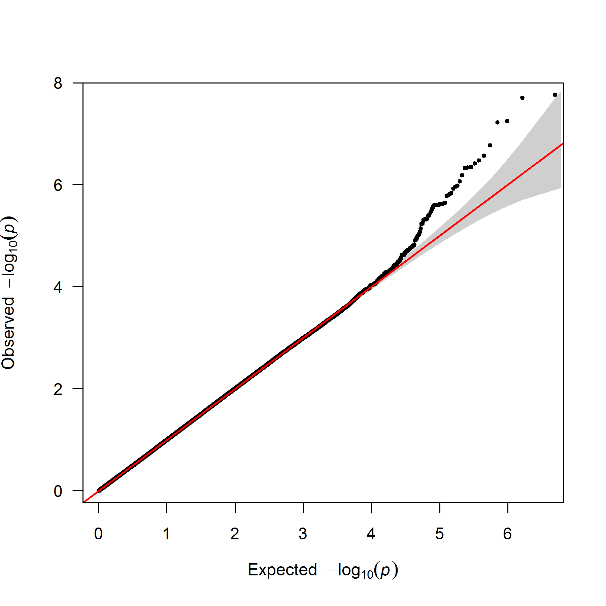**  λ=1.00 |

**eFigure 2: Manhattan plots of the results from the discovery genome-wide association studies.** The red line depicts genome-wide significance at 5x10^-8^, while the blue line is the suggestive level of significance, 1x10^-5^. SNPs reported in less than 50% of the total sample size were excluded from the meta-analysis.

|  | **Early** | **Late** | **Total** |
| --- | --- | --- | --- |
| **Maternal** | **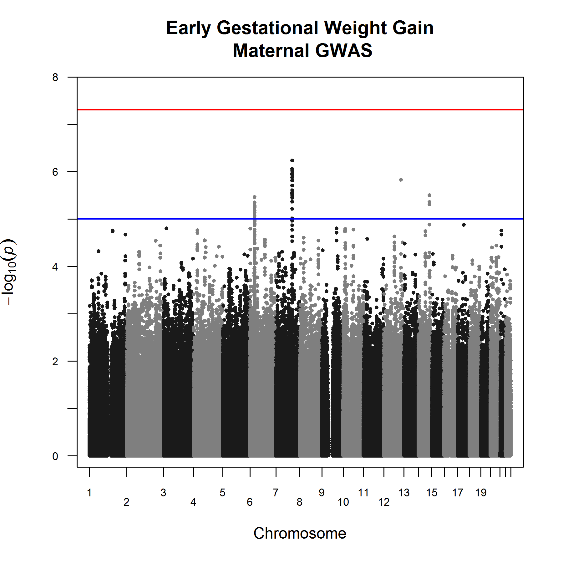** | **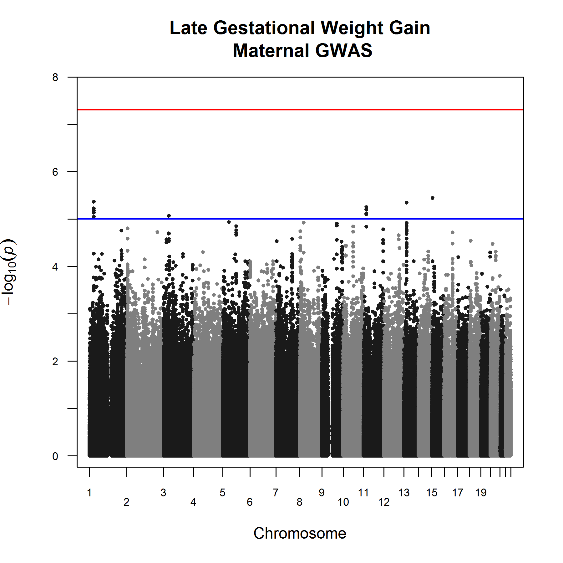** | **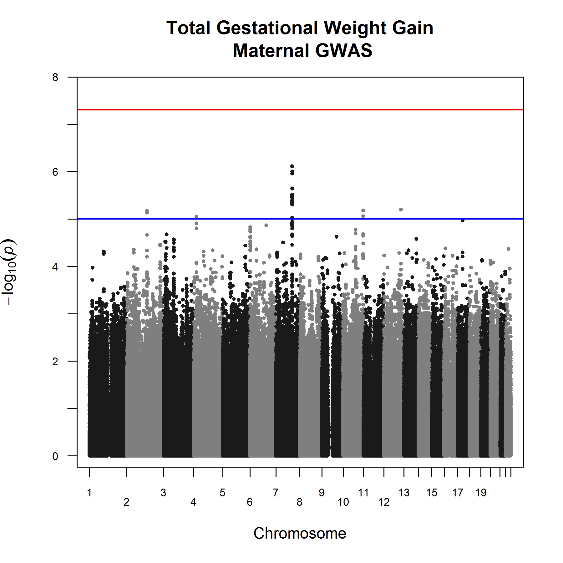** |
| **Offspring** | 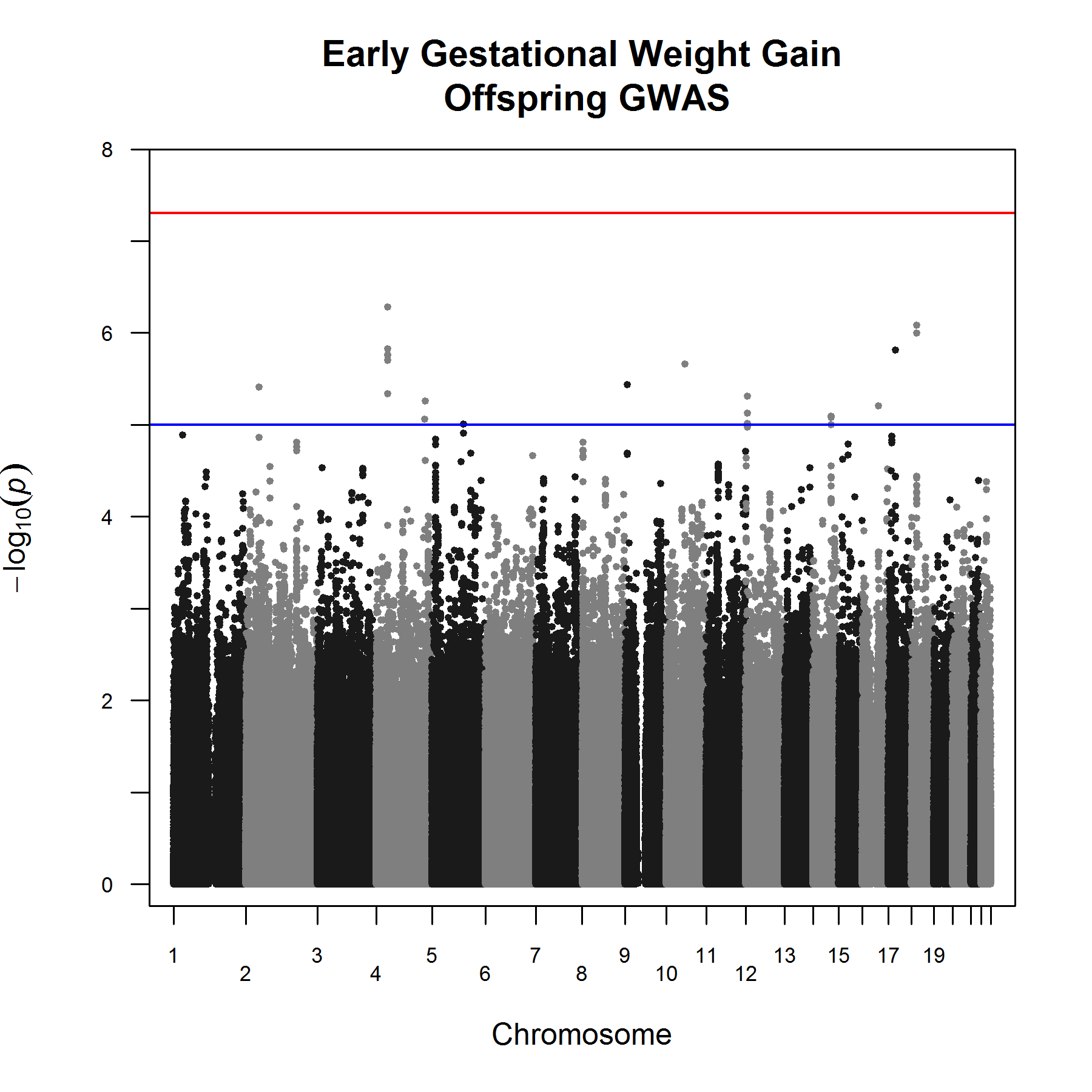 | 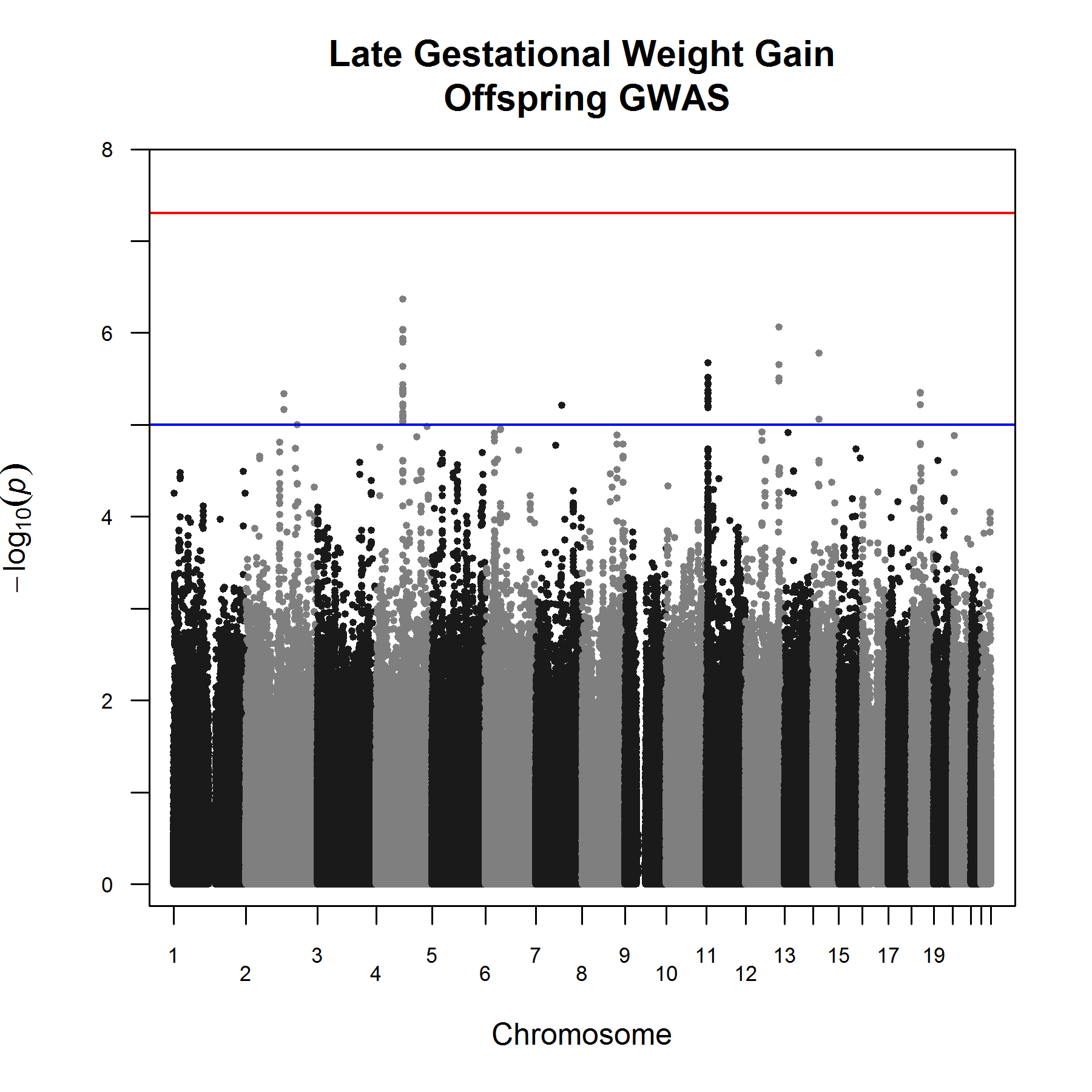 | 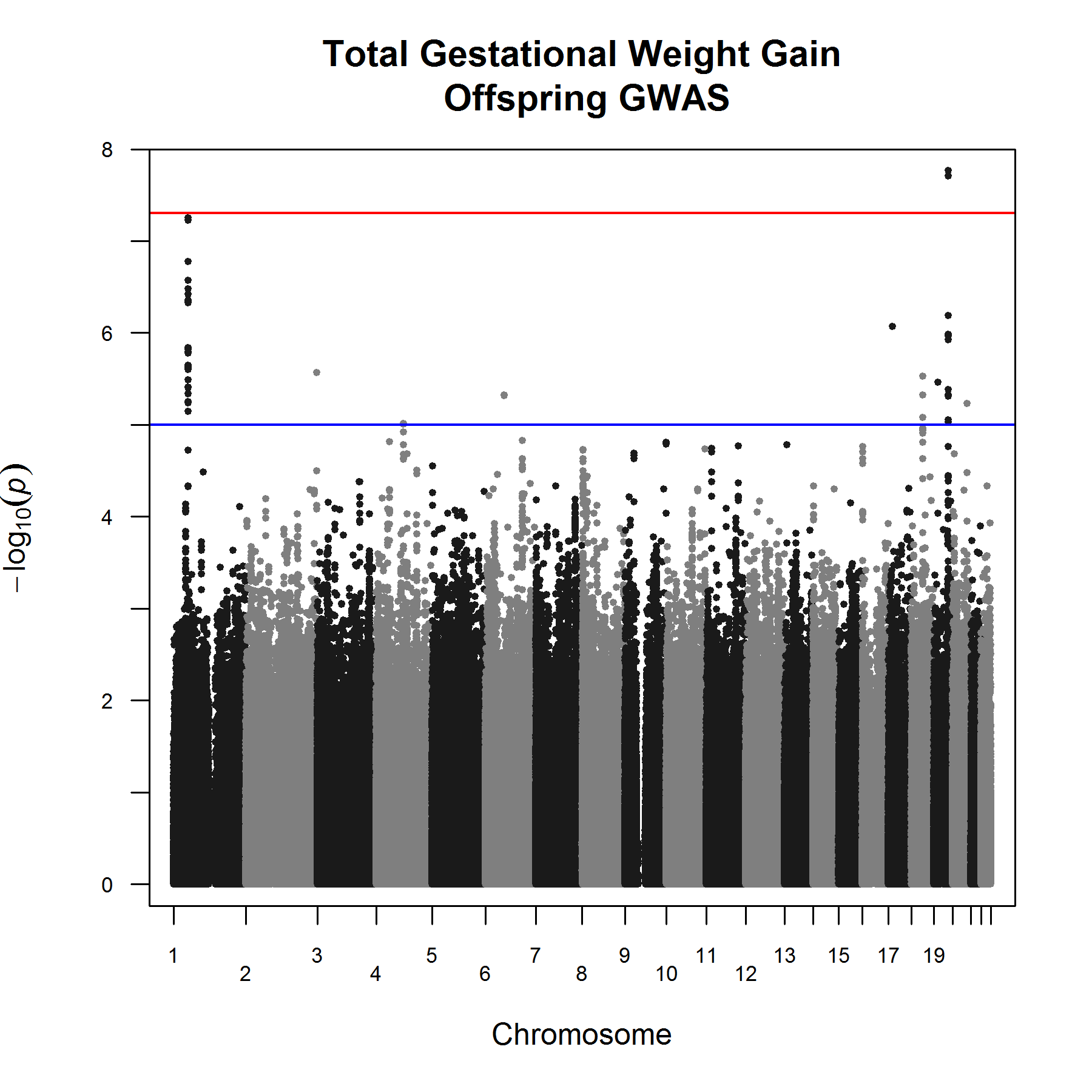 |

**eFigure 3: Summary of previously reported loci for BMI and their effect on GWG using maternal genotype (A-C) and offspring genotype (D-F).** Plots on the left are effect sizes (with SE) on GWG of 32 known BMI loci. Plots on the right are effect sizes (y axis) of previously reported BMI loci[^20^](#_ENREF_20) plotted against changes in GWG z-score (x axis) per BMI raising allele. The colour of each dot indicates the GWG association P-Value: orange, 5×10^−8^≤*P*<0.001; yellow, 0.001≤*P*<0.01; green, 0.01≤*P*<0.05 white, *P*≥0.05

| **A) Early GWG, Maternal genotype**  **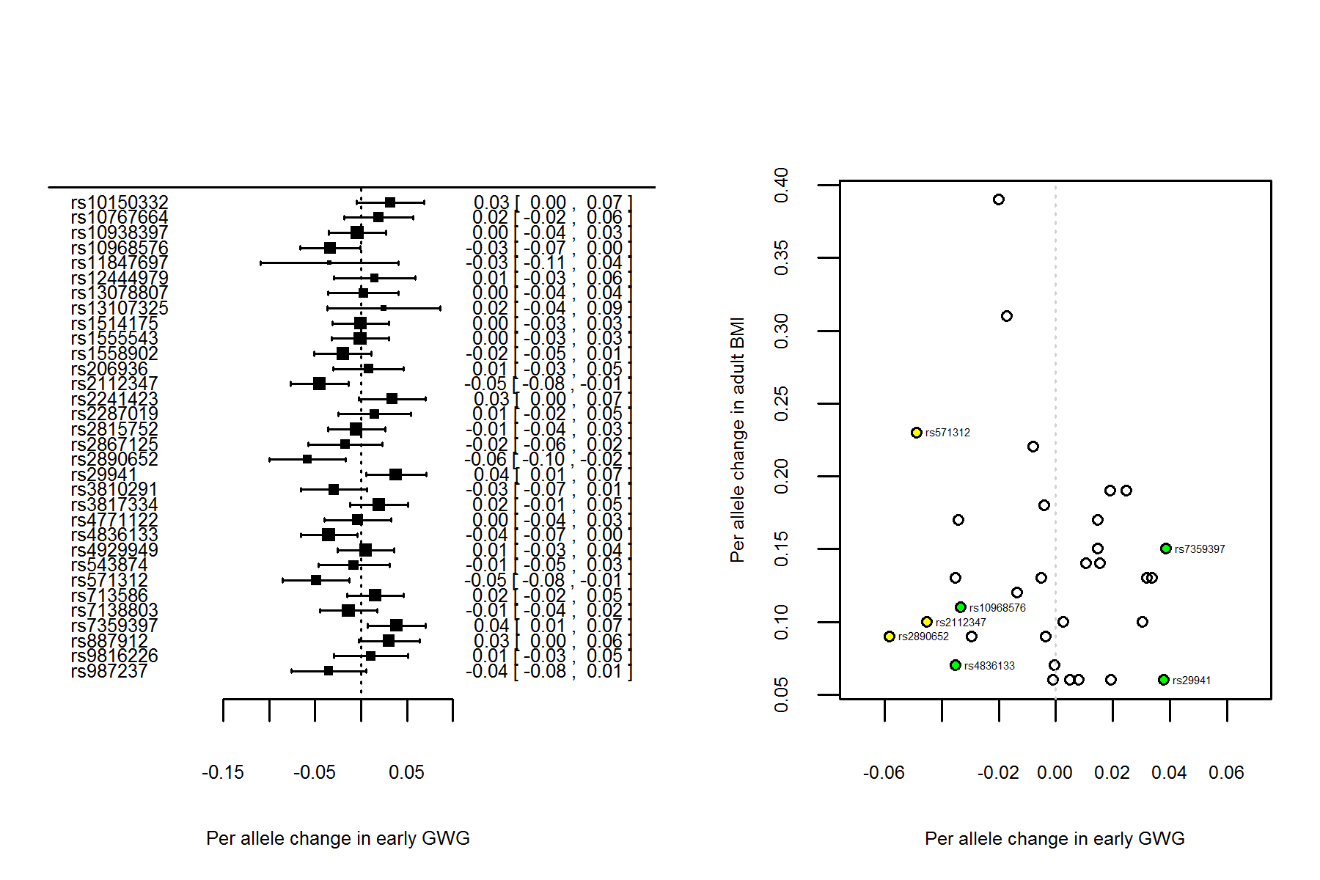** |
| --- |
| **B) Late GWG, Maternal Genotype**  **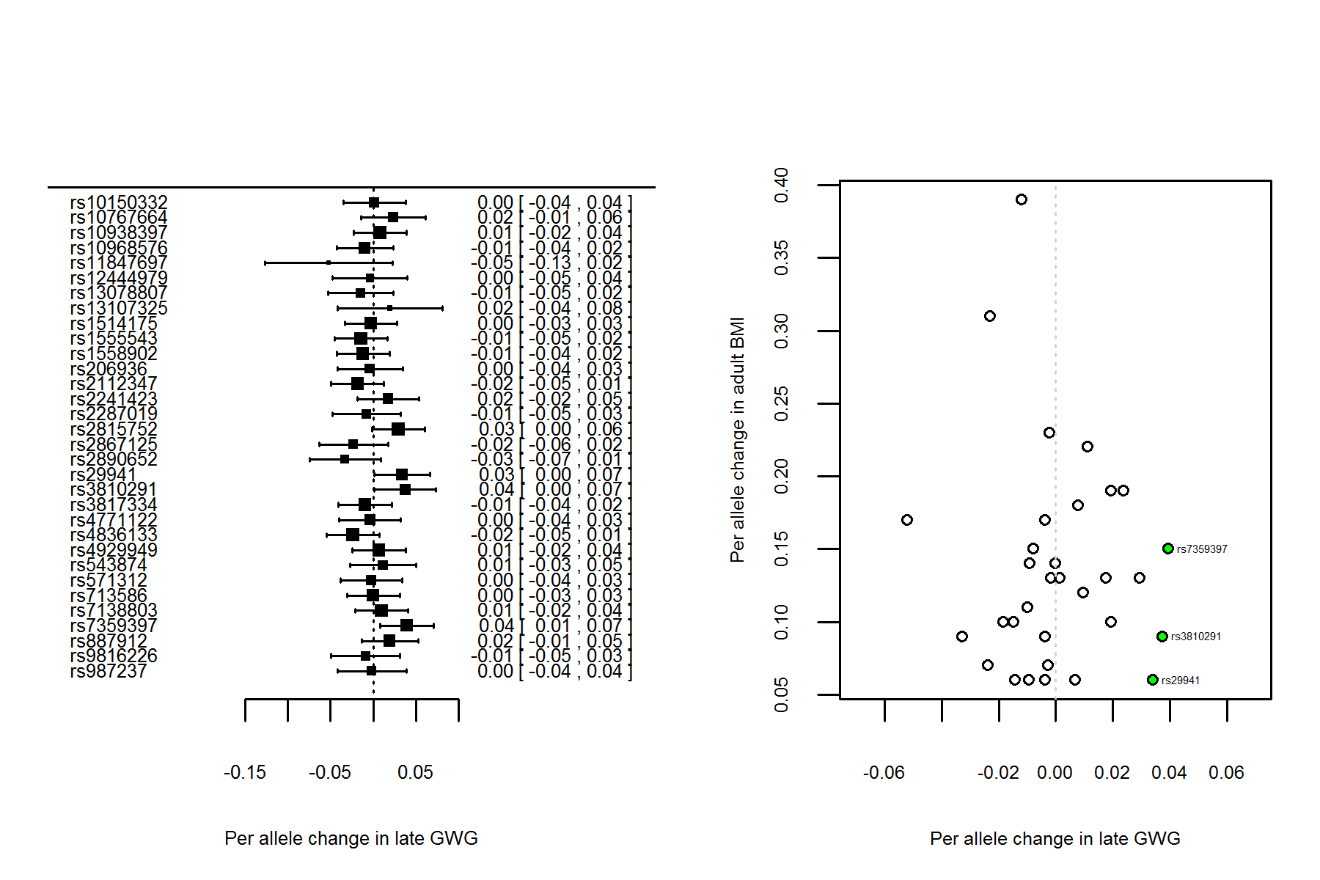** |

| **C) Total GWG, Maternal genotype**  **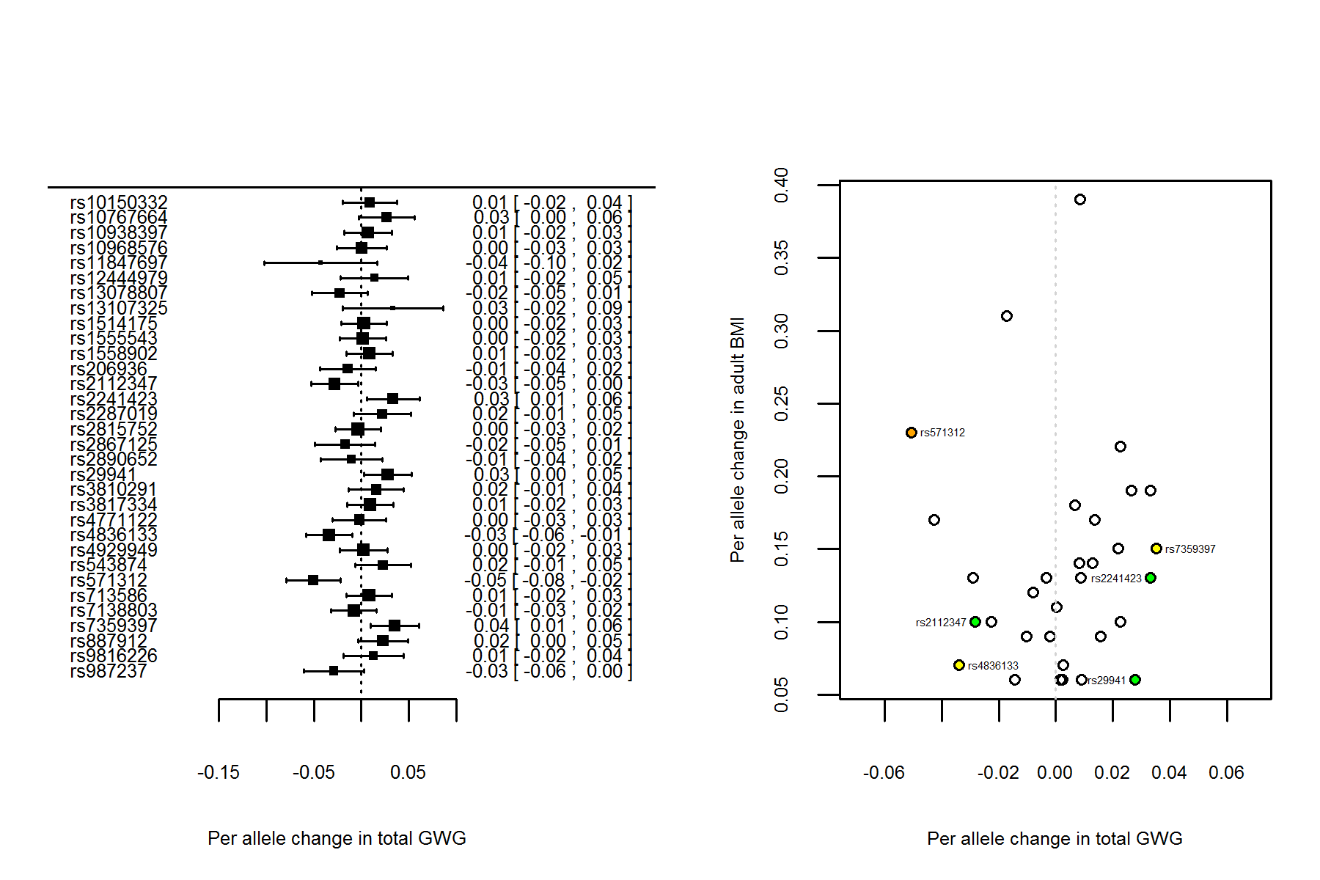** |
| --- |
| **D) Early GWG, Offspring genotype**  **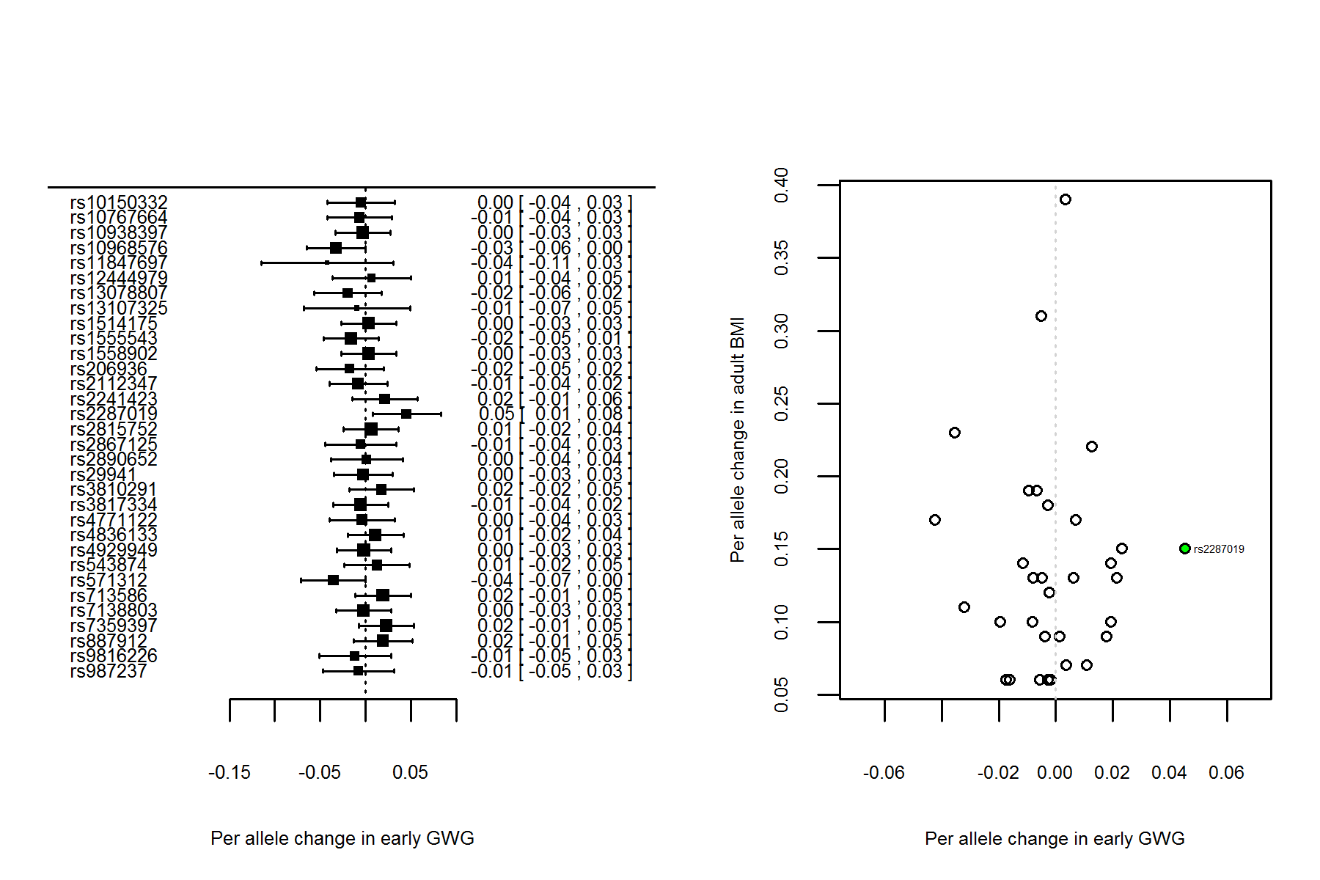** |

| **E) Late GWG, Offspring genotype**  **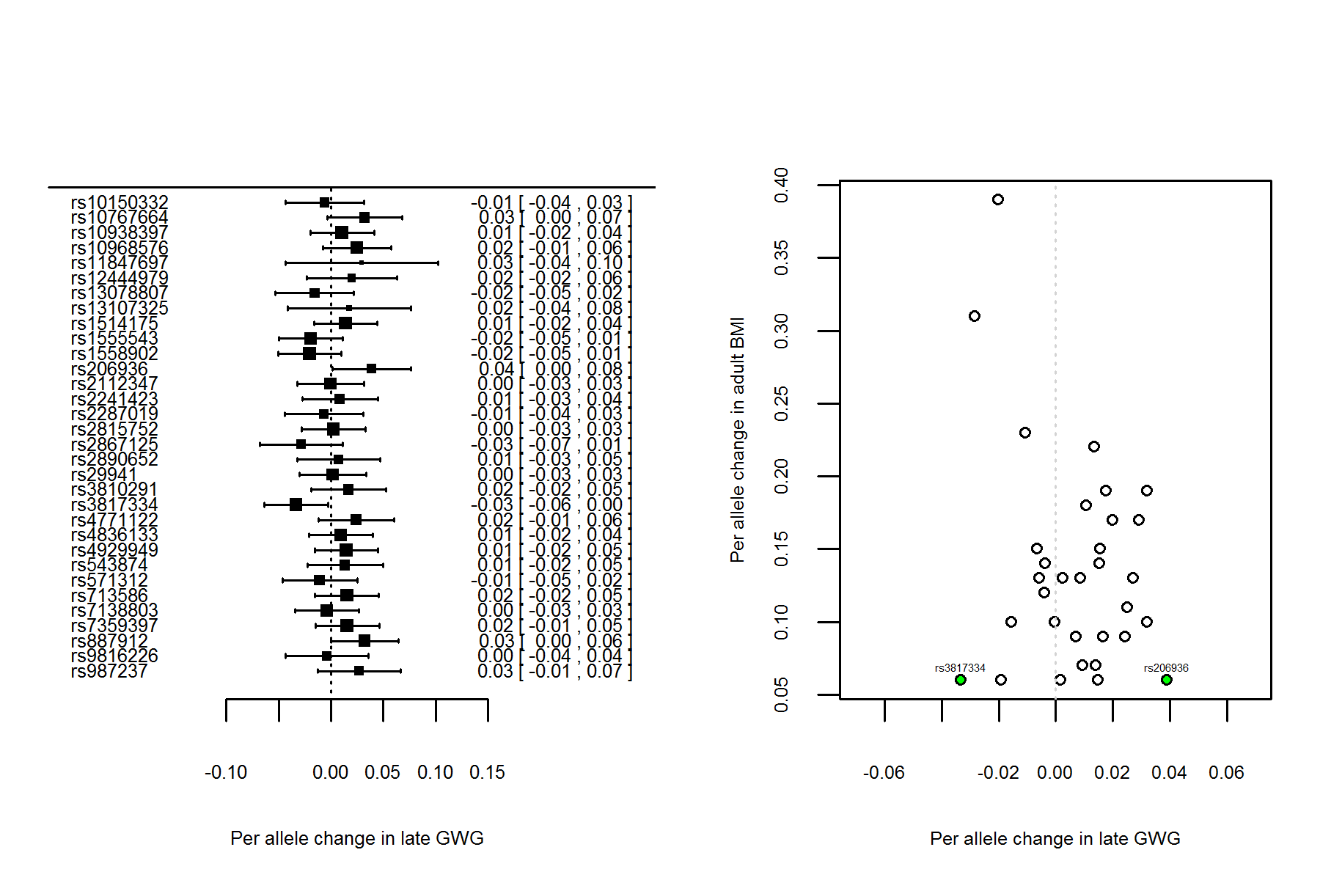** |
| --- |
| **F) Total GWG, Offspring genotype**  **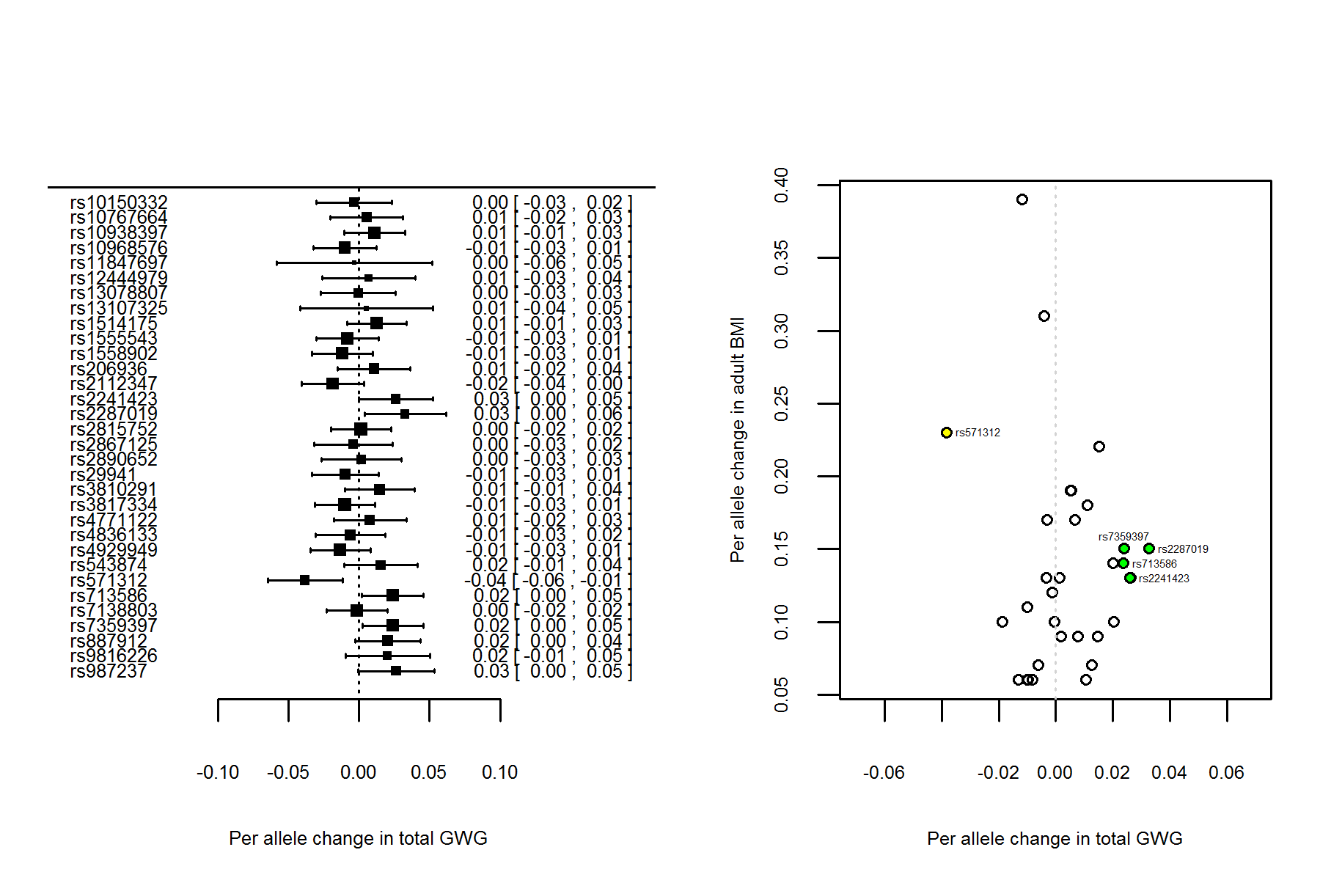** |

**eFigure 4: Summary of previously reported loci for glucose and their effect on GWG using maternal genotype (A-C) and offspring genotype (D-F).** Plots on the left are effect sizes (with SE) on GWG of 14 known glucose loci. Plots on the right are effect sizes (y axis) of previously reported glucose loci[^25^](#_ENREF_25) plotted against changes in GWG z-score (x axis) per gluocse raising allele. The colour of each dot indicates the GWG association P-Value: orange, 5×10^−8^≤*P*<0.001; yellow, 0.001≤*P*<0.01; green, 0.01≤*P*<0.05 white, *P*≥0.05

| **A) Early GWG, Maternal genotype**  **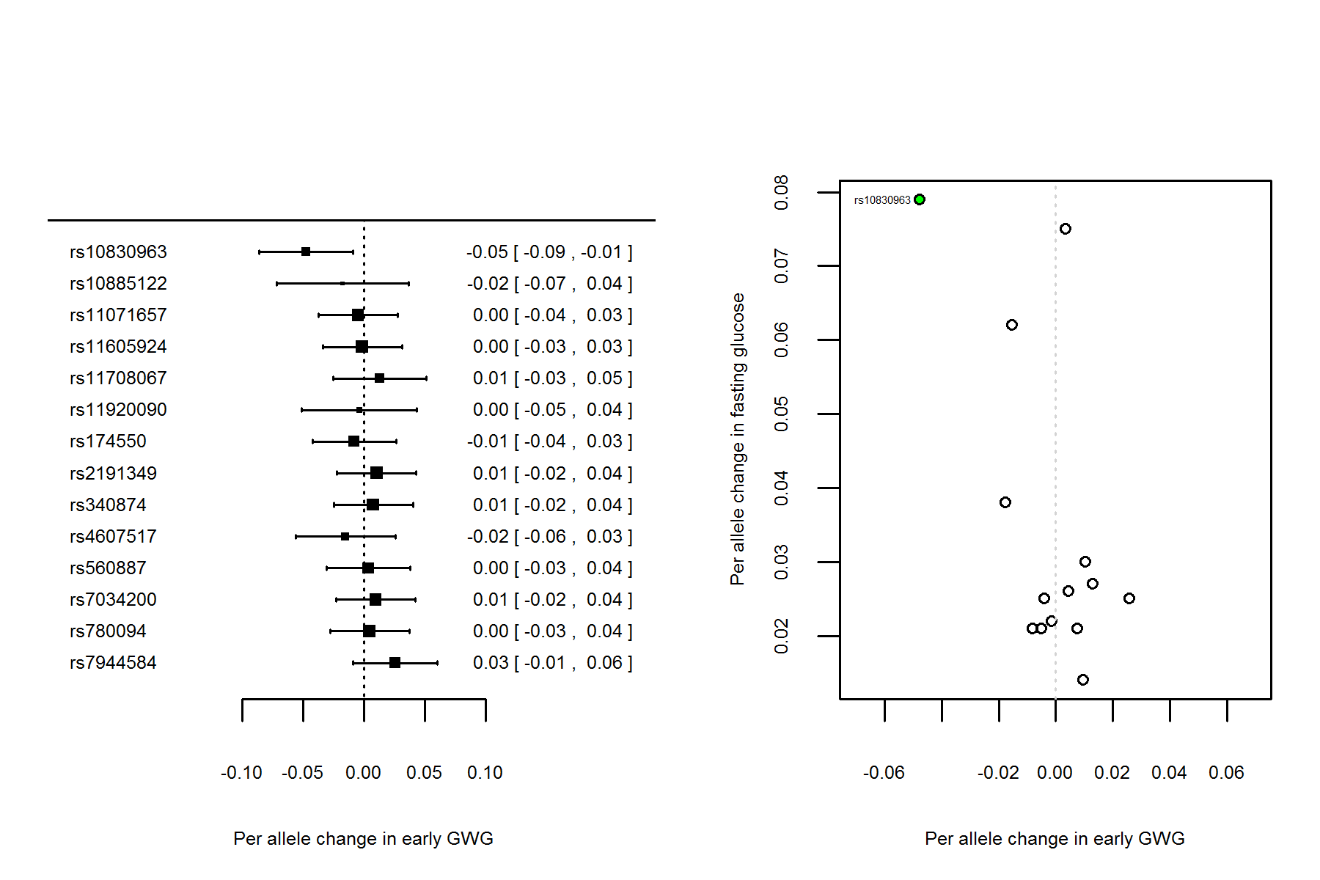** |
| --- |
| **B) Late GWG, Maternal Genotype**  **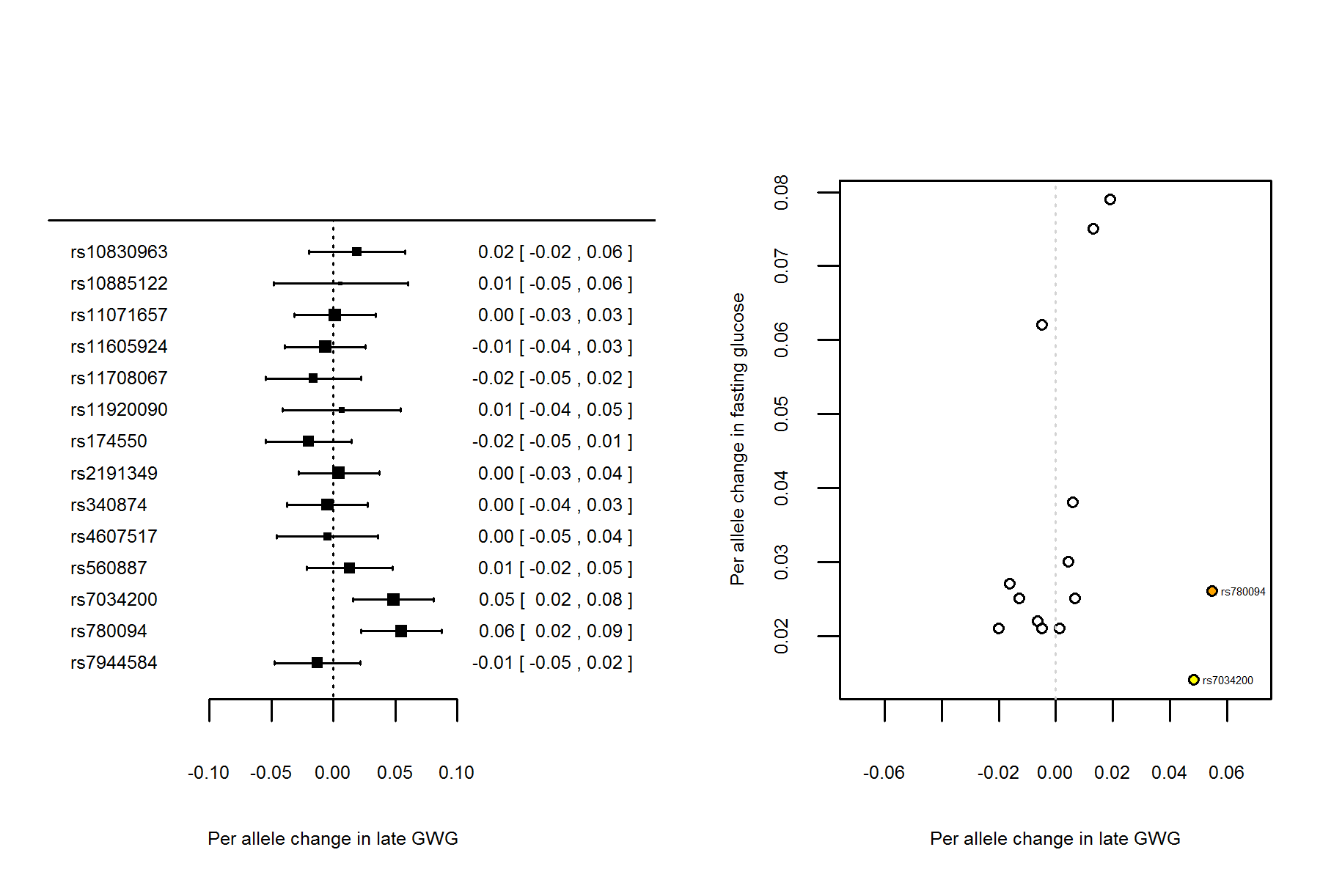** |

| **C) Total GWG, Maternal genotype**  **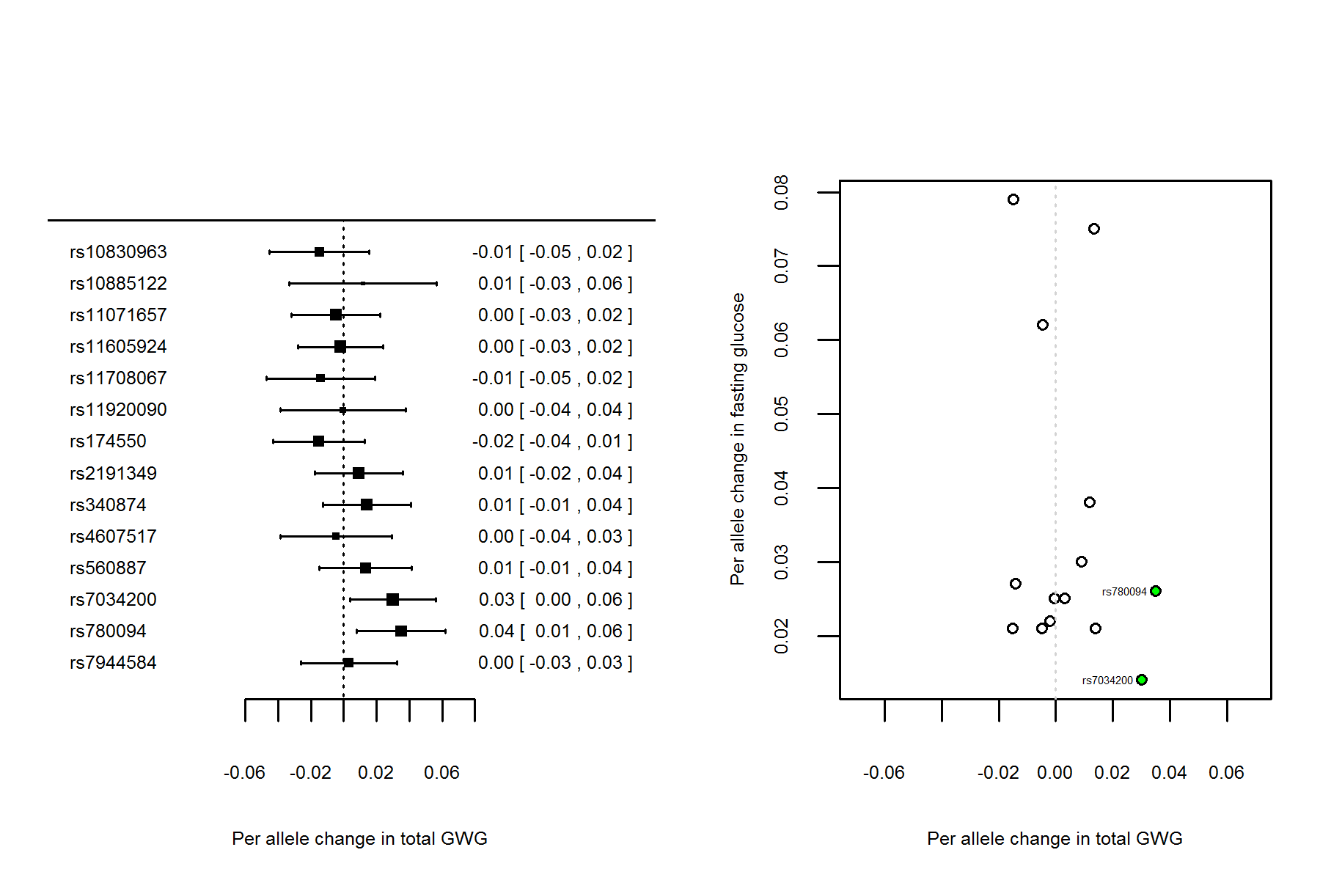** |
| --- |
| **D) Early GWG, Offspring genotype**  **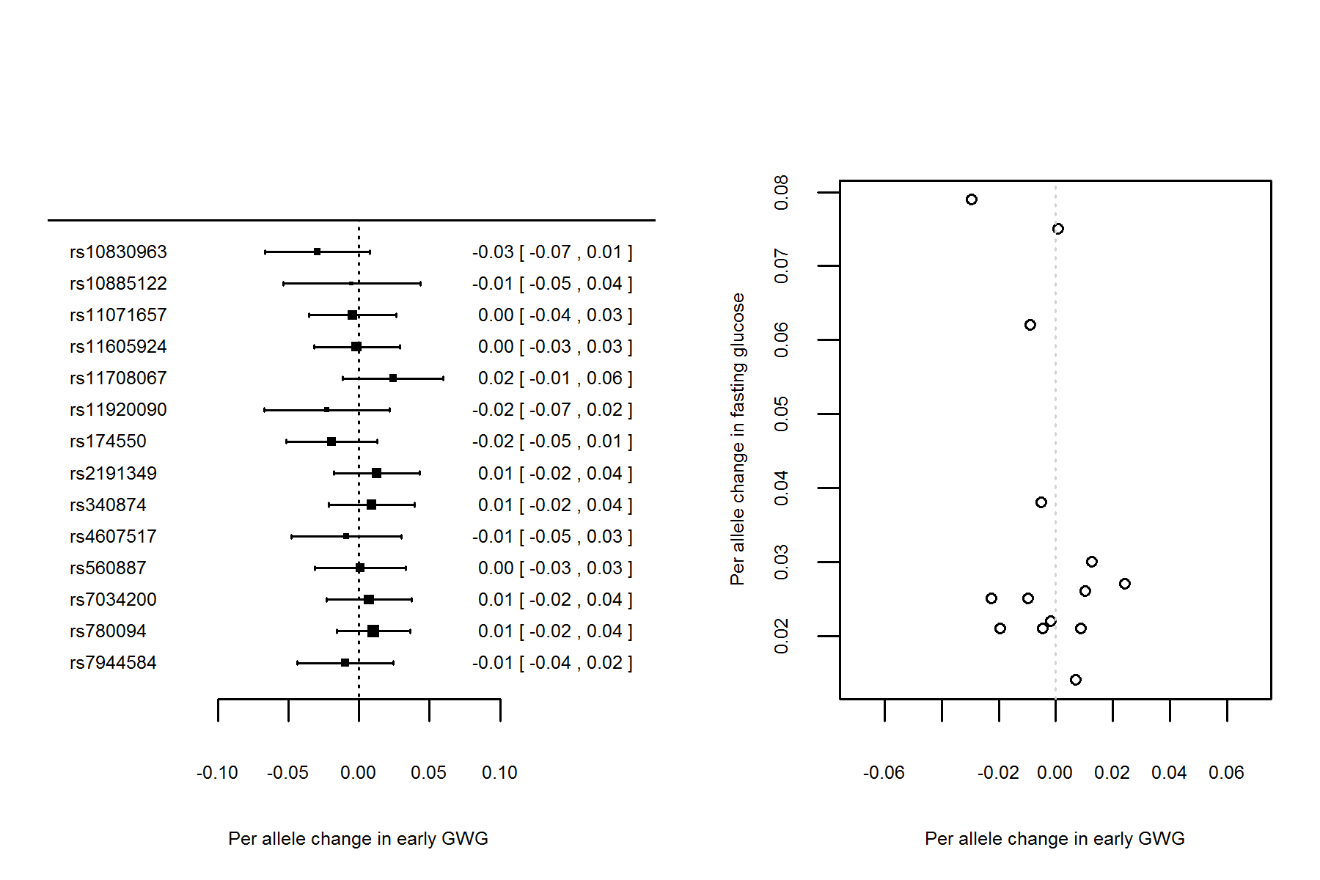** |

| **E) Late GWG, Offspring genotype**  **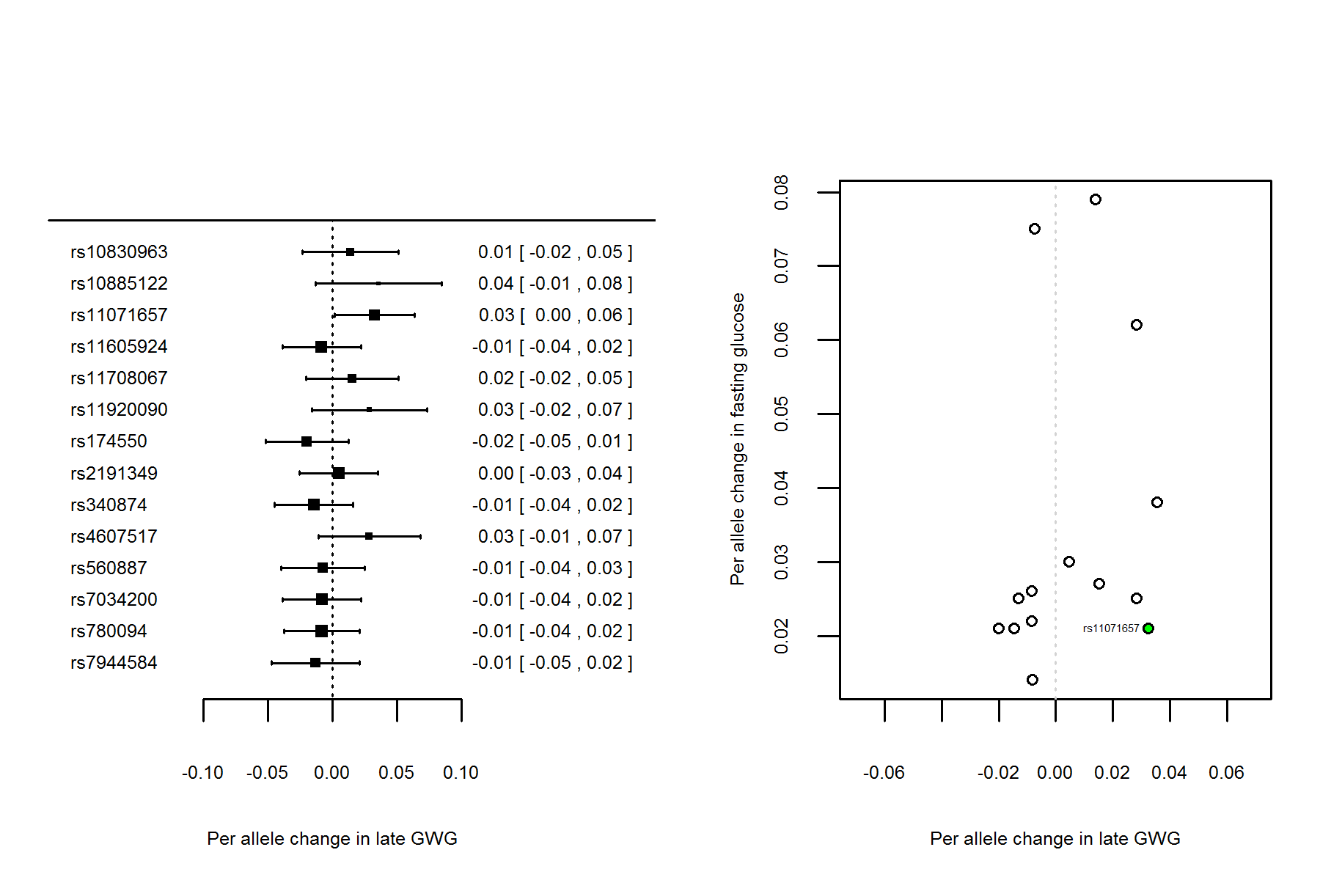** |
| --- |
| **F) Total GWG, Offspring genotype**  **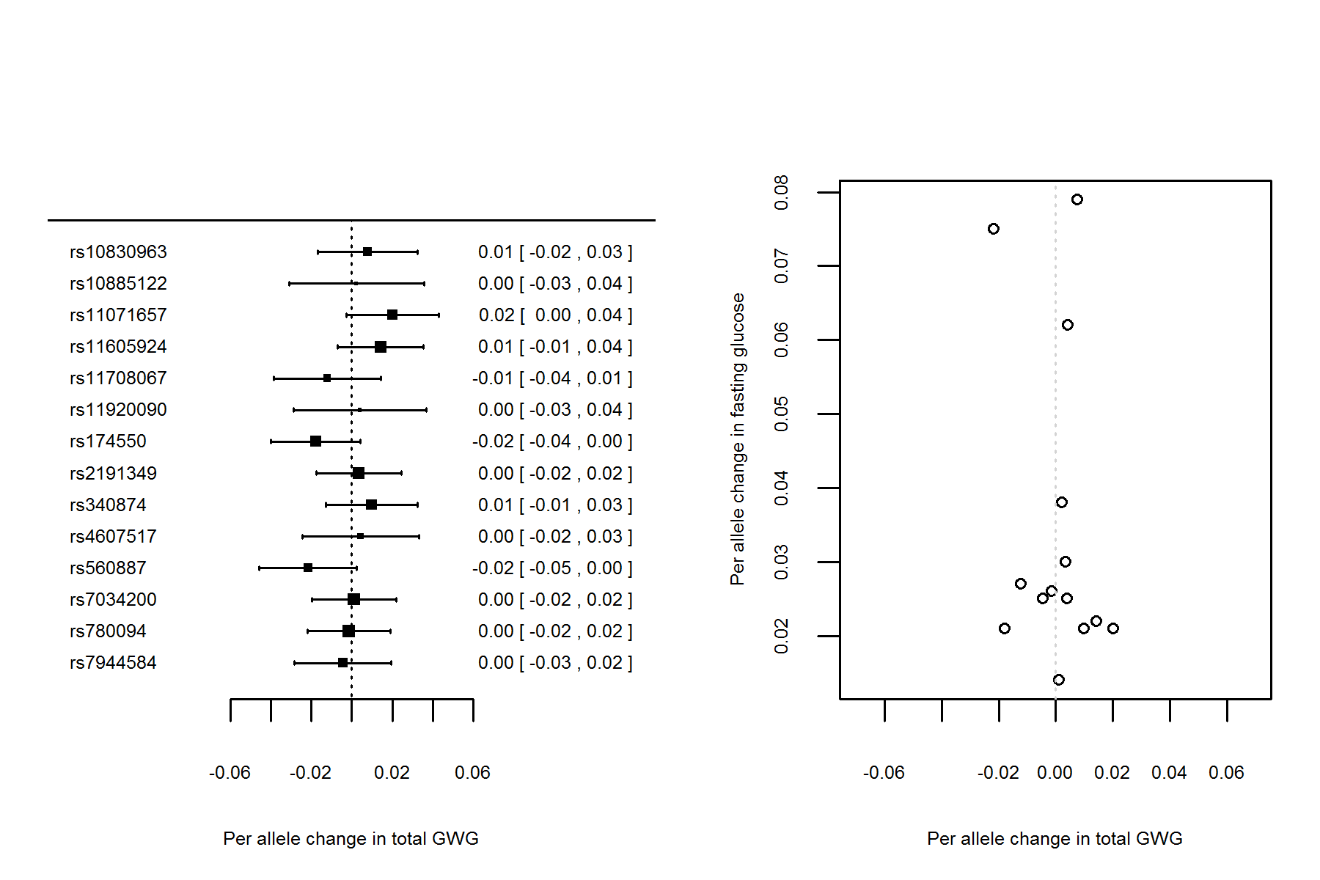** |

**eFigure 5: Summary of previously reported loci for type 2 diabetes and their effect on GWG using maternal genotype (A-C) and offspring genotype (D-F).** Plots on the left are effect sizes (with SE) on GWG of 64 known type 2 diabetes loci. Plots on the right are odds ratios (y axis) of previously reported type 2 diabetes loci[^26^](#_ENREF_26) plotted against changes in GWG z-score (x axis) per type 2 diabetes risk allele. The colour of each dot indicates the GWG association P-Value: orange, 5×10^−8^≤*P*<0.001; yellow, 0.001≤*P*<0.01; green, 0.01≤*P*<0.05 white, *P*≥0.05

| **A) Early GWG, Maternal genotype**  **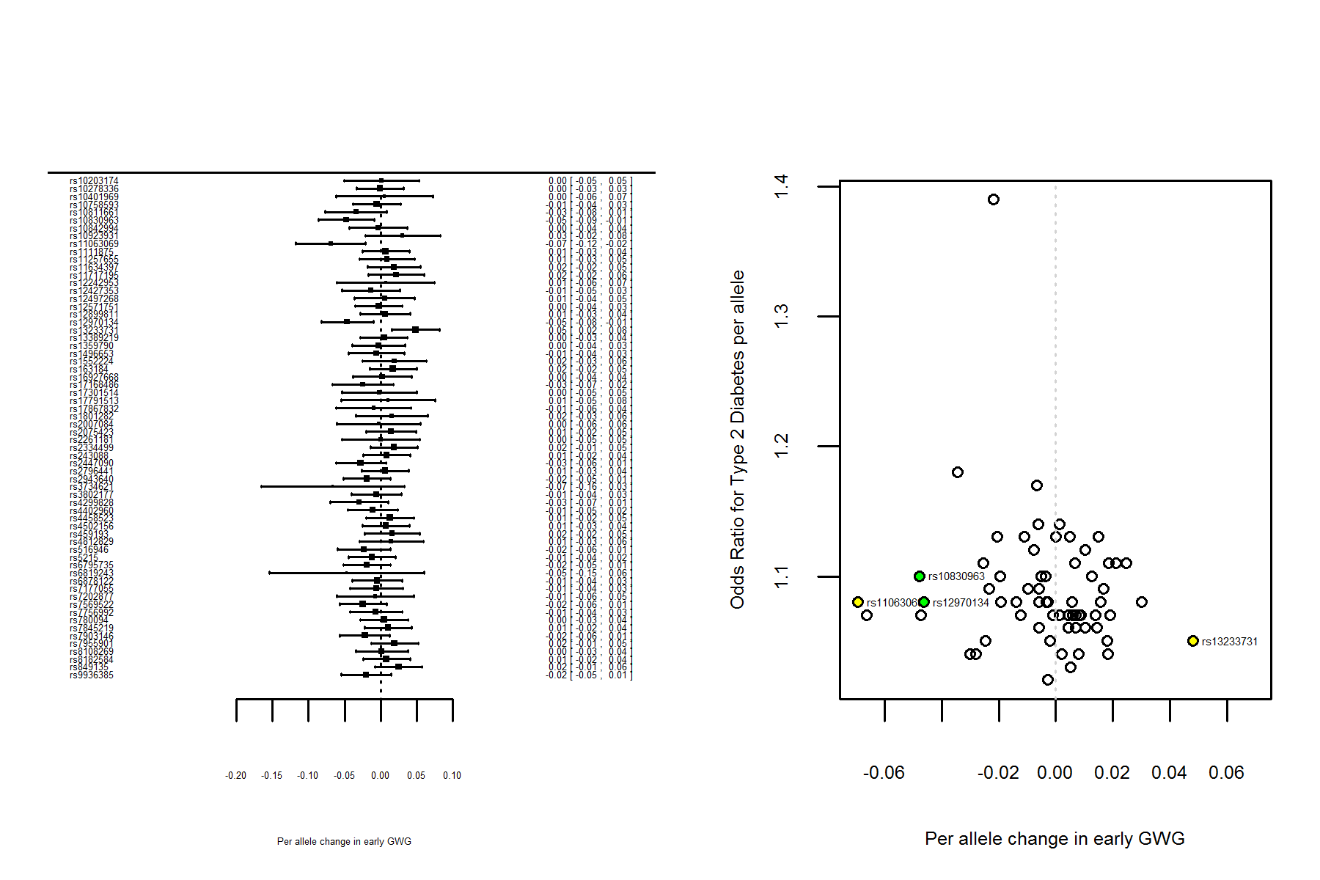** |
| --- |
| **B) Late GWG, Maternal Genotype**  **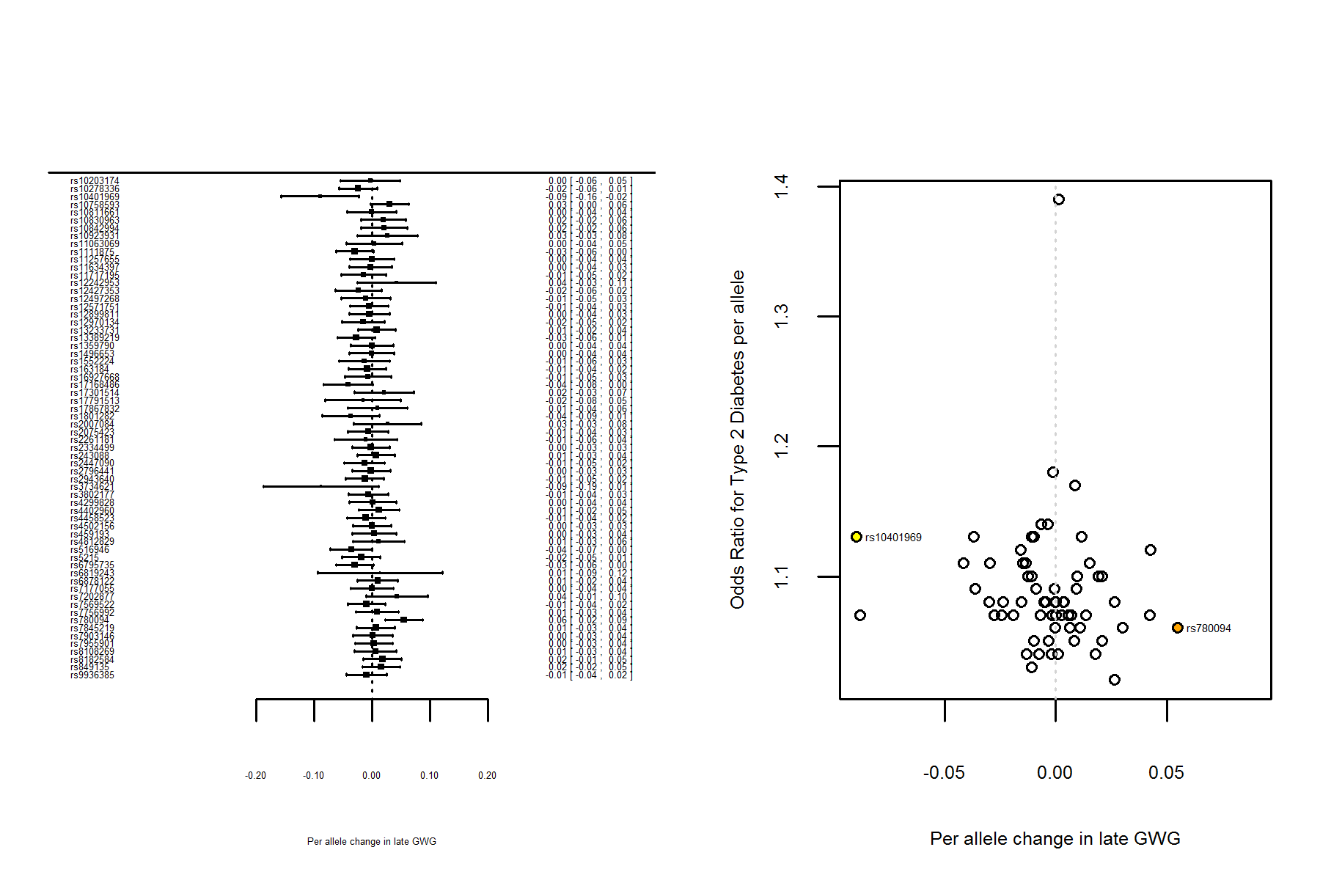** |

| **C) Total GWG, Maternal genotype**  **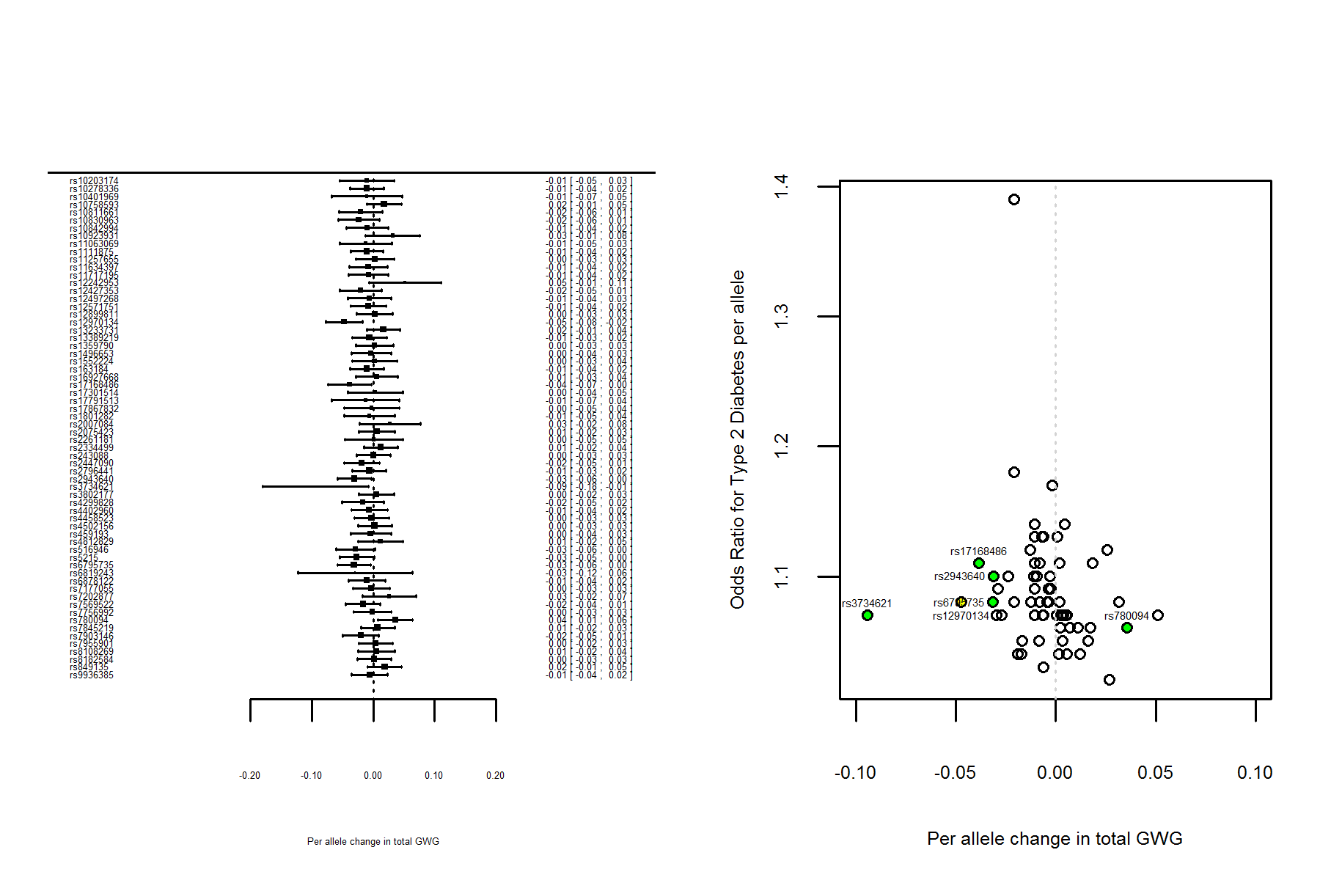** |
| --- |
| **D) Early GWG, Offspring genotype**  **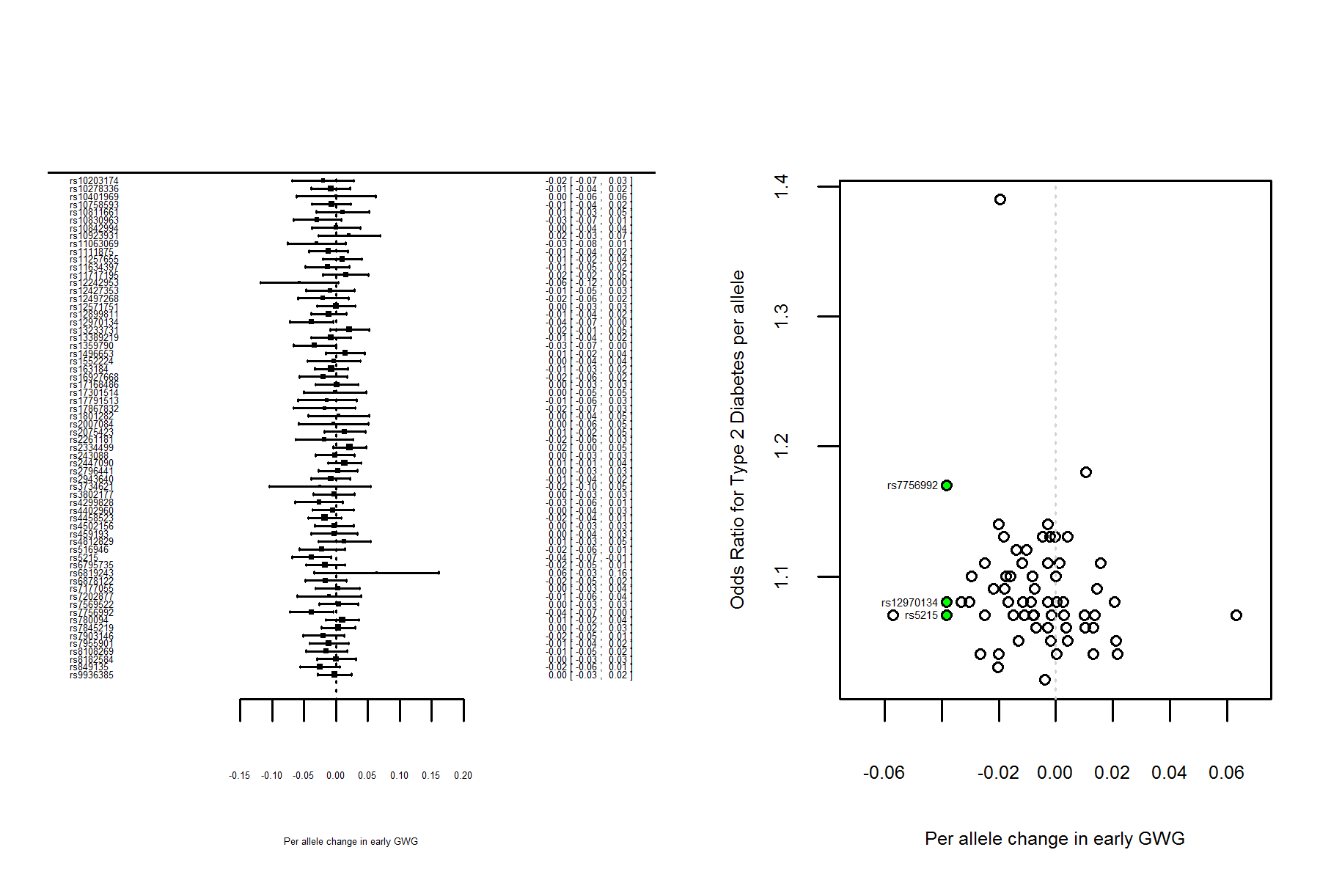** |

| **E) Late GWG, Offspring genotype**  **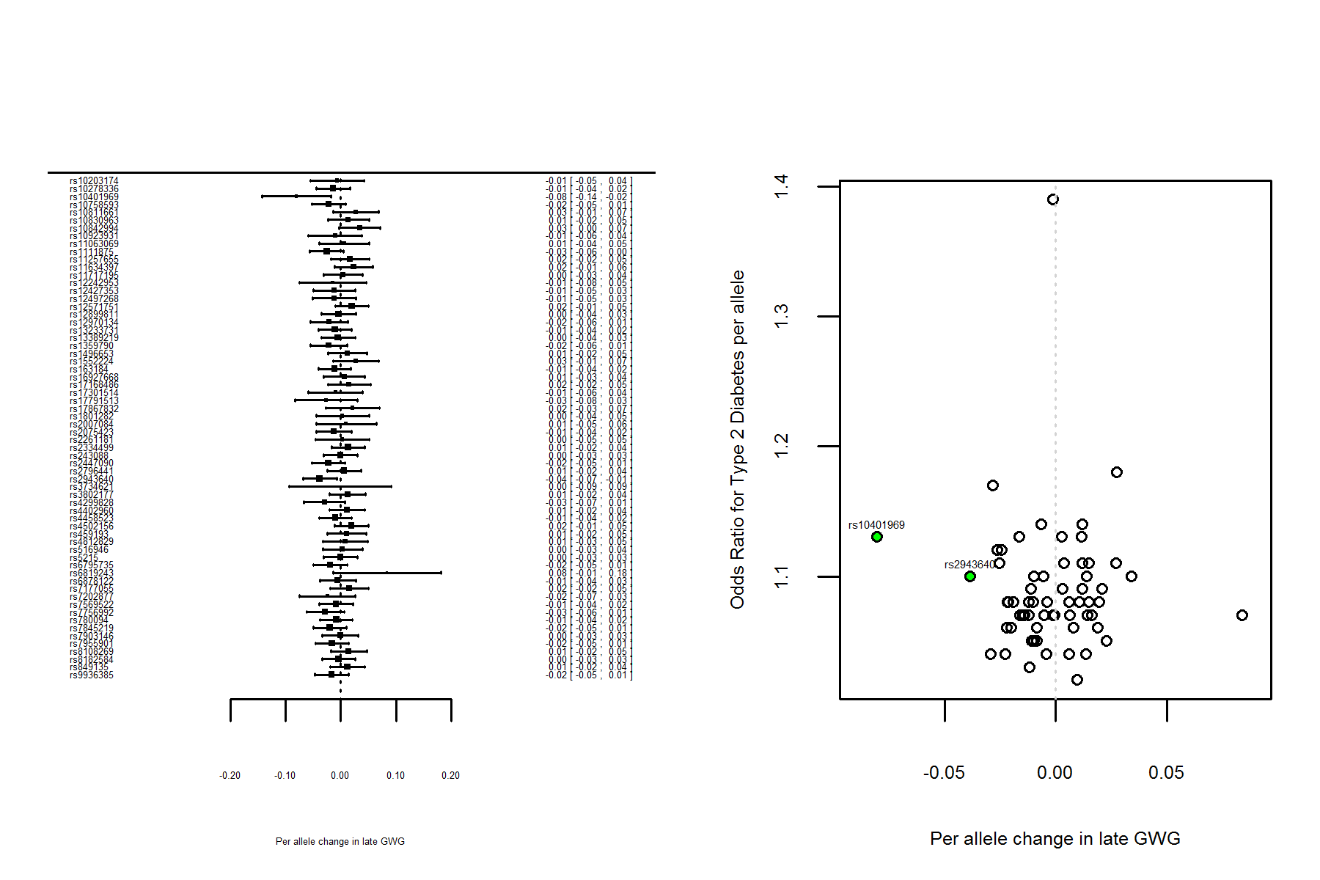** |
| --- |
| **F) Total GWG, Offspring genotype**  **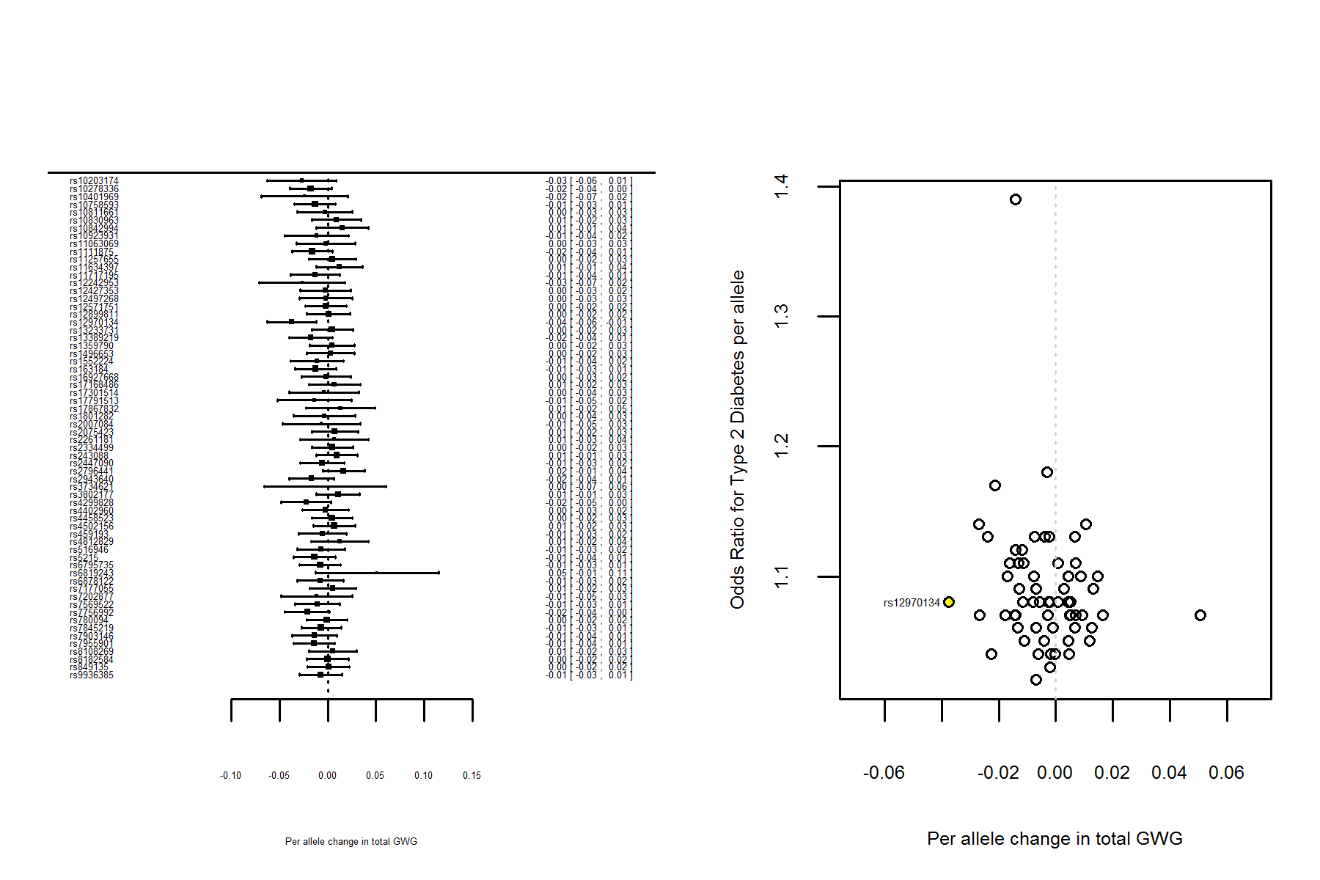** |

**eFigure 6: Summary of previously reported loci for birthweight and their effect on GWG using maternal genotype (A-C) and offspring genotype (D-F).** Plots on the left are effect sizes (with SE) on GWG of 7 known birth weight loci. Plots on the right are effect sizes (y axis) of previously reported birth weight[^19^](#_ENREF_19) loci plotted against changes in GWG z-score (x axis) per birth weight raising allele. The colour of each dot indicates the GWG association P-Value: orange, 5×10^−8^≤*P*<0.001; yellow, 0.001≤*P*<0.01; green, 0.01≤*P*<0.05 white, *P*≥0.05

| **A) Early GWG, Maternal genotype**  **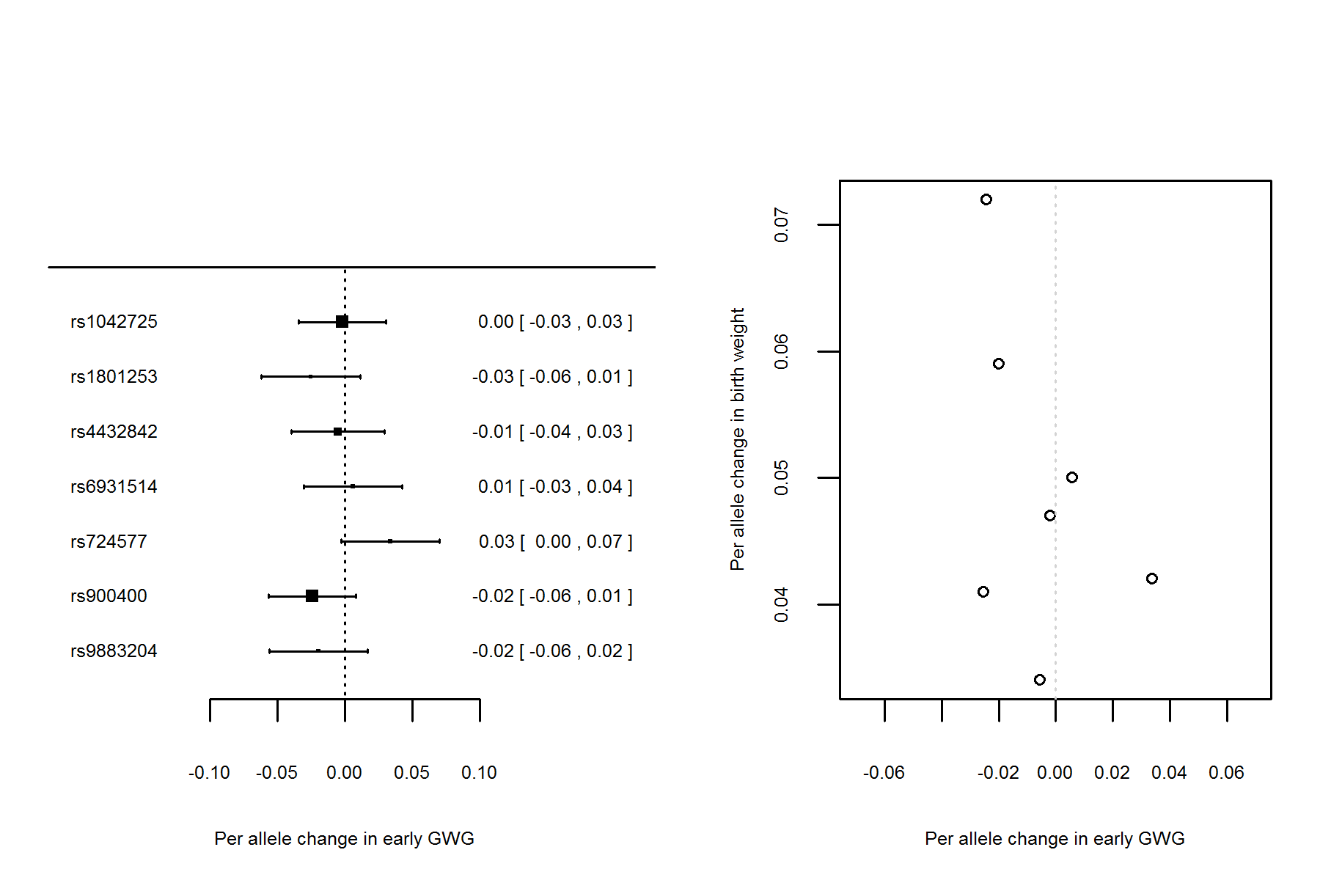** |
| --- |
| **B) Late GWG, Maternal Genotype**  **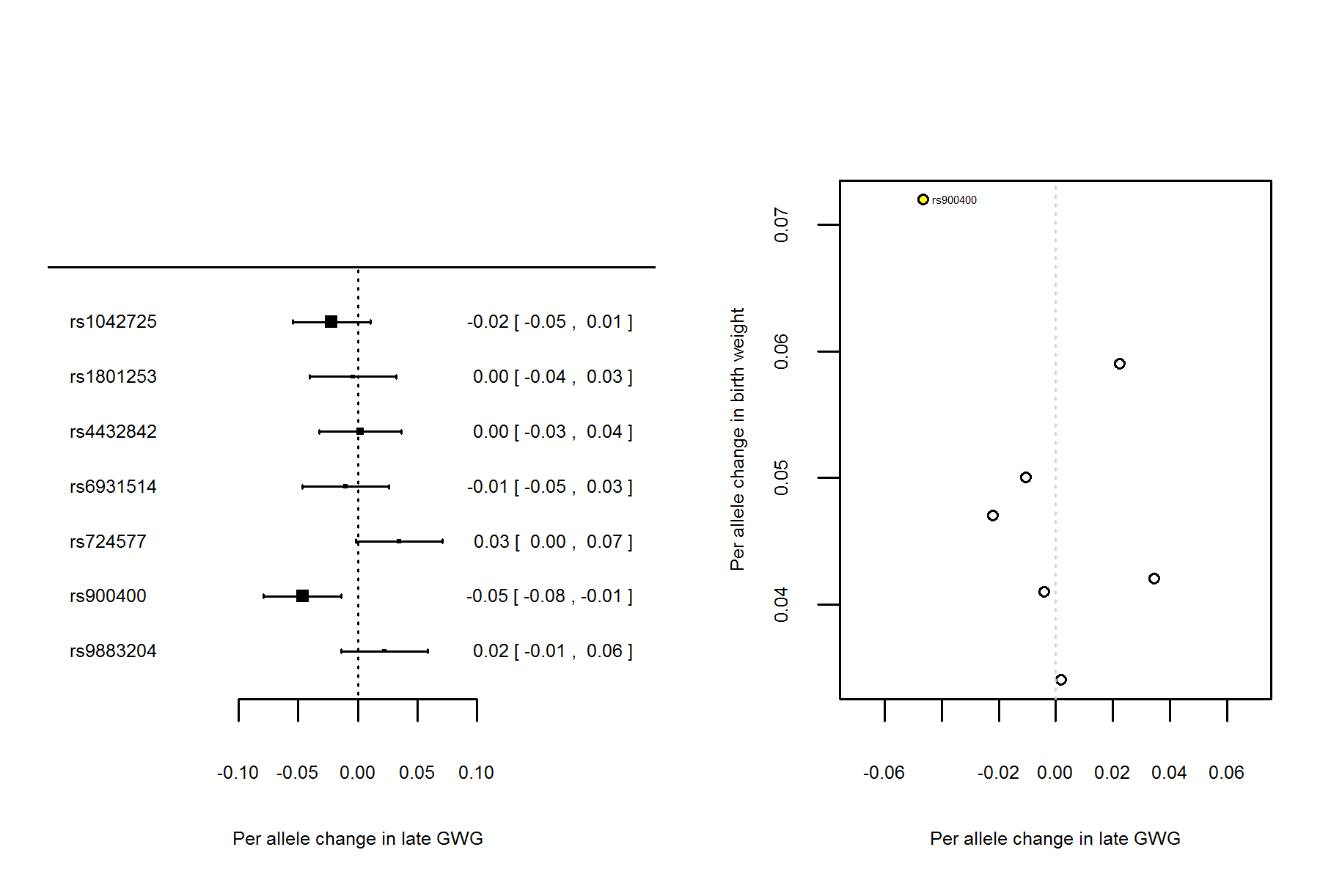** |

| **C) Total GWG, Maternal genotype**  **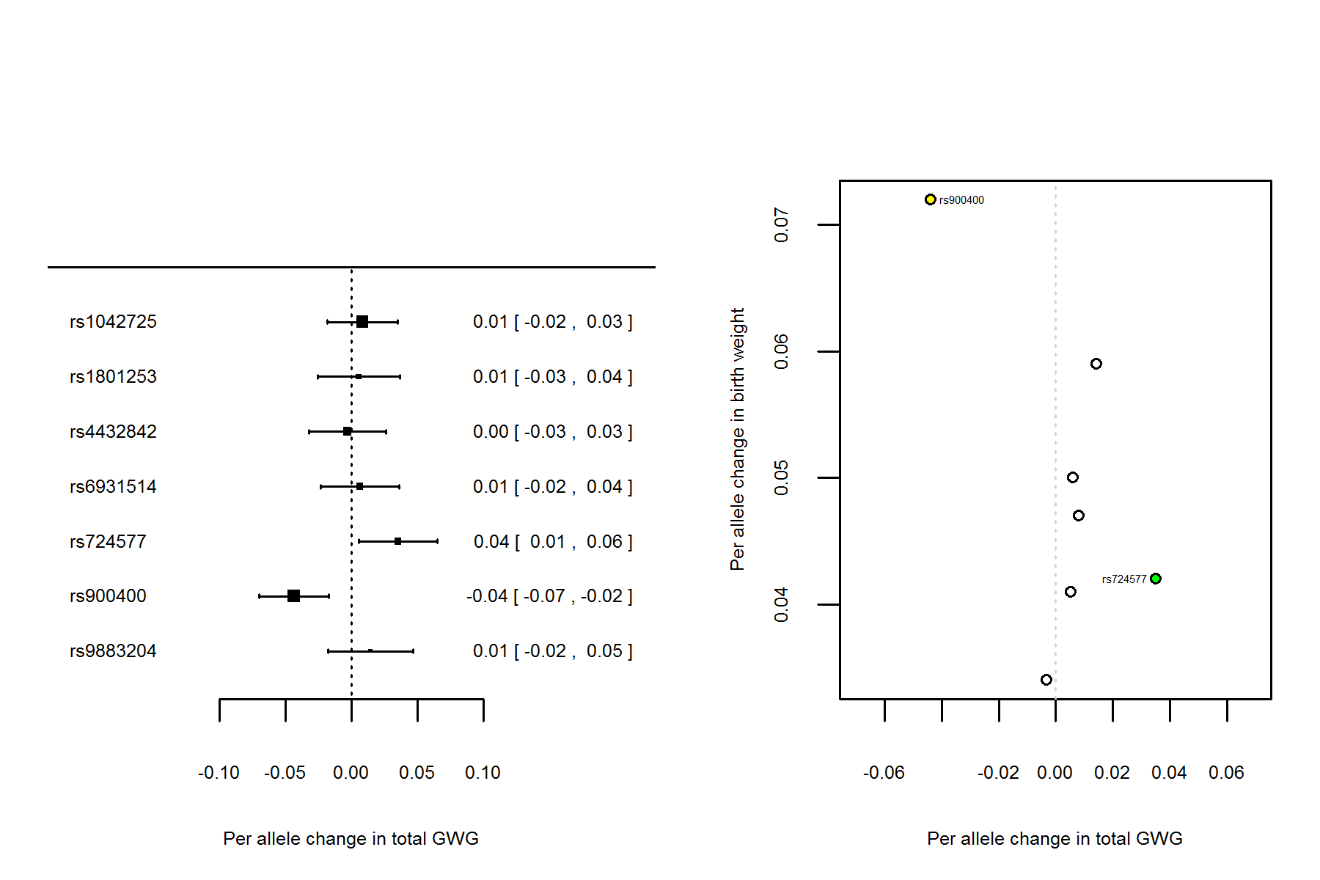** |
| --- |
| **D) Early GWG, Offspring genotype**  **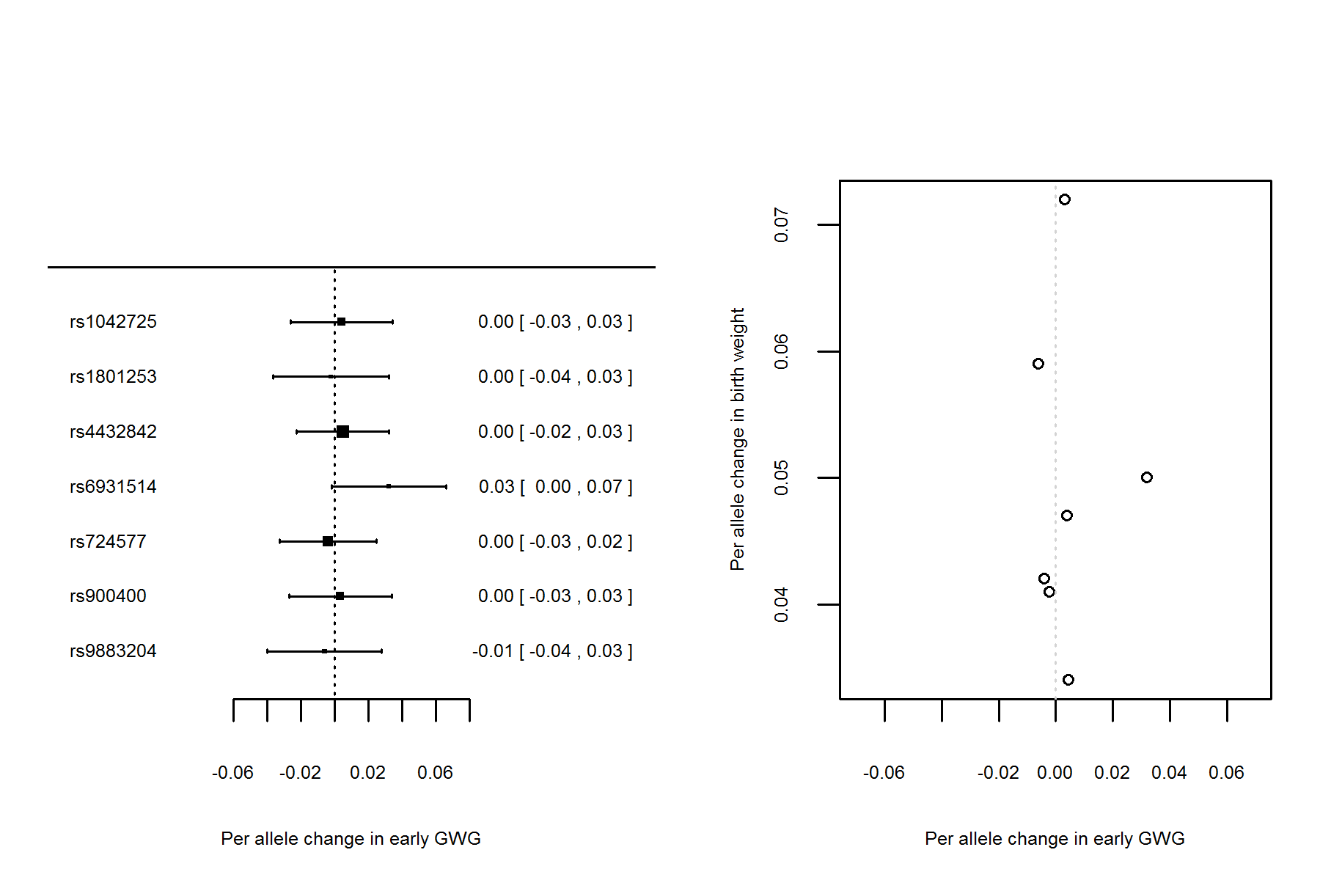** |

| **E) Late GWG, Offspring genotype**  **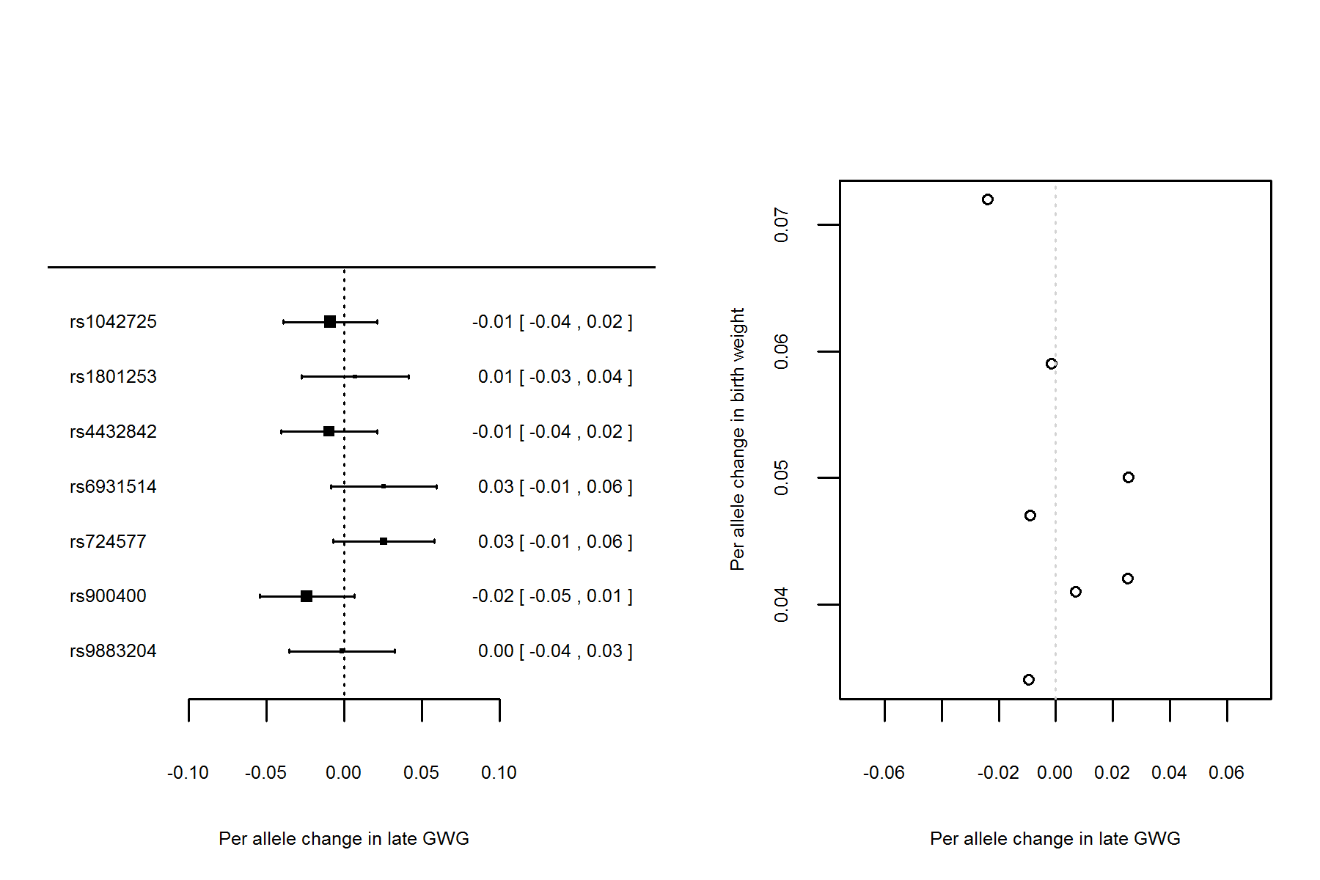** |
| --- |
| **F) Total GWG, Offspring genotype**  **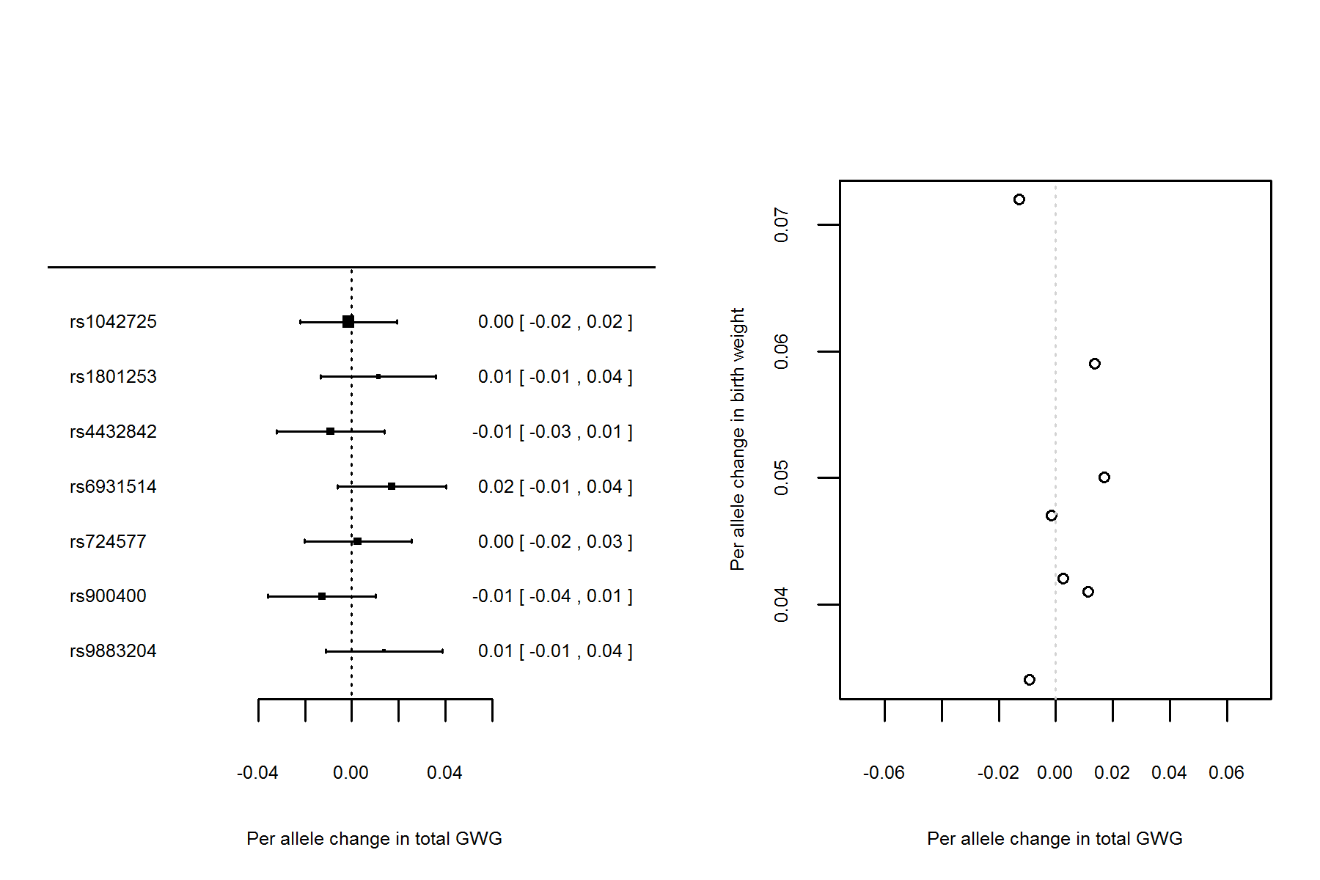** |

**eFigure 7: Summary of previously reported loci for blood pressure and their effect on GWG using maternal genotype (A-C) and offspring genotype (D-F).** Plots on the left are effect sizes (with SE) on GWG of 25 known blood pressure loci. Plots on the right are effect sizes (y axis) of previously reported blood pressure loci[^23^](#_ENREF_23)^,^[^24^](#_ENREF_24) plotted against changes in GWG z-score (x axis) per blood pressure raising allele. The colour of each dot indicates the GWG association P-Value: orange, 5×10^−8^≤*P*<0.001; yellow, 0.001≤*P*<0.01; green, 0.01≤*P*<0.05 white, *P*≥0.05

| **A) Early GWG, Maternal genotype**  **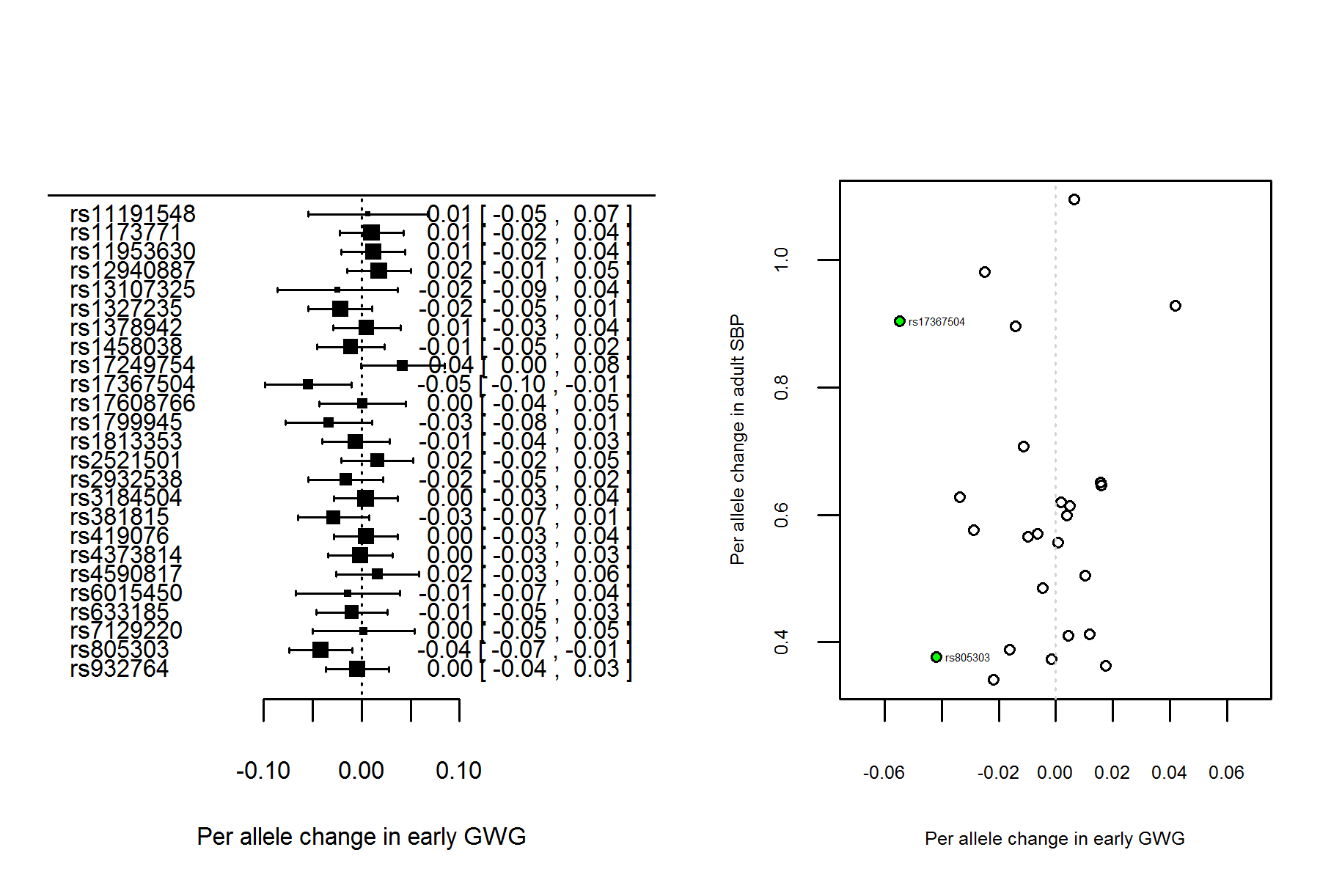** |
| --- |
| **B) Late GWG, Maternal Genotype**  **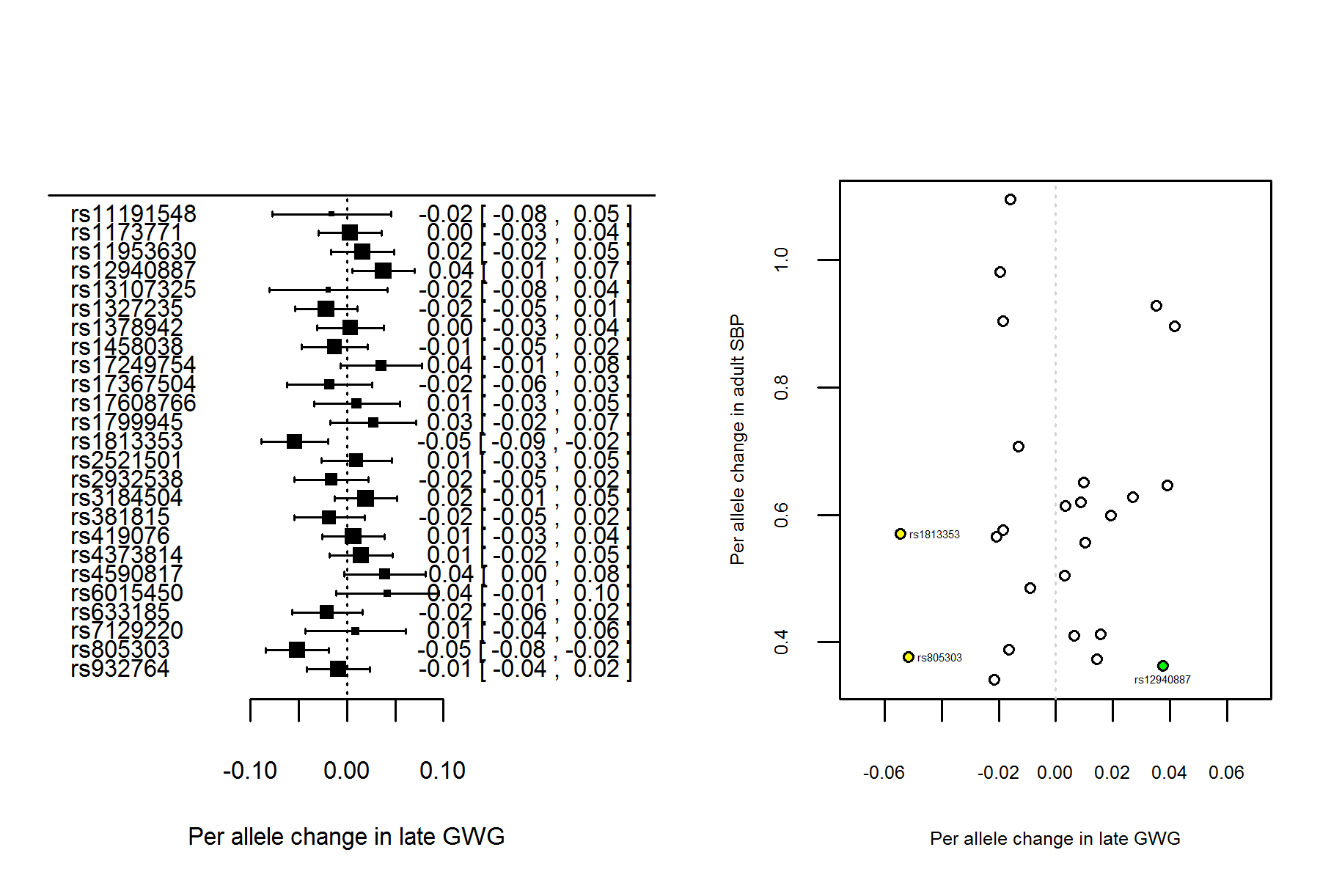** |

| **C) Total GWG, Maternal genotype**  **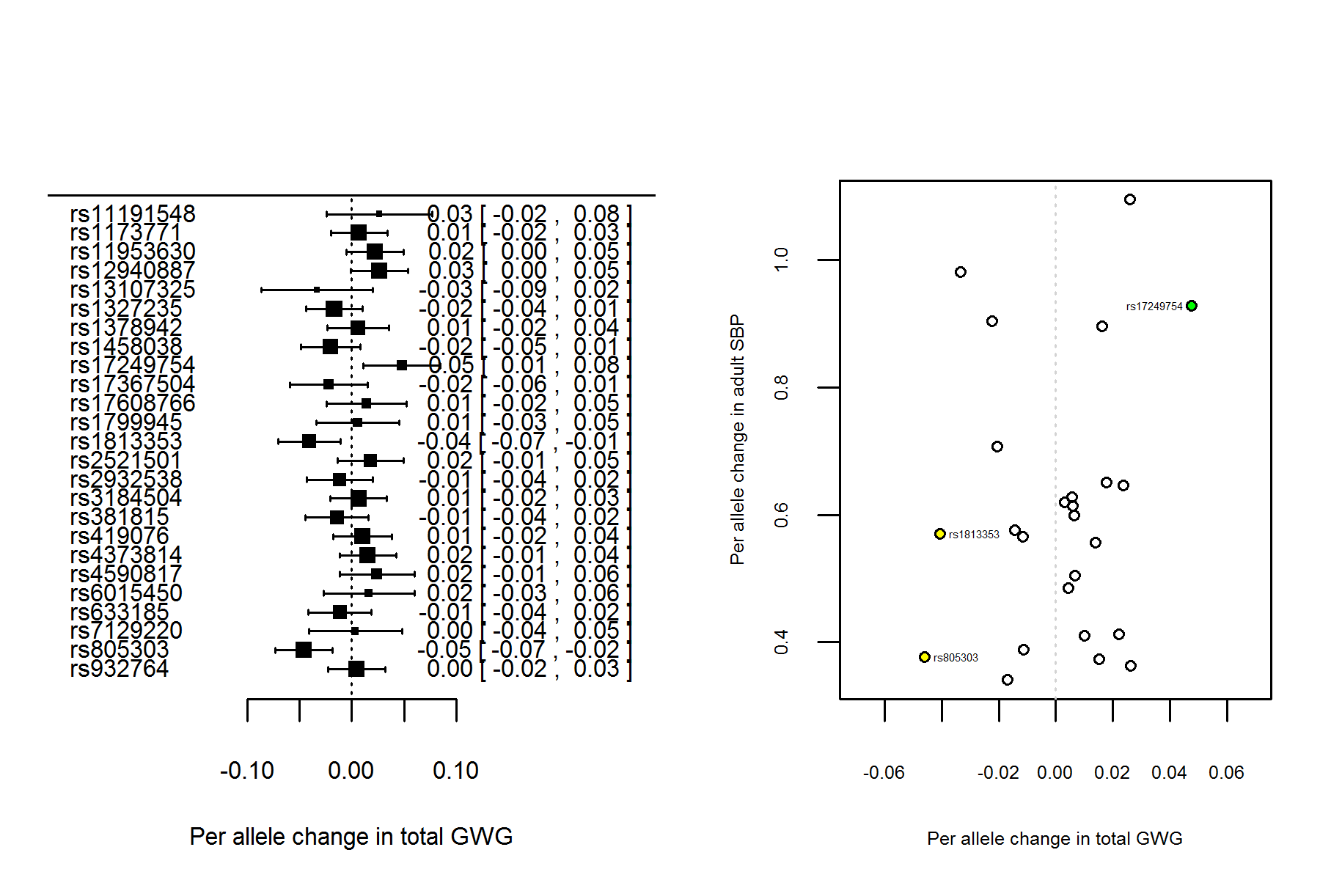** |
| --- |
| **D) Early GWG, Offspring genotype**  **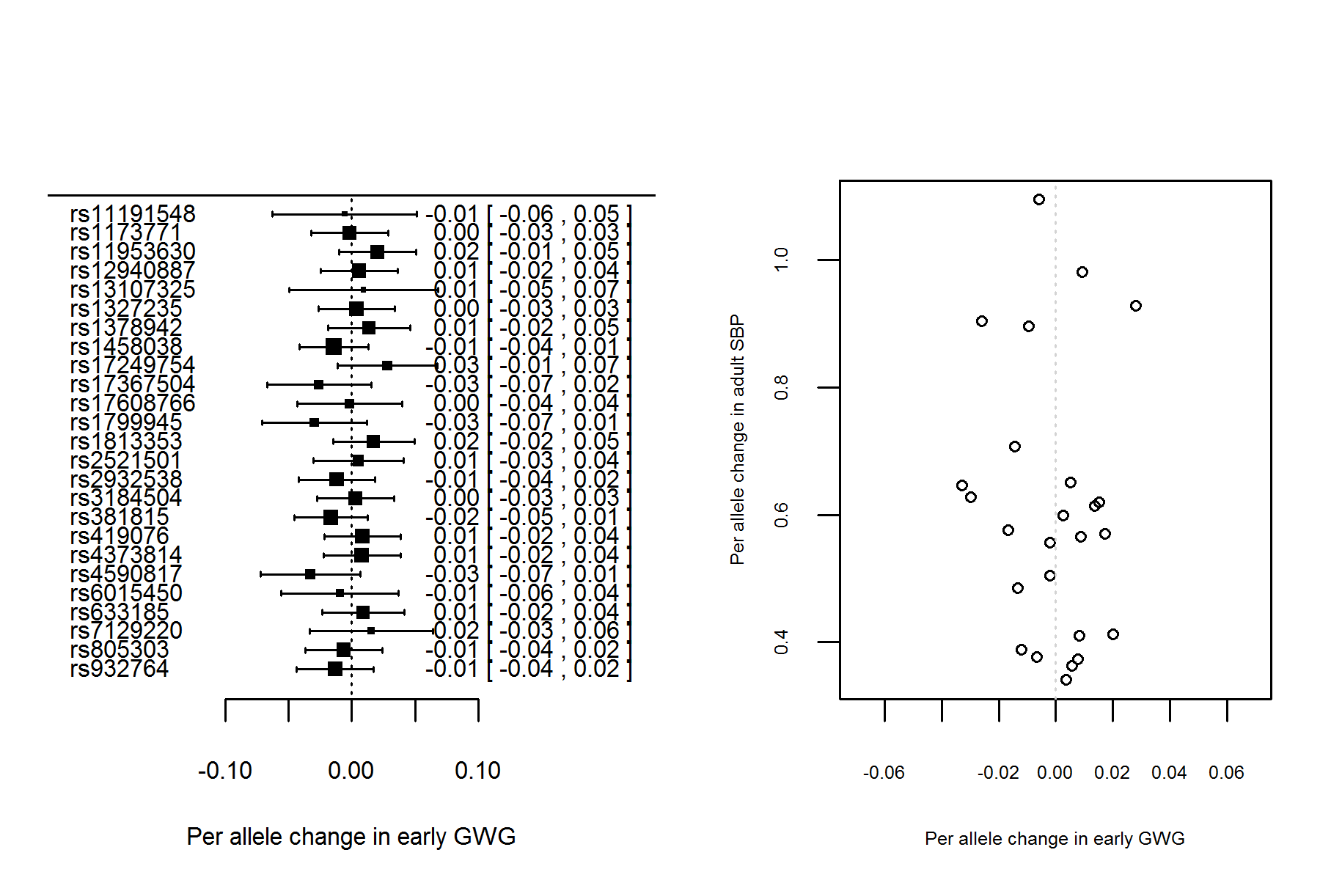** |

| **E) Late GWG, Offspring genotype**  **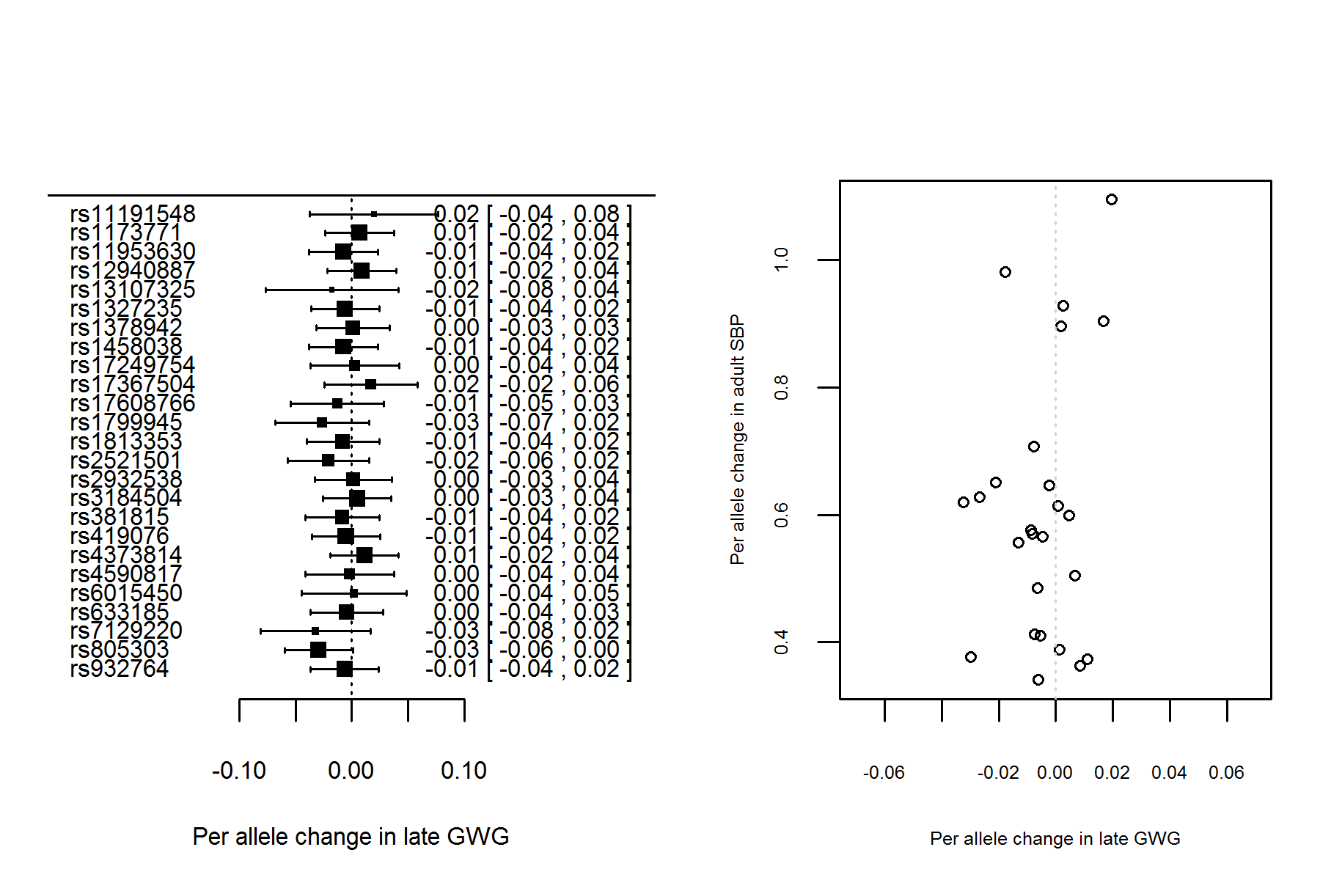** |
| --- |
| **F) Total GWG, Offspring genotype**  **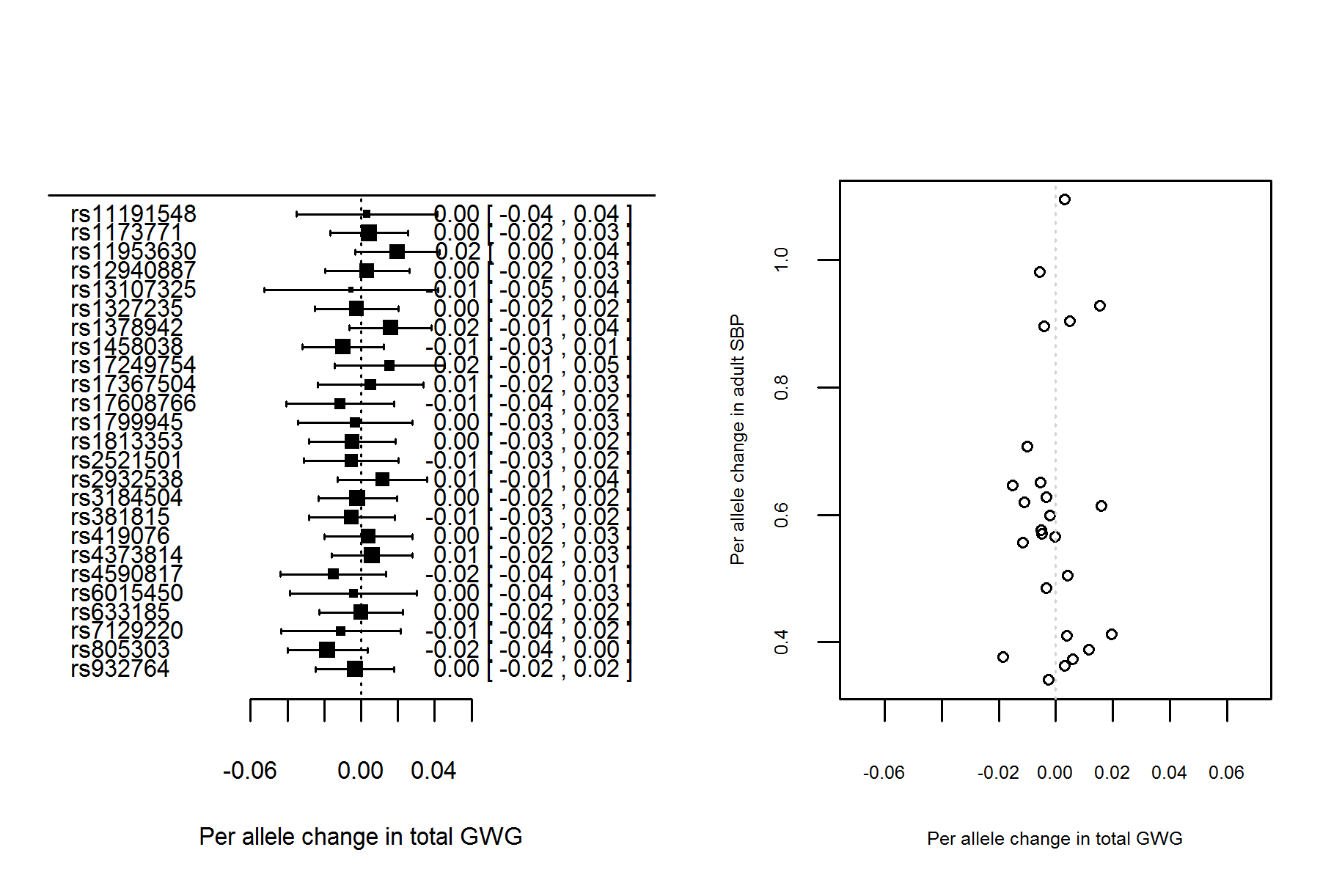** |

**eFigure 8: Summary of previously reported loci for waist-hip ratio and their effect on GWG using maternal genotype (A-C) and offspring genotype (D-F).** Plots on the left are effect sizes (with SE) on GWG of 14 known waist-hip ratio loci.[^21^](#_ENREF_21) Plots on the right are effect sizes (y axis) of previously reported waist-hip ratio loci plotted against changes in GWG z-score (x axis) per waist-hip ratio raising allele. The colour of each dot indicates the GWG association P-Value: orange, 5×10^−8^≤*P*<0.001; yellow, 0.001≤*P*<0.01; green, 0.01≤*P*<0.05 white, *P*≥0.05

| **A) Early GWG, Maternal genotype**  **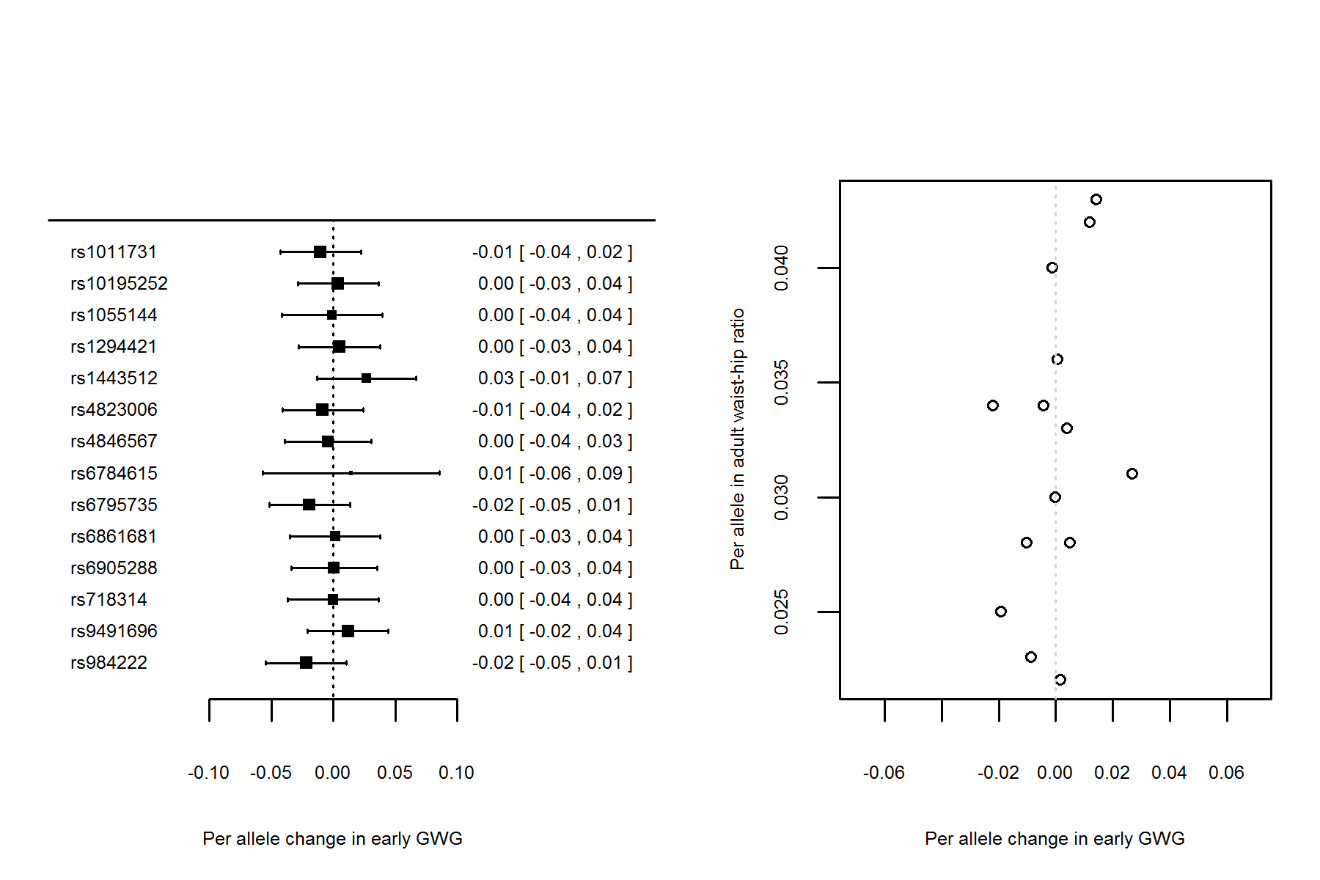** |
| --- |
| **B) Late GWG, Maternal Genotype**  **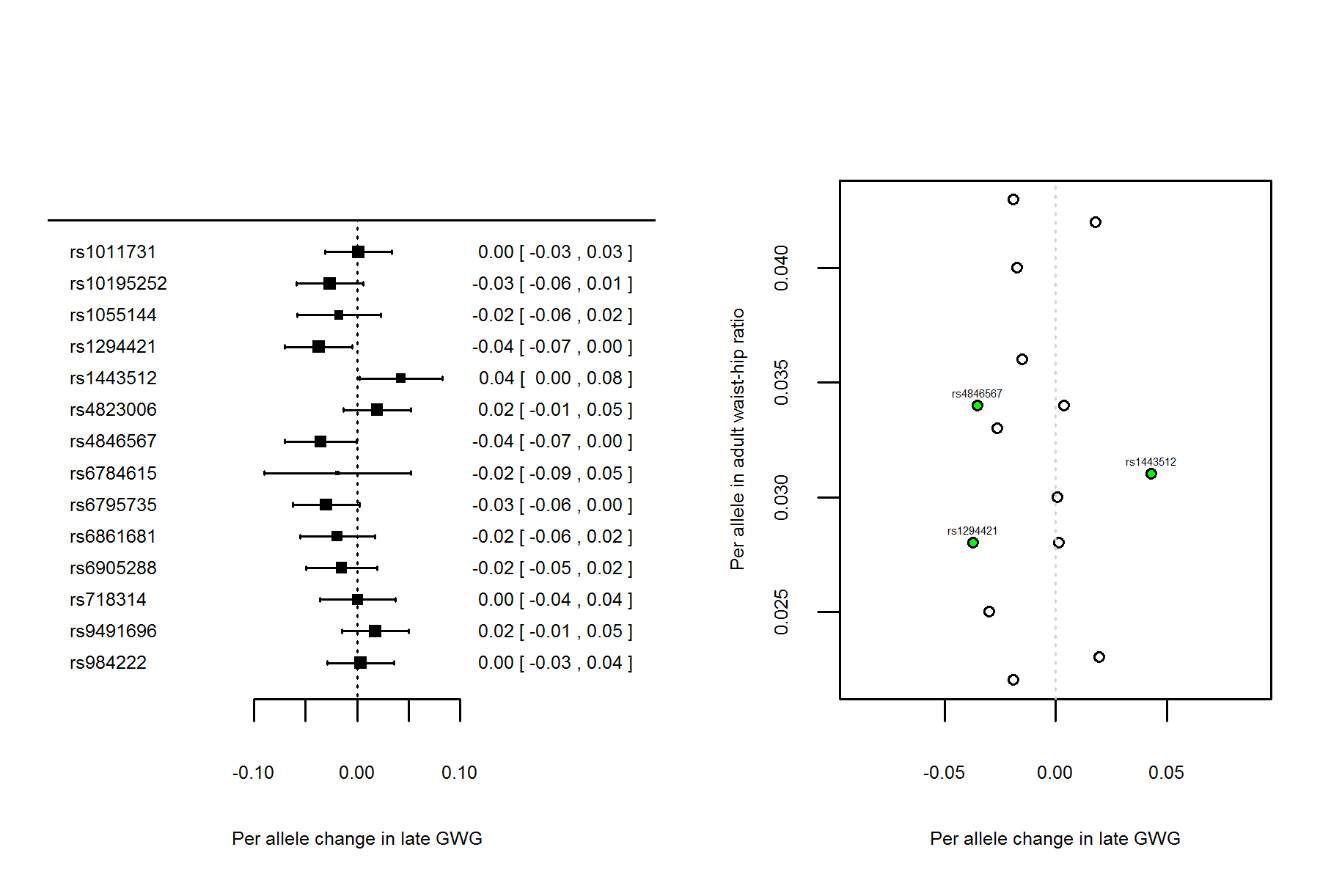** |

| **C) Total GWG, Maternal genotype**  **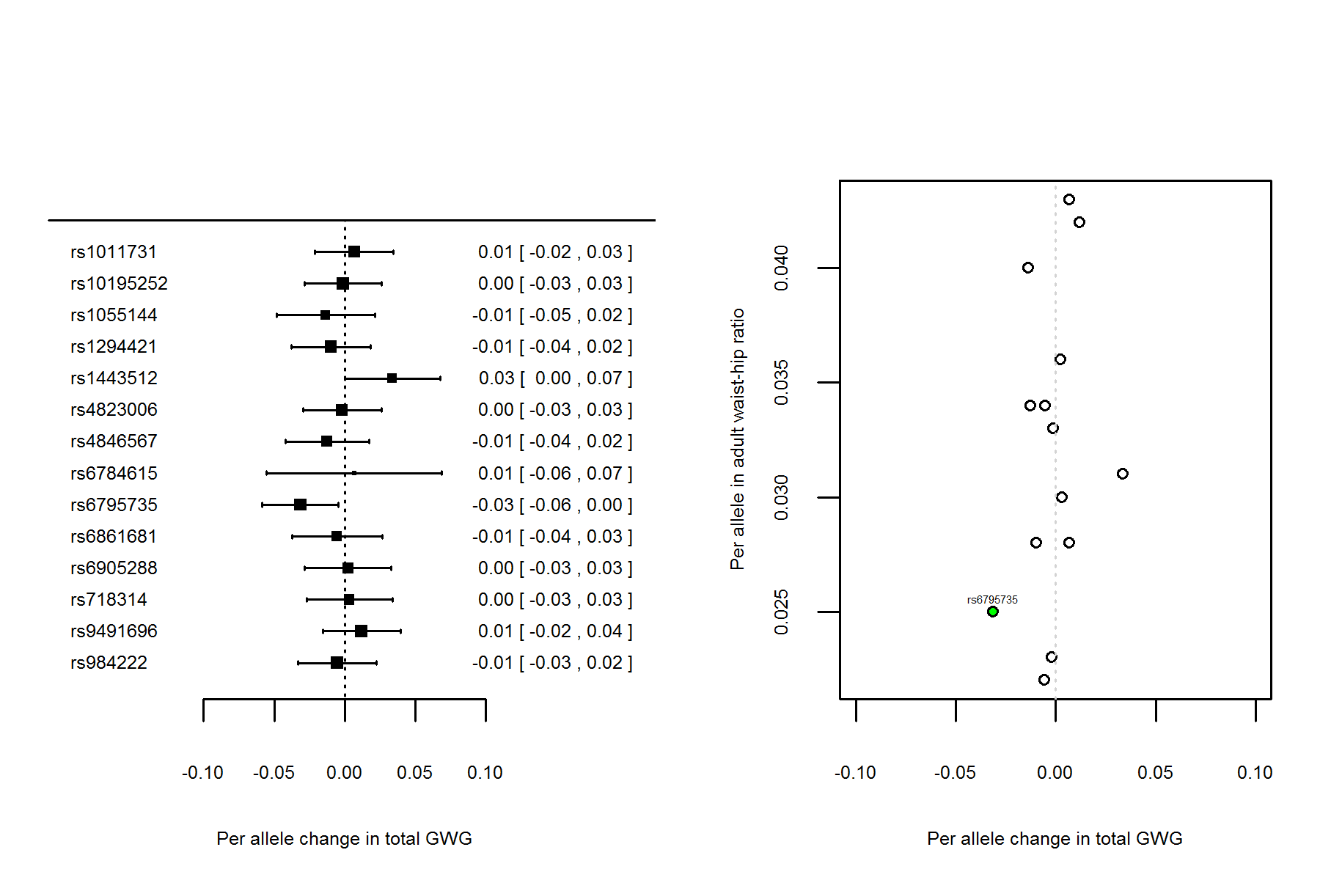** |
| --- |
| **D) Early GWG, Offspring genotype**  **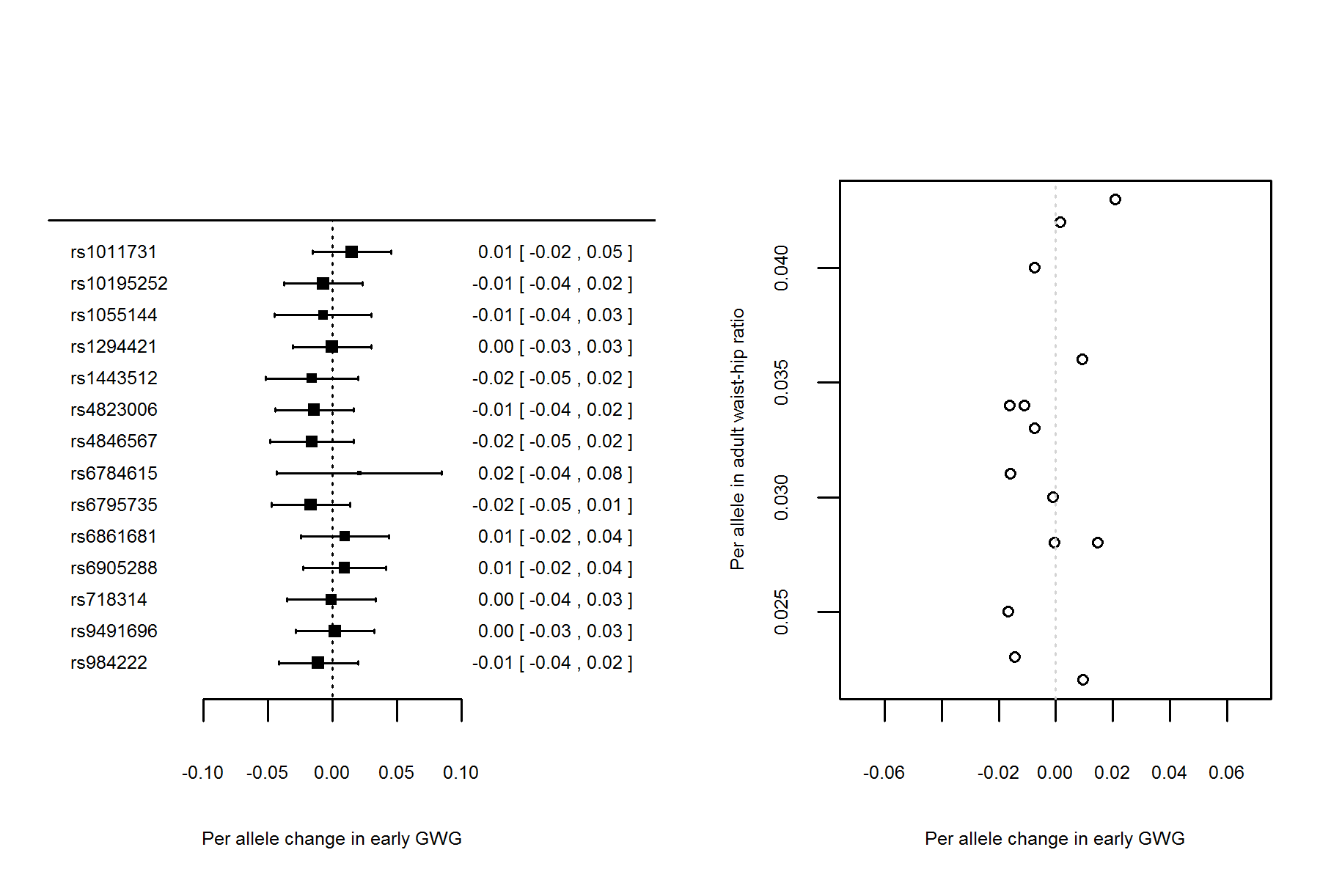** |

| **E) Late GWG, Offspring genotype**  **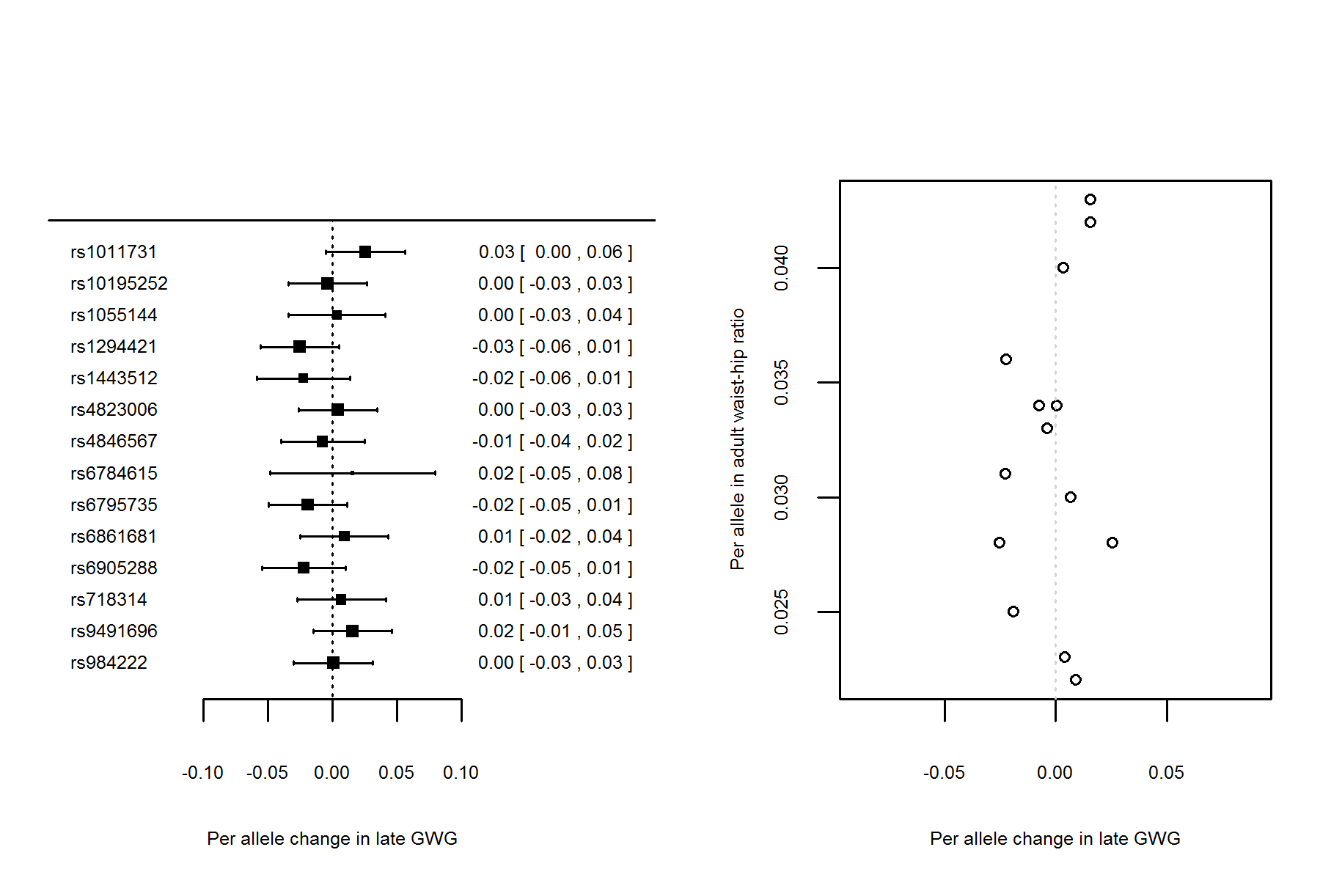** |
| --- |
| **F) Total GWG, Offspring genotype**  **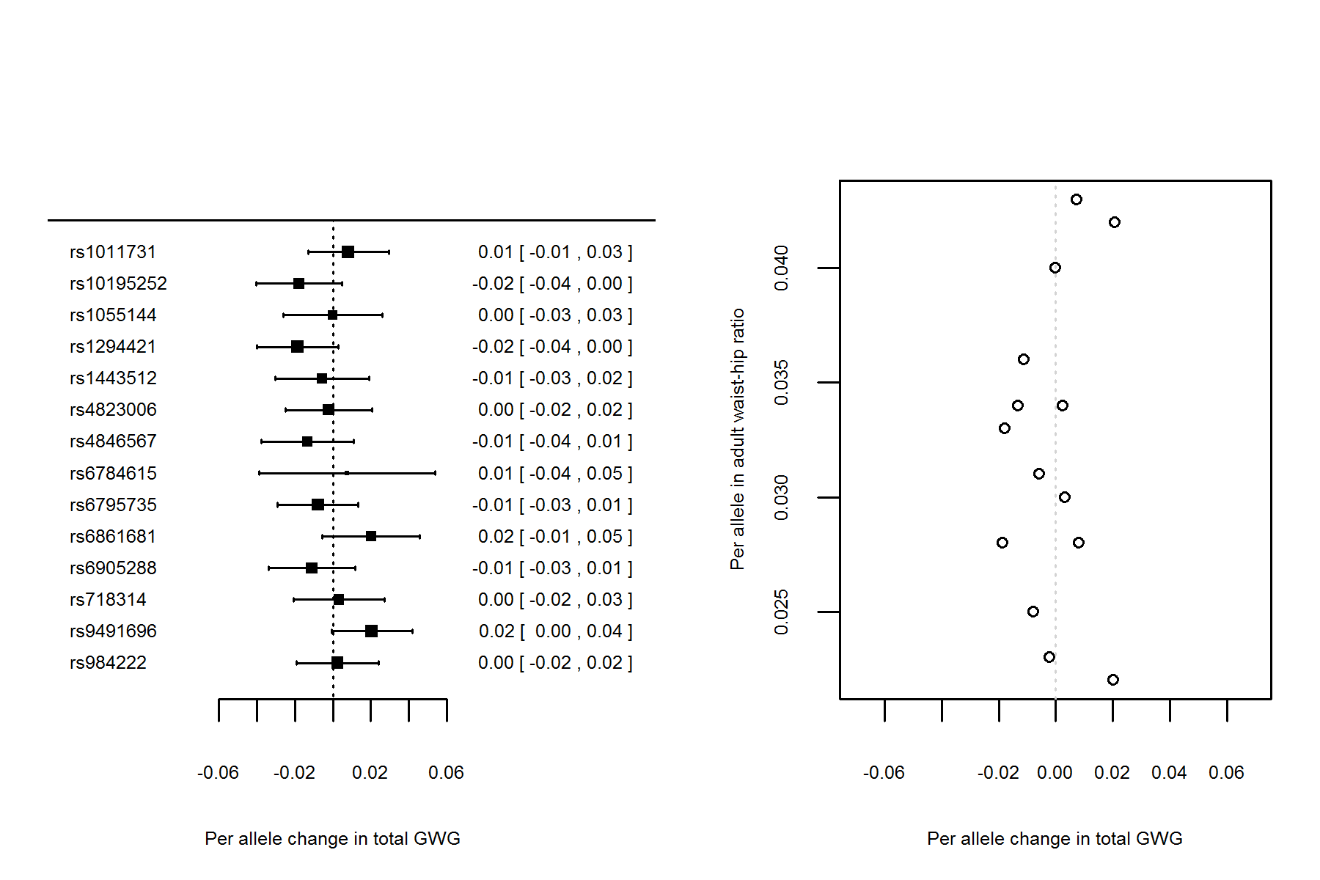** |

**eFigure 9: Summary of previously reported loci for vitamin D (25(OH)D) and their effect on GWG using maternal genotype and offspring genotype.** The plot are effect sizes (with SE) on early, late or total GWG using the maternal or offspring genotype for 2 known vitamin D loci.[^27^](#_ENREF_27) As there are just two vitamin D loci we have not shown Volcano plots for overlap of genetic variants for this trait with GWG.

**
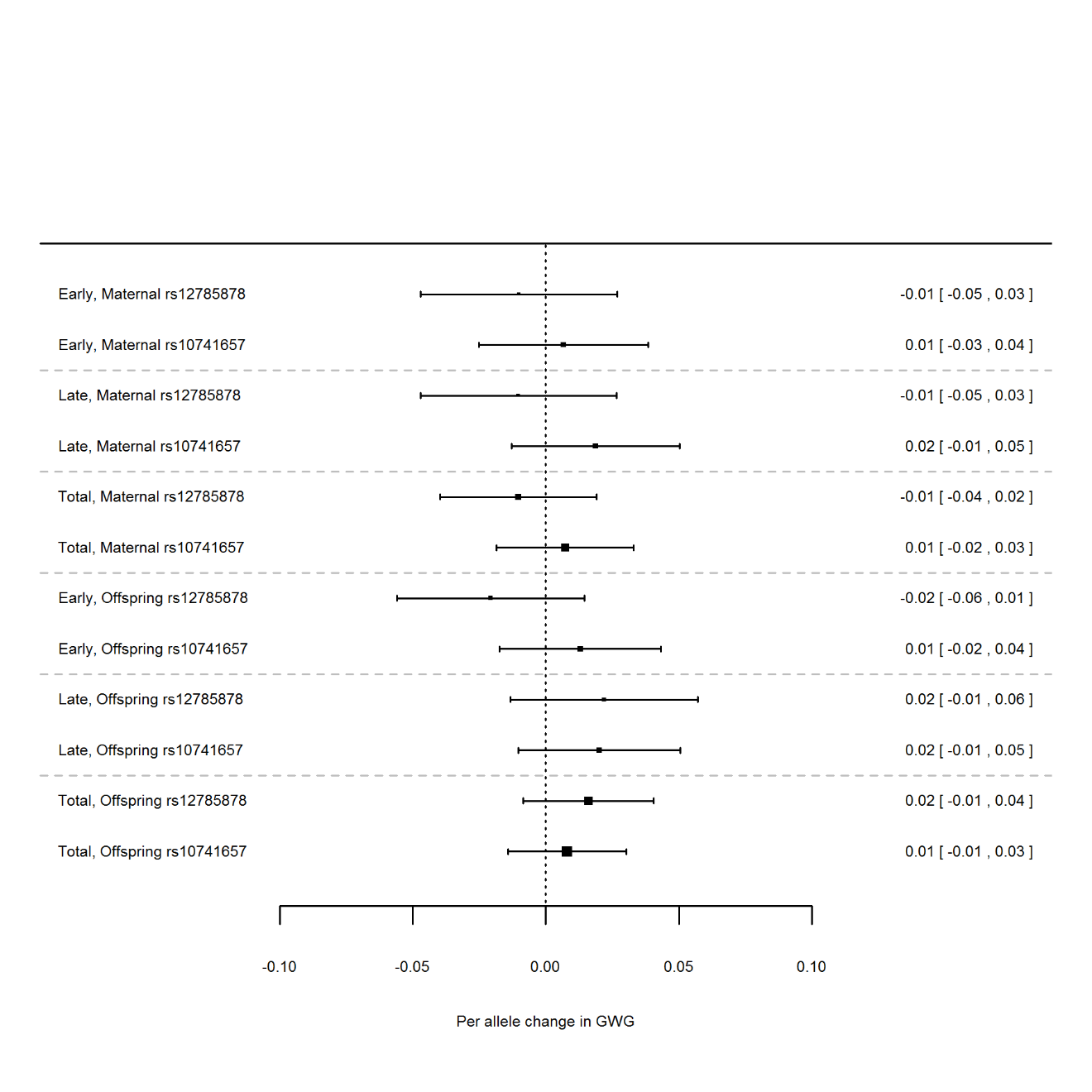
**

**eFigure 10: Summary of previously reported loci for height and their effect on GWG using maternal genotype (A-C) and offspring genotype (D-F).** Plots are effect sizes (y axis) of 180 previously reported height loci[^22^](#_ENREF_22) plotted against changes in GWG z-score (x axis) per height raising allele. The colour of each dot indicates the GWG association P-Value: orange, 5×10^−8^≤*P*<0.001; yellow, 0.001≤*P*<0.01; green, 0.01≤*P*<0.05 white, *P*≥0.05. Because of the very large number of loci for height we have not shown the Forrest plots of effect sizes (with SE) on GWG of the height loci.

| **A) Early GWG, Maternal genotype**  **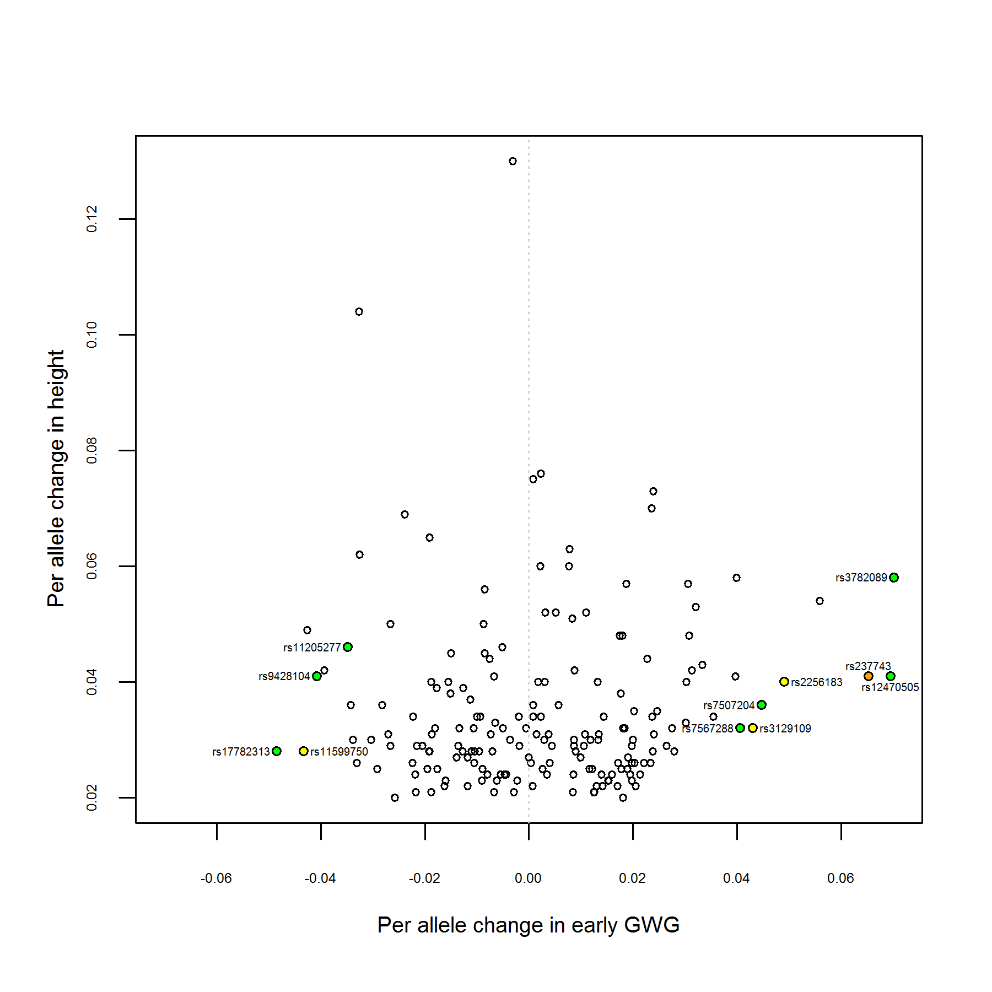** |
| --- |
| **B) Late GWG, Maternal Genotype**  **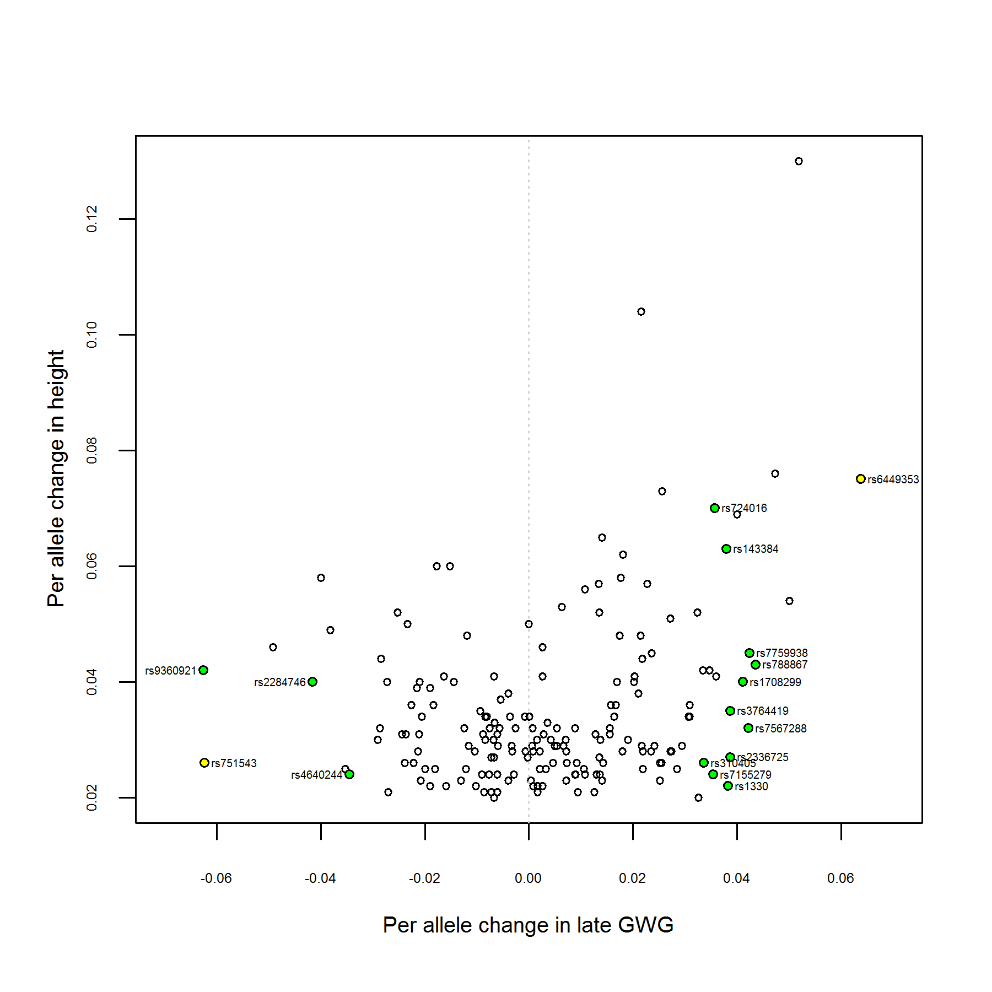** |
| **C) Total GWG, Maternal genotype**  **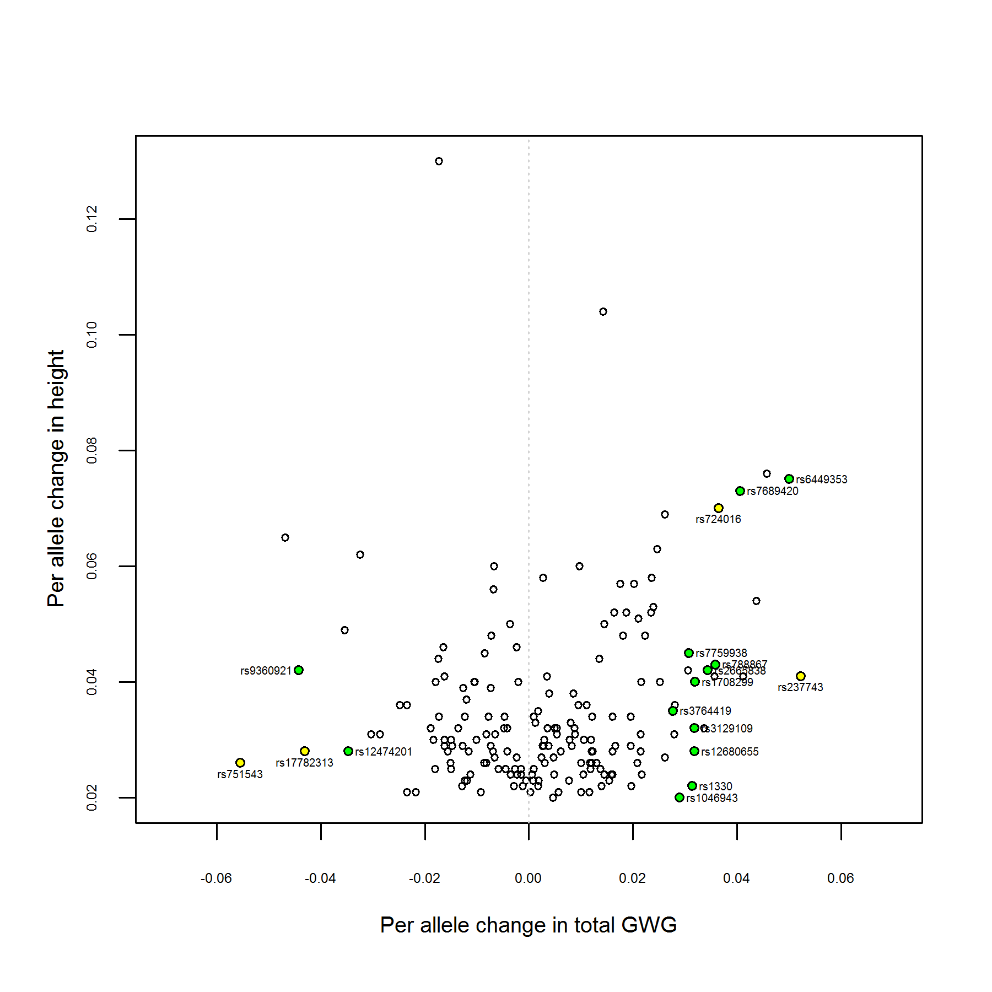** |
| **D) Early GWG, Offspring genotype**  **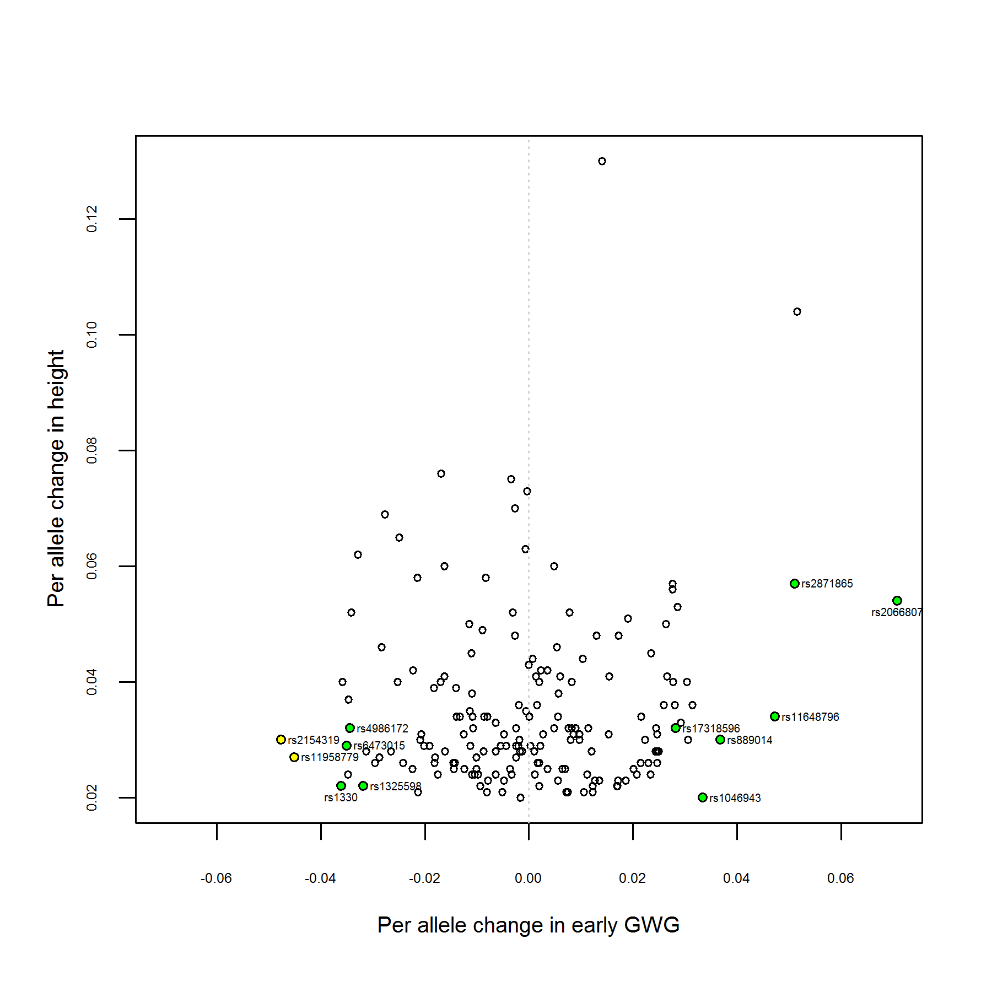** |

| **E) Late GWG, Offspring genotype**  **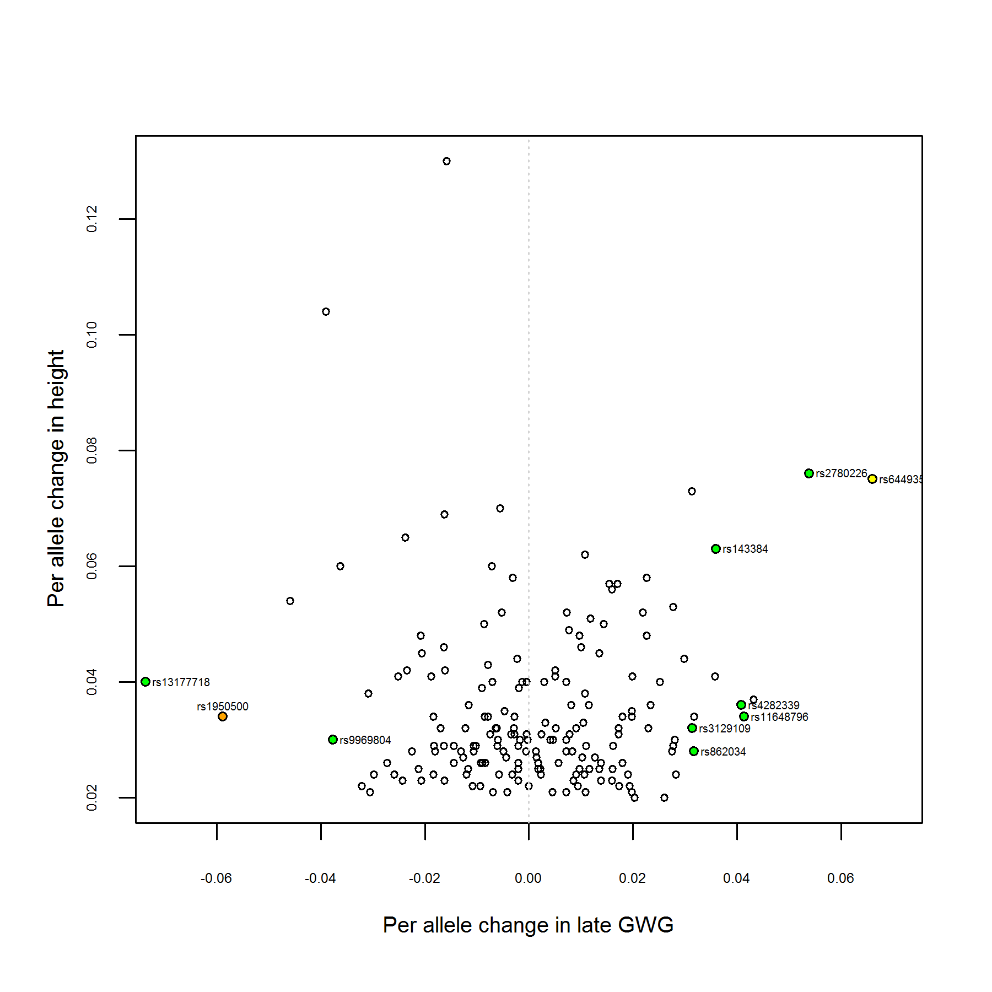** |
| --- |
| **F) Total GWG, Offspring genotype**  **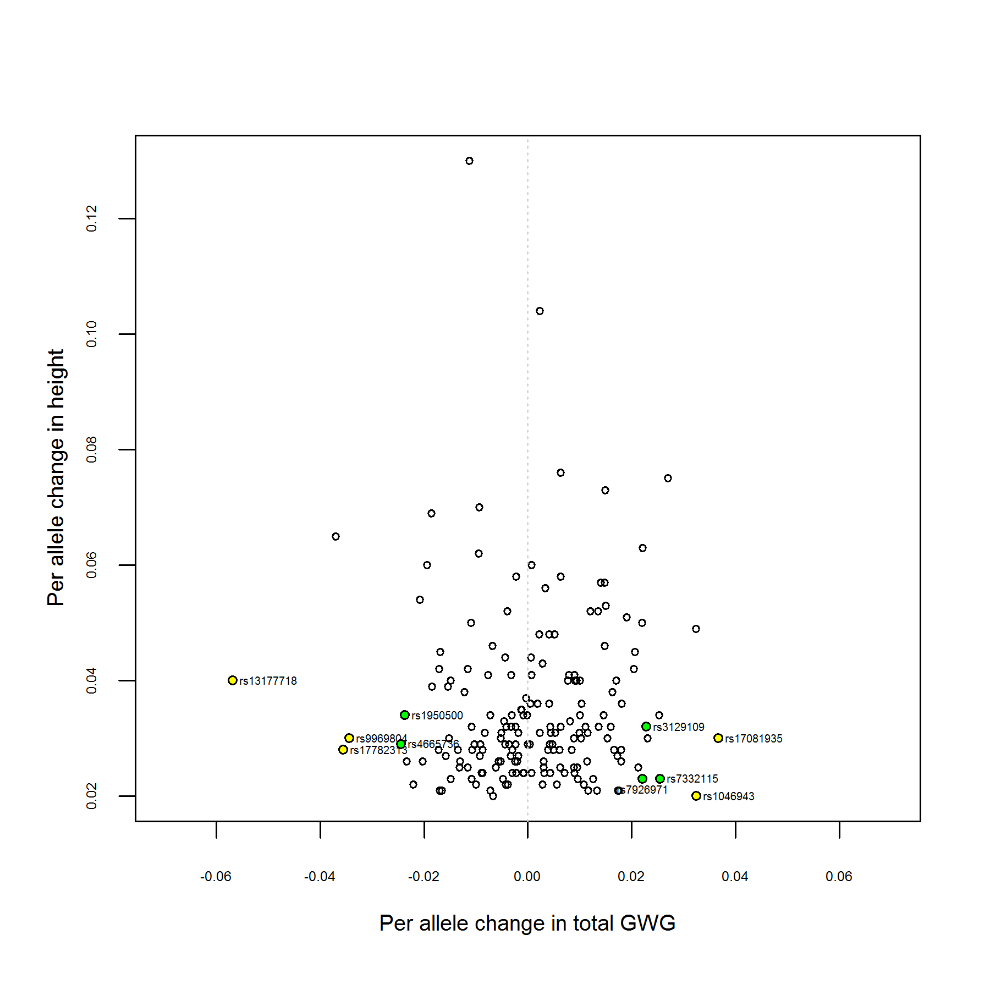** |

**Funding/support of individual studies**

***ALSPAC:*** The UK Medical Research Council and Wellcome Trust (102215/2/13/2) and the University of Bristol provide core support for ALSPAC. A Wellcome Trust (WT088806) grant provided funds for completion of genome wide (GW) genotyping on the ALSPAC mothers (WT088806). ALSPAC offspring GW data were generated by Sample Logistics and Genotyping Facilities at the Wellcome Trust Sanger Institute and LabCorp (Laboratory Corportation of America) using support from 23andMe. Maternal phenotypic and additional data collections were funded by the British Heart Foundation (SP/07/008/24066), WellcomeTrust (WT087997MA and WT092830M) and UK Medical Research Council (G1001357).

***DNBC-GOYA*:** This study is nested within the DNBC which was established with major grants from the Danish National Research Foundation, the Danish Pharmacists’ Fund, the Egmont Foundation, the March of Dimes Birth Defects Foundation, the Augustinus Foundation, and the Health Fund of the Danish Health Insurance Societies. The genotyping for DNBC-GOYA was funded by the Wellcome Trust (WT 084762).

***DNBC-PTB:*** This study is nested within the DNBC which was established and is maintained with major grants from the Danish National Research Foundation, the Danish Pharmacists’ Fund, the Egmont Foundation, the March of Dimes Birth Defects Foundation, the Augustinus Foundation, and the Health Fund of the Danish Health Insurance Societies. The generation of GWAS genotype data for the DNBC-PTB samples was carried out within the Gene Environment Association Studies (GENEVA) consortium with funding provided through the National Institutes of Health’s Genes, Environment, and Health Initiative (U01HG004423; U01HG004446; U01HG004438).

***EFSOCH:*** The Exeter Family Study of Childhood Health (EFSOCH) was supported by South West NHS Research and Development, Exeter NHS Research and Development, the Darlington Trust and the Peninsula National Institute of Health Research (NIHR) Clinical Research Facility at the University of Exeter. The opinions given in this paper do not necessarily represent those of NIHR, the NHS or the Department of Health.

***Gen3G:*** The Gen3G prospective cohort was supported by the Fonds de Recherche du Québec – Santé (FRQS – subvention Fonctionnement – Recherche Clinique - grant #20697) and by a Canadian Institute of Health Research (CIHR) Operating grant (Institute of Nutrition, Metabolism and Diabetes; MOP- 115071).

***Gen R:***  The general design of Generation R Study is made possible by ﬁnancial support from the Erasmus Medical Center, Rotterdam, the Erasmus University Rotterdam, the Netherlands Organization for Health Research and Development (ZonMw), the Netherlands Organisation for Scientific Research (NWO), the Ministry of Health, Welfare and Sport and the Ministry of Youth and Families. This research also received funding from the European Union’s Seventh Framework Programme (FP7/2007–2013), project EarlyNutrition under grant agreement n°289346. VWJ received an additional grant from the Netherlands Organization for Health Research and Development (VIDI 016.136.361) and a European Research Council Consolidator Grant (ERC-2014-CoG-648916). JFF has received funding from the European Union's Horizon 2020 research and innovation programme under grant agreement No 633595 (DynaHEALTH). This study was supported by funding from the European Union’s Horizon 2020 research and innovation programme (733206, LIFECYCLE).

***HAPO:*** This study was supported by the US National Institutes of Health (NIH) grants HD34242, HD34243 and HG004415.

***HIP:*** This study was funded by Tommy’s the Baby Charity and we acknowledge the support of the British Heart Foundation.

***INMA:*** This study was funded by grants from Instituto de Salud Carlos III (CB06/02/0041, FIS PI041436, PI081151, PI041705, PS09/00432, FIS-FEDER 03/1615, 04/1509, 04/1112, 04/1931 , 05/1079, 05/1052, 06/1213, 07/0314, 09/02647, 14/00891, 14/01687, PI06/0867, PI09/0009, MS13/00054), Spanish Ministry of Science and Innovation (SAF2013-49108-R), European Commission (ENGAGE project and grant agreement HEALTH-F4-2007-201413), Fundació La Marató de TV3, Generalitat de Catalunya-CIRIT AGAUR 2014 SGR-1138, Conselleria de Sanitat GeneralitatValenciana, Departamento de Sanidad del Gobierno Vasco (110093/2005, 110669/2009), DiputaciónForal de Gipuzkoa (DFG 05/007,DFG06/004, DFG08/001), Ayuntamiento de la Zona del estudio (Azpeitia, Azkoitia, Beasain, Zumarraga, Legazpia, Urretxu). Part of the DNA extractions and genotyping was performed at the Spanish National Genotyping Centre (CEGEN-Barcelona).

***MoBa:*** This work was supported by grants from the Norwegian Research Council (FUGE 183220/S10, FRIMEDKLI-05 ES236011), Swedish Medical Society (SLS 2008- 21198), Jane and Dan Olsson Foundations and Swedish government grants to researchers in the public health service (ALFGBG-2863, ALFGBG-11522), and the European Community’s Seventh Framework Programme (FP7/2007-2013). The Norwegian Mother and Child Cohort Study was also supported by the Norwegian Ministry of Health and the Ministry of Education and Research, NIH/NIEHS (contract no N01-ES-75558), NIH/NINDS (grant no.1 UO1 NS 047537-01 and grant no.2 UO1 NS 047537-06A1), and the Norwegian Research Council/FUGE (grant no. 151918/S10).

***NFBC1966 and NFBC1986:*** NFBC1966 and 1966 received financial support from the Academy of Finland (project grants 104781, 120315, 129269, 1114194, 24300796, Center of Excellence in Complex Disease Genetics and SALVE), University Hospital Oulu, Biocenter, University of Oulu, Finland (75617), NIHM (MH063706, Smalley and Jarvelin), Juselius Foundation, NHLBI grant 5R01HL087679-02 through the STAMPEED program (1RL1MH083268-01), NIH/NIMH (5R01MH63706:02), the European Commission (EURO-BLCS, Framework 5 award QLG1-CT-2000-01643), ENGAGE project and grant agreement HEALTH-F4-2007-201413, EU FP7 EurHEALTHAgeing -277849, the Medical Research Council, UK (G0500539, G0600705, G1002319, PrevMetSyn/SALVE) and the MRC, Centenary Early Career Award. The program is currently being funded by the H2020-633595 DynaHEALTH action and academy of Finland EGEA-project (285547).

***PANIC:*** The PANIC study was financially supported by grants from Ministry of Social Affairs and Health and Finland, Ministry of Education and Culture of Finland, Finnish Innovation Fund Sitra, Social Insurance Institution of Finland, Finnish Cultural Foundation, Juhu Vainio Foundation, Foundation for Pediatric Research, Paavo Nurmi Foundation, Paulo Foundation, Diabetes Research Foundation, Yrjo Jahnsson Foundation, Finnish Foundation for Cardiovascular Research, State research funding from Research Committee of Kuopio University Hospital Catchment Area, Kuopio University Hospital EVO funding, national doctoral programs, and the city of Kuopio.

***Raine:*** This study was supported by the National Health and Medical Research Council of Australia [grant numbers 572613, 403981 and 003209] and the Canadian Institutes of Health Research [grant number MOP-82893].

***Rhea:*** The Rhea study was financially supported by European projects (EU FP6-2003-Food-3-NewGeneris, EU FP6. STREP Hiwate, EU FP7 ENV.2007.1.2.2.2. Project No 211250 Escape, EU FP7-2008-ENV-1.2.1.4 Envirogenomarkers, EU FP7-HEALTH-2009- single stage CHICOS, EU FP7 ENV.2008.1.2.1.6. Proposal No 226285 ENRIECO, EU- FP7- HEALTH-2012 Proposal No 308333 HELIX), MeDALL (FP7 European Union project, No. 264357), and the Greek Ministry of Health (Program of Prevention of obesity and neurodevelopmental disorders in preschool children, in Heraklion district, Crete, Greece: 2011-2014; “Rhea Plus”: Primary Prevention Program of Environmental Risk Factors for Reproductive Health, and Child Health: 2012-15).

***STRIP:*** The STRIP study was financially supported by Academy of Finland (grants 206374 and 251360); Juho Vainio Foundation; Finnish Cardiac Research Foundation; Finnish Cultural Foundation; Finnish Ministry of Education and Culture; Sigrid Juselius Foundation; Yrjö Jahnsson Foundation; C.G. Sundell Foundation; Special Governmental Grants for Health Sciences Research, Turku University Hospital; Foundation for Pediatric Research; and Turku University Foundation

***SWS:*** The Southampton Women’s Survey was supported by grants from the Medical Research Council (MC_U147585827, MC_ST_U12055), British Heart Foundation (RG/07/009), Arthritis Research UK, National Osteoporosis Society, International Osteoporosis Foundation, Cohen Trust and NIHR Southampton Biomedical Research Centre, University of Southampton and University Hospital Southampton NHS Foundation Trust, with adjunctive support from the European Union's Seventh Framework Programme (FP7/2007-2013), project EarlyNutrition under grant agreement no. 289346.

***TEENAGE:*** This work was funded by the Wellcome Trust (098051) and has been co-financed by the European Union (European Social Fund—ESF) and Greek national funds through the Operational Program “Education and Lifelong Learning” of the National Strategic Reference Framework (NSRF)— Research Funding Program: Heracleitus II. Investing in knowledge society through the European Social Fund.

***Individual author funding:*** N.M.Warrington is supported by a National Health and Medical Research Council Early Career Fellowship (APP1104818); L. Paternoster was supported by a UK Medical Research Council fellowship (MR/J012165/1); MF. Hivert has received an American Diabetes Association (ADA) Pathways Accelerator Award (#1-15-ACE-26). D.A. Lawlor is a UK National Institute of Health Research Senior Investigator (NF-SI-0611-10196). R Beaumont and R Freathy are funded by the Wellcome Trust and Royal Society, grant 104150/Z/14/Z. A Hattersley is a Wellcome Trust Senior Investigator. B Jacobsson is funded by Jane and Dan Olsson Foundations (Gothenburg, Sweden), Swedish Medical Research Council (2015-02559), Norwegian Research Council/FUGE (grant no. 151918/S10; FRI-MEDBIO 249779) and March of Dimes (21-FY16-121), Swedish government grants to researchers in the public health service (ALFGBG-507701).

**Individual study acknowledgements:**

***ALSPAC:*** We are extremely grateful to all of the families who took part in this study, the midwives for their help in recruiting them, and the whole ALSPAC team, which includes interviewers, computer and laboratory technicians, clerical workers, research scientists, volunteers, receptionists, managers and nurses.

***DNBC-GOYA and DNBC-PTB:*** We are grateful to all of the families who have taken part in the DNBC, and the staff who have contributed to recruitment and data collection and analyses.

***EFSOCH:*** We are grateful to all Exeter Family Study participants and to all study personnel.

***Gen3G:*** Gen3G investigators would like to acknowledge the participants, the research staff from the endocrinology group at the Centre de Recherche clinique of the Centre Hospitalier de l’Université de Sherbrooke (CR-CHUS), the clinical and research staff of the Clinique de Prélèvement en Grossesse du CHUS, and the clinical staff from the CHUS Obstetric Department. The CR-CHUS is a FRQ-Sante affiliated research centre; recruitment and follow-up of Gen3G participants was possible based on the on-going support from the CR-CHUS.

***Gen R:*** The Generation R Study is conducted by the Erasmus Medical Center in close collaboration with the School of Law and Faculty of Social Sciences of the Erasmus University Rotterdam, the Municipal Health Service Rotterdam area, Rotterdam, the Rotterdam Homecare Foundation, Rotterdam and the Stichting Trombosedienst and Artsenlaboratorium Rijnmond (STAR-MDC), Rotterdam. We gratefully acknowledge the contribution of participating children and parents, general practitioners, hospitals, midwives and pharmacies in Rotterdam. The generation and management of GWAS genotype data for the Generation R Study were executed by the Human Genotyping Facility of the Genetic Laboratory of the Department of Internal Medicine, Erasmus MC, the Netherlands. We would like to thank Karol Estrada, Dr. Tobias A. Knoch, Anis Abuseiris, Luc V. de Zeeuw, and Rob de Graaf, for their help in creating GRIMP, BigGRID, MediGRID, and Services@MediGRID/D-Grid, (funded by the German Bundesministerium fuer Forschung und Technology; grants 01 AK 803 A-H, 01 IG 07015 G) for access to their grid computing resources. We thank Mila Jhamai, Manoushka Ganesh, Pascal Arp, Marijn Verkerk, Lizbeth Herrera and Marjolein Peters for their help in creating, managing and QC of the GWAS database. Also, we thank Karol Estrada for his support in creation and analysis of imputed data.

***GINIplus:*** The authors thank all the families for their participation in the GINIplus study. Furthermore, we thank all members of the GINIplus Study Group for their excellent work.

The GINIplus Study group consists of the following: Institute of Epidemiology I, Helmholtz Zentrum München, German Research Center for Environmental Health, Neuherberg (Heinrich J, Brüske I, Schulz H, Flexeder C, Zeller C, Standl M, Schnappinger M, Sußmann M, Thiering E, Tiesler C); Department of Pediatrics, Marien-Hospital, Wesel (Berdel D, von Berg A); Ludwig-Maximilians-University of Munich, Dr von Hauner Children’s Hospital (Koletzko S); Child and Adolescent Medicine, University Hospital rechts der Isar of the Technical University Munich (Bauer CP, Hoffmann U); IUF- Environmental Health Research Institute, Düsseldorf (Schikowski T, Link E, Klümper C).

***HAPO:*** HAPO would like to acknowledge the participants and research personnel at the participating HAPO field centres.

***HIP:*** We are grateful to the participants and the research midwives and other members of the research team. We also thank the Wellcome Trust Clinical Research Facility.

***INMA:*** The authors would like to thank all the participants for their generous collaboration. The authors are grateful to Silvia Fochs, Anna Sànchez, Maribel López, NuriaPey, Muriel Ferrer, AmparoQuiles, Sandra Pérez, Gemma León, Elena Romero, Maria Andreu, Nati Galiana, Maria Dolores Climent, Amparo Cases and Cristina Capo for their assistance in contacting the families and administering the questionnaires. A full roster of the INMA project investigators can be found at <http://www.proyectoinma.org/presentacion-inma/listado-investigadores/en_listado-investigadores.html>.

***LISAplus:*** The authors thank all the families for their participation in the LISAplus study. Furthermore, we thank all members of the LISAplus Study Group for their excellent work.

The LISAplus Study group consists of the following: Helmholtz Zentrum München, German Research Center for Environmental Health, Institute of Epidemiology I, Munich (Heinrich J, Schnappinger M, Brüske I, Sußmann M, Lohr W, Schulz H, Zeller C, Standl M); Department of Pediatrics, Municipal Hospital “St. Georg”, Leipzig (Borte M, Gnodtke E); Marien Hospital Wesel, Department of Pediatrics, Wesel (von Berg A, Berdel D, Stiers G, Maas B); Pediatric Practice, Bad Honnef (Schaaf B); Helmholtz Centre of Environmental Research – UFZ, Department of Environmental Immunology/Core Facility Studies, Leipzig (Lehmann I, Bauer M, Röder S, Schilde M, Nowak M, Herberth G , Müller J, Hain A); Technical University Munich, Department of Pediatrics, Munich (Hoffmann U, Paschke M, Marra S); Clinical Research Group Molecular Dermatology, Department of Dermatology and Allergy, Technische Universität München (TUM), Munich (Ollert M).

***MoBa:*** We are grateful to all the participating families in Norway who take part in this ongoing cohort study. Researchers interested in using MoBa data must obtain approval from the Scientific Management Committee of MoBa and from the Regional Committee for Medical and Health Research Ethics for access to data and biological material. Researchers are required to follow the terms of an Assistance Agreement containing a number of clauses designed to ensure protection of privacy and compliance with relevant laws.

***NFBC1966 and NFBC1986:*** The DNA extractions, sample quality controls, biobank up-keeping and aliquotting was performed in the National Public Health Institute, Biomedicum Helsinki, Finland and supported financially by the Academy of Finland and Biocentrum Helsinki. We thank the late Professor Paula Rantakallio (launch of NFBCs), and Ms Outi Tornwall and Ms Minttu Jussila (DNA biobanking). The authors would like to acknowledge the contribution of the late Academian of Science Leena Peltonen.

***PANIC:*** We are grateful to the parents and children for participating in the PANIC study and to the whole research team for their contribution in carrying out the study.

***Raine:*** The authors are grateful to the Raine Study participants and their families, and to the Raine Study research staff for cohort coordination and data collection. The authors gratefully acknowledge the NH&MRC for their long term contribution to funding the study over the last 25 years and also the following Institutions for providing funding for Core Management of the Raine Study: The University of Western Australia (UWA), Raine Medical Research Foundation, UWA Faculty of Medicine, Dentistry and Health Sciences, Telethon Kids Institute and Women and Infants Research Foundation and Curtin University. The authors gratefully acknowledge the assistance of the Western Australian DNA Bank (National Health and Medical Research Council of Australia National Enabling Facility).

This work was supported by resources provided by the Pawsey Supercomputing

Centre with funding from the Australian Government and the Government of

Western Australia.

***Rhea:*** The authors would like to thank all the participants for their generous collaboration.

***STRIP:*** We thank the study participants and the staff who have collected data.

***SWS:*** We thank the mothers of the Southampton Women's Survey who gave us their time and the team of dedicated research nurses and ancillary staff for their assistance.

***TEENAGE:*** We thank all study participants and their families as well as all volunteers for their contribution in this study. We thank the following staff from the Sample Management and Genotyping Facilities at the Wellcome Trust Sanger Institute for sample preparation, quality control and genotyping: Dave Jones, Doug Simpkin, Emma Gray, Hannah Blackburn, Sarah Edkins.

**Full list of EGG consortium members (as of Sep 2016) is provided below in alphabetical order.**

Linda S. Adair^1^, Tarunveer S. Ahluwalia^2,3,4^, Peter Almgren^5^, Wei Ang^6^, Mustafa Atalay^7^, Jonas Bacelis^8^, Robin N. Beaumont^9^, Jacques Beckmann^10^, Hans Bisgaard^2^, Thomas Bond^11^, Dorret I. Boomsma^12^, Judith B. Borja ^13,14^, Jonathan P. Bradfield^15^, Mariona Bustamante^16,17,18,19^, Alana Cavadino^20^, Lachlan Coin^21^, Cyrus Cooper^22^, Diana L. Cousminer^23,24,25^, Shikta Das^26^, Felix R. Day^27^, George V. Dedoussis^28^, Paul Elliott^11^, Johan G. Eriksson^29,30,31^, David M. Evans^32,33,34^, Bjarke Feenstra^35^, Janine F. Felix^36,37,38^,

Cilius E. Fonvig^3,39^, Timothy M. Frayling^9^, Rachel M. Freathy^9,32^, Romy Gaillard^37^, Frank Geller^35^, Matthew W. Gillman^40^, Struan F.A. Grant^15,24,41,42^, Niels Grarup^3^, Maria M. Groen-Blokhuis^43^, Leif Groop^5,23^, Monica Guxens^17,18,19^, Dexter Hadley^44^, Hakon Hakonarson^15,24,41^, Torben Hansen^3^, Andrew T. Hattersley^9^, M. Geoffrey Hayes^45,46,47^, Johannes Hebebrand^48^, Joachim Heinrich^49,50^, Øyvind Helgeland^51,52^, Tine B. Henrisksen^53^, Anke Hinney^48^, Joel Hirschhorn^54^, Marie-France Hivert^40,55^, Berthold Hoche^56,57,58^, John W.V. Holloway^59^, Jens-Christian Holm^3,39^, Momoko Horikoshi^60,61^, Jouke-Jan Hottenga^12^, Ville Huikari^62^, Elina Hyppönen^63,64,65^, Bo Jacobsson^66,67^, Vincent W.V. Jaddoe^36,37,38^, Marjo-Riitta Järvelin^62,68,69,70^, Stefan Johansson^51,71^, Marika Kaakinen^72^, Marjan Kerkhof^73^, Antje Körner^74,75^, Sailesh Kotecha^76^, Eskil Kreiner^2^, Benard Kulohoma^60^, Zoltán Kutalik^10,77^, Timo A. Lakka^7,78,79^, Debbie A. Lawlor^32,33^, Terho Lehtimäki^80,81^, Alexandra M Lewin^68^, Cecilia M Lindgren^60,82^, Virpi Lindi^7^, Allan Linneberg^83^, William L. Lowe Jr.^45^, Ronald C.W. Ma^84,85,86^, Aurelien Mace^87^, Reedik Mägi^88^, Per Magnus^66^, Anubha Mahajan^60^, Julie A. Marsh^6^, Mark I. McCarthy^60,61,89^, Niina S. McCarthy^90^, Mads Melbye^35,91,92^, Karen L. Mohlke^93^, Claire Monnereau^36,37,38^, Dennis O. Mook-Kanamori^94,95,96^, Andrew P. Morris^60,88,97^, Jeffrey C. Murray^98^, Ronny Myhre^66^, Harri Niinikoski^99,100^, Pål R. Njølstad^51^, Ellen Aagaard Nøhr^101^, Ioanna Ntalla^28,102^, Paul O'Reilly^103,104^, Emily Oken^40^, Ken K. Ong^27,105^, Katja Pahkala^106,107^, Kalliope Panoutsopoulou^108^, Oluf Pedersen^3^, Craig E. Pennell^6^, John R.B. Perry^27^, Pimphen Charoen^68,109^, Niina Pitkänen^106^, Beate St Pourcain^32,110^, Christine Power^63^, Rashmi Prasad^5^, Inga Prokopenko^60,72^, Olli T. Raitakari^106,111^, Christoph Reichetzeder^56,112^, Rebecca M. Reynolds^113^, Rebecca Richmond^32^, Alina Rodriguez^68,114^, Rany Salem^115,116,117,118^, Seang-Mei Saw^119,120^, Sylvain Sebert^62,69^, Verena Sengpiel^121^, Thorkild I.A. Sørensen^32,33,122,123^, Ulla Sovio^124^, Marie Standl^49^, Eric A.P. Steegers^125^, Evie Stergiakouli^32^, David P. Strachan^126^, Jordi Sunyer^17,18,19,127^, Yik-Ying Teo^119,128,129^, Elisabeth Thiering^49,130^, Nicholas J. Timpson^32,33^, Jessica Tyrrell^9,131^, André G. Uitterlinden^36,37,132^, Cornelia M. van Duijn^37^, Suzanne Vogelezang^36,37,38^, Tanja G.M. Vrijkotte^133^, Carol A. Wang^6^, Nicole M. Warrington^6,34^, William J. Watkins^76^, Erich Wichmann^49^, Elisabeth Widén^23^, Gonneke Willemsen^12^, James F. Wilson^134,135^, Hanieh Yaghootkar^9^, Mohammad H. Zafarmand^133^, Eleftheria Zeggini^108^

1. Department of Nutrition, University of North Carolina, Chapel Hill, NC 27599, USA.
2. COPSAC, Copenhagen Prospective Studies on Asthma in Childhood, Herlev and

Gentofte Hospital University of Copenhagen, Copenhagen, 2820 Gentofte, Denmark.

1. The Novo Nordisk Foundation Center for Basic Metabolic Research, Section of

Metabolic Genetics, Faculty of Health and Medical Sciences, University of Copenhagen, Copenhagen, DK -2100, Denmark.

1. Steno Diabetes Center, Gentofte, DK-2820, Denmark.
2. Department of Clinical Sciences, Diabetes and Endocrinology, Lund University

Diabetes Centre, Malmö, 205 02, Sweden.

1. Division of Obstetrics and Gynaecology, The University of Western Australia, Perth,

WA 6009, Australia.

1. Institute of Biomedicine, Physiology, University of Eastern Finland, Kuopio, FI-

70211, Finland

1. Department of Obstetrics and Gynecology, Sahlgrenska University Hospital,

Gothenburg, 41685, Sweden.

1. Institute of Biomedical and Clinical Science, University of Exeter Medical School,

University of Exeter, Royal Devon and Exeter Hospital, Exeter, EX2 5DW, UK.

1. Swiss Institute of Bioinformatics, Lausanne, CH-1015, Switzerland.
2. Department of Epidemiology and Biostatistics, School of Public Health, Imperial

College London, London, W2 1PG, UK.

1. Netherlands Twin Register, Department of Biological Psychology, Vrije Universiteit,

Amsterdam, 1081 BT, the Netherlands.

1. USC-Office of Population Studies Foundation, Inc., University of San Carlos, Cebu

City, 6000, Philippines.

1. Department of Nutrition and Dietetics, University of San Carlos, Cebu City, 6000,

Philippines.

1. Center for Applied Genomics, The Children’s Hospital of Philadelphia, Philadelphia,

PA 19104, USA.

1. Center for Genomic Regulation (CRG), Barcelona, 08003, Spain.
2. ISGlobal, Centre for Research in Environmental Epidemiology (CREAL), Barcelona,

08003, Spain.

1. Universitat Pompeu Fabra (UPF), Barcelona, 08002, Spain.
2. CIBER de Epidemiología y Salud Pública (CIBERESP), Madrid, 28029, Spain.
3. Centre for Environmental and Preventive Medicine, Wolfson Institute of Preventive

Medicine, Queen Mary University of London, London, EC1M 6BQ, UK.

1. Institute for Molecular Bioscience, University of Queensland, Brisbane, QLD 4072,

Australia.

1. MRC Lifecourse Epidemiology Unit, Faculty of Medicine, University of

Southampton, Southampton, SO16 6YD, UK.

1. Institute for Molecular Medicine, Finland (FIMM), University of Helsinki, Helsinki,

FI -00014, Finland.

1. Division of Human Genetics, The Children’s Hospital of Philadelphia, Philadelphia,

PA 19104, USA.

1. Department of Genetics, Perelman School of Medicine, University of Pennsylvania,

Philadelphia, PA 19104, USA.

1. Department of Primary Care and Public Health, Imperial College London,

Hammersmith, W6 6RP, UK.

1. MRC Epidemiology Unit, University of Cambridge School of Clinical Medicine,

Cambridge, CB2 0QQ, UK.

1. Department of Nutrition and Dietetics, School of Health Science and Education,

Harokopio University, Athens, 17671, Greece.

1. National Institute for Health and Wel fare, Helsinki, 00271, Finland.
2. Department of General Practice and Primary Health Care, University of Helsinki and

Helsinki University Hospital, Helsinki, 00014, Finland.

1. Folkhälsan Research Center, Helsinki, 00250, Finland.
2. Medical Research Council Integrative Epidemiology Unit at the University of Bristol,

Bristol, BS8 2BN, UK.

1. School of Social and Community Medicine, University of Bristol, Bristol, BS8 2BN,

UK.

1. The University of Queensland Diamantina Institute, Translational Research Institute,

Brisbane, QLD 4102, Australia.

1. Department of Epidemiology Research, Statens Serum Institut, Copenhagen, DK –

2300, Denmark.

1. The Generation R Study Group, Erasmus MC, University Medical Center Rotterdam,

Rotterdam, 3015 CE, the Netherlands.

1. Department of Epidemiology, Erasmus MC, University Medical Center Rotterdam,

Rotterdam, 3015 CE, the Netherlands.

1. Department of Pediatrics, Erasmus MC, University Medical Center Rotterdam,

Rotterdam, 3015 CE, the Netherlands.

1. The Children's Obesity Clinic, Department of Pediatrics, Copenhagen University

Hospital Holbæk, Holbæk, DK-4300, Denmark

1. Obesity Prevention Program, Department of Population Medicine, Harvard Medical

School and Harvard Pilgrim Health Care Institute, Boston, MA 02215, USA.

1. Department of Pediatrics, Perelman School of Medicine, University of Pennsylvania,

Philadelphia, PA 19104, USA.

1. Division of Endocrinology, The Children’s Hospital of Philadelphia, Philadelphia, PA

19104, USA.

1. Department of Biological Psychology, Vrije Universiteit Amsterdam, NCA

Neuroscience Campus Amsterdam, EMGO+ Institute for Health and Care Research,

Amsterdam, 1081 HV, the Netherlands.

1. Department of Pediatrics, University of California San Francisco School of Medicine,

San Francisco, CA 94158, USA.

1. Department of Medicine, Division of Endocrinology, Metabolism, and Molecular

Medicine, Northwestern University Feinberg School of Medicine, Chicago, IL 60611, USA.

1. Center for Genetic Medicine, Northwestern University Feinberg School of Medicine,

Chicago, IL 60611, USA.

1. Department of Anthropology, Northwestern University, Evanston, IL 60208, USA.
2. Department of Child and Adolescent Psychiatry, Psychosomatics and Psychotherapy,

University Hospital Essen, University of Duisburg-Essen, 45147 Essen, Germany.

1. Institute of Epidemiology I, Helmholtz Zentrum München -German Research Center

for Environmental Health, 85764 Neuherberg, Germany.

1. Institute and Outpatient Clinic for Occupational, Social and Environmental Medicine,

Inner City Clinic, University Hospital Munich, Ludwig Maximilian University of Munich, 80336 Munich, Germany.

1. KG Jebsen Center for Diabetes Research, Department of Clinical Science, University

of Bergen, Bergen, N-5020, Norway.

1. Department of Pediatrics, Haukeland University Hospital, Bergen, N-5021, Norway.
2. Department of Clinical Medicine - Department of Paediatrics, Aarhus University

Hospital, Aarhus N, 8200, Denmark.

1. Broad Institute of MIT and Harvard, Cambridge, MA 02142, USA.
2. Department of Medicine, Universite de Sherbrooke, Sherbrooke, QC J1H 5H3,

Canada.

1. Institute of Nutritional Sciences, University of Potsdam, D-14558 Potsdam, Germany.
2. IFLb GmbH - Institute for Laboratory Medicine, D-10627 Berlin, Germany.
3. Department of Basic Medicine, Medical College of Hunan Normal University,

Changsha, 46000, China.

1. Human Development and Health, Faculty of Medicine, University of Southampton,

Southampton, SO16 6YD, UK.

1. Wellcome Trust Centre for Human Genetics, University of Ox ford, Oxford, OX3

7BN, UK.

1. Oxford Centre for Diabetes, Endocrinology and Metabolism, University of Oxford,

Oxford, OX3 7LE, UK.

1. Biocenter Oulu, University of Oulu, Oulu, 90220, Finland.
2. Population, Policy and Practice, UCL Institute of Child Health, University College

London, London, WC1N 1EH, UK.

1. Centre for Population Health Research, School of Health Sciences, and Sansom

Institute, University of South Australia, Adelaide, SA 5001, Australia.

1. South Australian Health and Medical Research Institute, Adelaide, SA 5000,

Australia.

1. Norwegian Institute of Public Health, Oslo, N -0403, Norway.
2. Department of Obstetrics and Gynecology, Sahlgrenska Academy, University of

Gothenburg, Gothenburg, SE 41685, Sweden.

1. Department of Epidemiology and Biostatistics, MRC–PHE Centre for Environment &

Health, School of Public Health, Imperial College London, London, W2 1PG, UK.

1. Center for Life Course Health Research, Faculty of Medicine, University of Oulu,

Oulu, FI -90014, Finland.

1. Unit of Primary Care, Oulu University Hospital, Oulu, 90220, Finland.
2. Center for Medical Genetics and molecular Medicine, Haukeland University Hospital,

Bergen, N -5021, Norway.

1. Department of Genomics of Common Disease, School of Public Health, Imperial

College London, London, SW7 2AZ, UK.

1. Groningen Research Institute for Asthma and COPD (GRIAC), University of

Groningen, University Medical Center Groningen, Groningen, 9700 RB, the

Netherlands.

1. Pediatric Research Center, Department of Women ́s & Child Health, University of

Leipzig, 04103 Leipzig, Germany.

1. IFB Adiposity Diseases, University of Leipzig, 04103 Leipzig, Germany.
2. Department of Child Health, School of Medicine, Cardiff Univeristy, Cardiff, CF14

4XN, UK.

1. Institute of Social and Preventive Medicine, Lausanne University Hospital (CHUV),

Lausanne, 1010, Switzerland.

1. Department of Clinical Physiology and Nuclear Medicine, Kuopio University

Hospital, Kuopio, FI -70029, Finland.

1. Kuopio Research Institute of Exercise Medicine, Kuop io, FI-70100, Finland.
2. Department of Clinical Chemistry, Fimlab Laboratories, Tampere, 33520, Finland.
3. Department of Clinical Chemistry, University of Tampere School of Medicine,

Tampere, 33014, Finland.

1. Li Ka Shing Centre for Health Information and Discovery, The Big Data Institute,

University of Oxford, Oxford, OX3 7BN, UK.

1. Research Center for Prevention and Health Capital Region, Center for Sundhed,

Rigshospitalet – Glostrup, Copenhagen University, Glostrup, DK-2600, Denmark.

1. Department of Medicine and Therapeutics, The Chinese University of Hong Kong,

Hong Kong, China.

1. Li Ka Shing Institute of Health Sciences, The Chinese University of Hong Kong,

Hong Kong, China.

1. Hong Kong Institute of Diabetes and Obesity, The Chines e University of Hong

Kong, Hong Kong, China.

1. Institute of Social and Preventive Medicine, University Hospital of Lausanne,

Lausanne, 1010, Switzerland.

1. Estonian Genome Center, University of Tartu, Tartu, 50090, Estonia.
2. Oxford National Institute for Health Research (NIHR) Biomedical Research Centre,

Churchill Hospital, Oxford, OX3 7LE, UK.

1. Centre for Genetic Origins of Health and Disease (GOHaD), The University of

Western Australia, Crawley, WA 6009, Australia.

1. Department of Clinical Medicine, University of Copenhagen, Copenhagen, DK-2200,

Denmark.

1. Department of Medicine, Stanford School of Medicine, Stanford, California, CA

94305, USA.

1. Department of Genetics, University of North Carolina, Chapel Hill, NC 27599, USA.
2. Department of Clinical Epidemiology, Leiden University Medical Center, Leiden,

2333 ZA, the Netherlands.

1. Department of Public Health and Primary Care, Leiden University Medical Center,

Leiden, 2333 ZA, the Netherlands.

1. Epidemiology Section, BESC Department, King Faisal Specialist Hospital and

Research Centre, Riyadh, 12713, Saudi Arabia.

1. Department of Biostatistics, University of Liverpool, Liverpool, L69 3GA, UK.
2. Department of Pediatrics, University of Iowa, Iowa City, IA 52242, USA.
3. Department of Pediatrics, Turku University Hospital, Turku, 20014, Finland.
4. Department of Physiology, University of Turku, Turku, 20520, Finland.
5. Research Unit for Gynaecology and Obstetrics, Department of Clinical Research,

University of Southern Denmark, Odense, 5230, Denmark.

1. William Harvey Research Institute, Barts and the London School of Medicine and

Dentistry, Queen Mary University of London, London, EC1M 6BQ, UK.

1. Institute of Psychiatry, Kings College London, London, SE5 8AF, UK.
2. Medical Research Council (MRC), Social, Genetic and Developmental Psychiatry

Centre, London, SE5 8AF, UK.

1. Department of Paediatrics, University of Cambridge, Cambridge, CB2 0QQ, UK.
2. Research Centre of Applied and Preventive Cardiovascular Med icine, University of

Turku, Turku, FI-20014, Finland.

1. Paavo Nurmi Centre, Sports and Exercise Medicine Unit, Department of Physical

Activity and Health, Turku, FI-20014, Finland.

1. Wellcome Trust Sanger Institute, Hinxton, CB10 1HH, UK.
2. Department of Tropical Hygiene, Faculty of Tropical Medicine, Mahidol University,

Bangkok, 10400, Thailand.

1. Max Planck Institute for Psycholinguistics, Nijmegen, 6525 XD, the Netherlands.
2. Department of Clinical Physiology and Nuclear Medicine, Turku University Hospital,

Turku, 20520, Finland.

1. Center for Cardiovascular Research, Charité - Universitätsmedizin Berlin, 10117

Berlin, Germany.

1. BHF Centre for Cardiovascular Science, University of Edinburgh, Queen's Medical

Research Institute, Edinburgh, Scotland, EH16 4TJ, UK.

1. Department of Psychology, Mid Sweden University, Östersund, 83125, Sweden.
2. Division of Endocrinology, Department of Medicine, Boston Children's Hospital,

Boston, MA 02115, USA.

1. Department of Genetics, Harvard Me dical School, Boston, MA 02115, USA.
2. Center for Basic and Translational Obesity Research, Boston Children's Hospital,

Boston, MA 02115, USA.

1. Program in Medical and Population Genetics, Broad Institute of Harvard and MIT,

Cambridge, MA 02142, USA.

1. Saw Swee Hock School of Public Health, National University of Singapore, National

University Health System, Singapore, 119077, Singapore.

1. Singapore Eye Research Institute, Singapore, 168751, Singapore.
2. Department of Obstetrics and Gynecology, Sahlgrenska University Hospital,

Gothenburg, SE-41685, Sweden.

1. Novo Nordisk Foundation Center for Basic Metabolic Research and Department of

Public Health, Faculty of Health and Medical Sciences, University of Copenhagen, Copenhagen, DK -2200, Denmark.

1. Institute of Preventive Medicine, Bispebjerg and Frederiksberg Hospital, The Capital

Region, Copenhagen, DK-2000, Denmark.

1. Department of Obstetrics and Gynaecology, University of Cambridge, National

Institute for Health Research Cambridge Comprehensive Biomedical Research Centre, Cambridge, CB2 0SW, UK.

1. Department of Obstetrics and Gynecology, Erasmus MC, University Medical Center,

Rotterdam, 3015 CE, the Netherlands.

1. Population Health Research Institute, St George's University of London, London,

Cranmer Terrace, SW17 0RE, UK.

1. IMIM (Hospital del Mar Medical Research Institute), Barcelona, 08003, Spain.
2. Department of Statistics and Applied Probability, National University of Singapore,

Singapore, 117546, Singapore.

1. Life Sciences Institute, National University of Singapore, Singapore, 117456,

Singapore.

1. Division of Metabolic and Nutritional Medicine, Dr. von Hauner Children's Hospital,

University of Munich Medical Center, 80337 Munich, Germany.

1. European Centre for Environment and Human Health, University of Exeter, Truro,

TR1 3HD, UK

1. Department of Internal Medicine, Erasmus MC, University Medical Center

Rotterdam, 3015 CE, the Netherlands.

1. Department of Public Health, Academic Medical Center (AMC), University of

Amsterdam, Amsterdam, 1100 DD, the Netherlands.

1. Usher Institute for Population Health Sciences and Informatics, University of

Edinburgh, Edinburgh, Scotland, EH8 9AG, UK.

1. MRC Human Genetics Unit, Institute of Genetics and Molecular Medicine, University

of Edinburgh, Edinburgh, Scotland, EH4 2XU, UK

**eReferences**

1. Boyd A, Golding J, Macleod J, et al. Cohort Profile: the 'children of the 90s'--the index offspring of the Avon Longitudinal Study of Parents and Children. *International journal of epidemiology* 2013; **42**(1): 111-27.

2. Fraser A, Macdonald-Wallis C, Tilling K, et al. Cohort Profile: The Avon Longitudinal Study of Parents and Children: ALSPAC mothers cohort. *Int J Epidemiol* 2013; **42**(1): 97-110.

3. Fraser A, Tilling K, Macdonald-wallis C, et al. Association of maternal weight gain in pregnancy with offspring obesity and metabolic and vascular traits in childhood. *Circulation* 2010; **121**: 2557-64.

4. Paternoster L, Evans DM, Nohr EA, et al. Genome-wide population-based association study of extremely overweight young adults--the GOYA study. *PLoS One* 2011; **6**(9): e24303.

5. Olsen J, Melbye M, Olsen SF, et al. The Danish National Birth Cohort--its background, structure and aim. *ScandJPublic Health* 2001; **29**(4): 300-7.

6. Knight B, Shields BM, Hattersley AT. The Exeter Family Study of Childhood Health (EFSOCH): study protocol and methodology. *PaediatrPerinatEpidemiol* 2006; **20**(2): 172-9.

7. Guillemette L, Allard C, Lacroix M, et al. Cohort profile: Genetics of Glucose regulation in Gestation and Growth (Gen3G) – a prospective pre-birth cohort of mother-child pairs in Sherbrooke, Canada. . *BMJ open* 2016; **6**(2): e010031.

8. Kooijman MN, Kruithof CJ, van Duijn CM, et al. The Generation R Study: design and cohort update 2017. *European journal of epidemiology* 2016; **31**(12): 1243-64.

9. Berg A, Kramer U, Link E, et al. Impact of early feeding on childhood eczema: development after nutritional intervention compared with the natural course - the GINIplus study up to the age of 6 years. *Clin Exp Allergy* 2010; **40**(4): 627-36.

10. Metzger BE, Lowe LP, Dyer AR, et al. Hyperglycemia and adverse pregnancy outcomes. *N Engl J Med* 2008; **358**(19): 1991-2002.

11. Guxens M, Ballester F, Espada M, et al. Cohort Profile: The INMA--INfancia y Medio Ambiente--(Environment and Childhood) Project. *International journal of epidemiology* 2011.

12. Heinrich J, Bolte G, Holscher B, et al. Allergens and endotoxin on mothers' mattresses and total immunoglobulin E in cord blood of neonates. *Eur Respir J* 2002; **20**(3): 617-23.

13. Magnus P, Birke C, Vejrup K, et al. Cohort Profile Update: The Norwegian Mother and Child Cohort Study (MoBa). *Int J Epidemiol* 2016.

14. Magnus P, Irgens LM, Haug K, et al. Cohort profile: the Norwegian Mother and Child Cohort Study (MoBa). *Int J Epidemiol* 2006; **35**(5): 1146-50.

15. Nilsen RM, Vollset SE, Gjessing HK, et al. Self-selection and bias in a large prospective pregnancy cohort in Norway. *Paediatr Perinat Epidemiol* 2009; **23**(6): 597-608.

16. Irgens LM. The Medical Birth Registry of Norway. Epidemiological research and surveillance throughout 30 years. *Acta Obstet Gynecol Scand* 2000; **79**(6): 435-9.

17. Williams LA, Evans SF, Newnham JP. Prospective cohort study of factors influencing the relative weights of the placenta and the newborn infant. *BMJ* 1997; **314**: 1864-8.

18. Chatzi L, Plana E, Daraki V, et al. Metabolic syndrome in early pregnancy and risk of preterm birth. *Am J Epidemiol* 2009; **170**(7): 829-36.

19. Horikoshi M, Yaghootkar H, Mook-Kanamori DO, et al. New loci associated with birth weight identify genetic links between intrauterine growth and adult height and metabolism. *Nat Genet* 2013; **45**(1): 76-82.

20. Speliotes EK, Willer CJ, Berndt SI, et al. Association analyses of 249,796 individuals reveal 18 new loci associated with body mass index. *Nat Genet* 2010; **42**(11): 937-48.

21. Heid IM, Jackson AU, Randall JC, et al. Meta-analysis identifies 13 new loci associated with waist-hip ratio and reveals sexual dimorphism in the genetic basis of fat distribution. *Nat Genet* 2010; **42**(11): 949-60.

22. Lango Allen H, Estrada K, Lettre G, et al. Hundreds of variants clustered in genomic loci and biological pathways affect human height. *Nature* 2010; **467**(7317): 832-8.

23. Ehret GB, Munroe PB, Rice KM, et al. Genetic variants in novel pathways influence blood pressure and cardiovascular disease risk. *Nature* 2011; **478**(7367): 103-9.

24. Johnson T, Gaunt TR, Newhouse SJ, et al. Blood pressure loci identified with a gene-centric array. *Am J Hum Genet* 2011; **89**(6): 688-700.

25. Dupuis J, Langenberg C, Prokopenko I, et al. New genetic loci implicated in fasting glucose homeostasis and their impact on type 2 diabetes risk. *Nat Genet* 2010; **42**(2): 105-16.

26. Morris AP, Voight BF, Teslovich TM, et al. Large-scale association analysis provides insights into the genetic architecture and pathophysiology of type 2 diabetes. *Nat Genet* 2012; **44**(9): 981-90.

27. Wang TJ, Zhang F, Richards JB, et al. Common genetic determinants of vitamin D insufficiency: a genome-wide association study. *Lancet* 2010; **376**(9736): 180-8.
